# Artificial intelligence in intimate partner violence risk pathways: a PRISMA-ScR review of femicide prevention and medico-legal accountability

**DOI:** 10.3389/fdgth.2026.1877651

**Published:** 2026-06-23

**Authors:** Paolo Bailo, Giulio Nittari, Tommaso Spasari, Filippo Gibelli, Giovanna Ricci

**Affiliations:** 1 Section of Legal Medicine, School of Law, University of Camerino, Camerino, Italy; 2 Telemedicine and Telepharmacy Centre, School of Medicinal and Health Products Sciences, University of Camerino, Camerino, Italy; 3 Section of Occupational and Legal Medicine and BioLaw, Niccolò Cusano University, Rome, Italy

**Keywords:** artificial intelligence, decision support, digital health, femicide, human oversight, intimate partner violence, medico-legal accountability, risk assessment

## Abstract

**Introduction:**

Artificial intelligence (AI), machine learning, natural language processing and related decision-support methods are increasingly studied in intimate partner violence (IPV), domestic-violence and gender-based violence contexts. The key question is not whether AI can predict femicide as an individual lethal event, but whether AI-related methods may help institutions recognise, document, communicate and act on distributed signs of escalation across clinical, legal, police, social-service and digital settings.

**Methods:**

This PRISMA-ScR scoping review, informed by Joanna Briggs Institute guidance and structured using the Population-Concept-Context framework, mapped English-language AI-related literature in IPV, domestic violence, coercive-control and femicide-related risk pathways. Sexual violence was included only when embedded in IPV, domestic-abuse, coercive-control, family-violence, lethality-risk or femicide-related pathways.

**Results:**

Searches identified 4,099 records; after deduplication, 2,906 were screened, 166 reports were assessed at full text and 125 were included in the core evidence map. The evidence was heterogeneous, spanning clinical and electronic health records, police narratives, legal documents, social media or online posts, survey data, linked administrative data and survivor-facing digital tools. AI-related methods were used mainly for detection, classification, record linkage, risk stratification, text mining, triage or decision support rather than for direct evaluation of femicide-prevention interventions. Femicide, lethality and severe escalation were addressed in only part of the corpus, and few studies examined implementation, human oversight, false reassurance, fairness, privacy or downstream institutional action in depth.

**Discussion:**

The findings do not support individual femicide prediction or demonstrate that AI prevents lethal violence. Instead, they support a more defensible role for AI as a bounded component in human-led risk-recognition pathways. The review develops a six-layer conceptual synthesis linking distributed risk signals, AI-assisted signal processing, human contextual review, multi-agency response, legal-ethical governance and medico-legal accountability. AI may support institutional recognition and coordination, but it cannot substitute for professional judgment, survivor-centred practice, due process or adequately resourced prevention systems.

## 1 Introduction

Violence against women, including intimate partner violence (IPV), remains a major public-health, human-rights and medico-legal concern. International and European sources describe IPV as associated with physical injury, mental-health harm, sexual and reproductive consequences, social isolation and economic vulnerability, and they document the continuing burden of gender-based violence across jurisdictions (1–4). At the most severe end of this continuum, intimate partner and family-related femicides represent gender-related killings that frequently arise in contexts of prior abuse, coercive control, threats, separation, stalking, weapon access, previous police contact, healthcare encounters or social-service involvement (5–8). These observations do not make lethal outcomes predictable in an individual deterministic sense. They do, however, raise a medico-legal question that is both narrower and more concrete: when signs of escalation are available across institutional settings, how are they recognised, documented, communicated and acted upon?

The medico-legal relevance of this problem does not lie in claiming that lethal violence can be forecast with certainty. It lies in the relationship between foreseeability, documentation, escalation and response. Femicide should be framed as a possible endpoint of pathways of violence, coercive control and institutional contact, not as a mechanically predictable event. Risk assessment and risk management guidance in the IPV field already emphasises lethality risk, repeated victimisation, escalation and safety planning without collapsing these into deterministic prediction (7, 9). Domestic homicide and death-review sources likewise point to missed opportunities, information silos, incomplete risk management and implementation failures rather than to a total absence of warning signs (10–12). For forensic and legal medicine, the central question is therefore whether danger was reasonably recognisable and whether institutions responded in a proportionate, documented and coordinated way.

Healthcare is one important part of this distributed pathway. World Health Organization (WHO) guidance emphasises survivor-centred first-line support, safe enquiry, confidentiality, referral and health-system organisation rather than actuarial certainty (13–15). Emergency departments, primary care, mental-health services, obstetric and gynaecological settings and injury-related encounters may all generate records relevant to violence, fear or repeated harm. At the same time, police calls, prior complaints, protection-order proceedings, social-service referrals, shelter contact and digital abuse reports may sit in separate institutional systems. The warning signal, if present, is therefore often fragmented. No single professional may see the full chronology, and no single dataset can safely represent the lived dynamics of coercive control.

Artificial intelligence (AI)-related methods have attracted interest because they may process unstructured records, identify patterns in text, support linkage across systems or prompt triage and review. Studies in the included corpus have applied machine learning or natural language processing (NLP) to legal documents concerning intimate partner femicide and to police narrative data (16, 17). Other work has examined clinical notes, electronic health records (EHRs) and broader institutional records relevant to IPV identification (18, 19). These methods may contribute to detection, record organisation or prioritisation of professional review. Their promise, however, is often easier to state than to implement safely. The institutional data available for modelling are incomplete, shaped by under-reporting, affected by structural inequality and sensitive in ways that raise privacy, discrimination and surveillance concerns.

That heterogeneity makes conceptual precision necessary. Prediction, risk assessment, risk stratification, early warning, decision support and prevention are not interchangeable. Prediction implies a forecast that a particular future event will occur. Risk assessment evaluates known risk factors and case context. Risk stratification assigns relative priority categories. Early warning identifies signals requiring professional review. Decision support assists reasoning but does not replace it. Prevention requires action: safety planning, referral, protection, perpetrator management, follow-up and inter-agency coordination. These distinctions matter because a low algorithmic score may create false reassurance, while a high score may prompt intrusive or discriminatory intervention if not embedded in meaningful human review. A low score should therefore be interpreted only as the absence of a model-generated alert under specified data conditions, not as evidence that danger is absent. Conversely, a high score should trigger proportionate professional review rather than automatic coercive, intrusive or discriminatory action.

This review maps AI-related methods studied in IPV, domestic violence, coercive-control and femicide-related risk contexts. It asks not whether AI can predict femicide, but how AI-related methods have been used across risk-recognition pathways and what those uses imply for prevention, governance and medico-legal accountability. Existing AI/IPV literature is too heterogeneous to support a single technical conclusion, and only a subset of studies addresses femicide, intimate partner homicide or lethality directly (20, 21). This supports a scoping-review approach and a cautious conceptual synthesis. In this article, femicide-prevention implications are used in a specific and limited sense: femicide is not treated as an individual prediction endpoint, and AI is not presented as a substitute for risk assessment, clinical judgement, police decision-making, judicial oversight or social-service response. Rather, the analysis examines whether AI-related methods may support earlier institutional stages at which risk signals are recorded, missed, linked or escalated, while preserving professional responsibility and avoiding false reassurance, discriminatory surveillance and technological solutionism.

## 2 Methods

### 2.1 Study design, eligibility criteria and population-concept-context (PCC) framework

This study was conducted as a PRISMA-ScR scoping review and was informed by JBI guidance for scoping-review methodology (22, 23). A scoping approach was selected because the field is broad, methodologically heterogeneous and not yet suited to meta-analysis or a narrow effectiveness review. The review protocol was not prospectively registered because the review was exploratory and iterative, as is common in scoping work that aims to map an emerging interdisciplinary field. Nevertheless, the eligibility framework, screening process, data charting and reporting were structured according to PRISMA-ScR and JBI principles throughout. The objective was to map how AI-related methods have been studied in IPV, domestic violence, coercive-control and femicide-related risk contexts, and to examine the implications of this evidence for medico-legal and public-health prevention pathways.

Eligibility was structured using the Population-Concept-Context (PCC) framework, which is recommended in JBI scoping-review methodology for clarifying broad review questions and organising heterogeneous evidence. The population domain was defined inclusively as any person exposed to IPV, domestic violence, domestic abuse, family violence, coercive control, gender-based violence, violence against women, stalking in an IPV/domestic-abuse context, intimate partner homicide, domestic homicide, femicide or lethality risk. The review recognised that most retrieved publications focused on women and girls, but eligibility was not limited to cisgender women. Same-sex relationships, transgender relationships and LGBTQ + populations were considered within scope where the violence occurred within an intimate-partner or domestic-abuse pathway. For this review, IPV was operationally understood as violence, abuse, coercive control, stalking or lethality-related risk occurring in current or former intimate relationships, including current or former spouses, partners, cohabiting or non-cohabiting partners, boyfriends/girlfriends and ex-boyfriends/ex-girlfriends.

The concept domain included artificial intelligence, machine learning, deep learning, natural language processing, large language models, algorithmic or automated decision-making, predictive analytics, data mining, text mining, risk prediction, decision support, clinical decision support, risk stratification, early-warning systems, record linkage, data linkage and EHR-based identification. The context domain included healthcare, emergency care, primary care, mental health, police or law enforcement, courts or judicial settings, emergency calls, social services, shelters, digital platforms and multi-agency prevention systems. Reports were included in the empirical evidence map only when all three domains were present: a relevant violence context, an AI-related or algorithmic technology, and a function related to detection, screening, classification, risk assessment, risk stratification, triage, decision support, referral, prevention, inter-agency coordination, medico-legal accountability or legal/ethical governance. Records were excluded from the core evidence map when they addressed general violence, offender-risk prediction, child abuse, elder abuse, generic digital health or commercial tools without a clear IPV/femicide risk-pathway and AI-related component. Records addressing sexual violence were included only when sexual violence was situated within intimate partner violence, domestic abuse, coercive control, family violence, lethality risk or femicide-related pathways. Studies focused exclusively on non-partner sexual assault, sexual-violence forensics, child sexual exploitation or sexual-offence detection without an IPV/domestic-abuse risk-pathway component were outside the scope.

### 2.2 Information sources, search strategy and selection process

The empirical search covered biomedical, public-health, social-science, criminology and computing-oriented databases, including PubMed/MEDLINE, Scopus, Web of Science, PsycINFO, Criminal Justice Abstracts, IEEE Xplore and ACM Digital Library. Searches covered all years available in each database up to 28 April 2026 and were limited to English-language records. Search strings combined terms for IPV, domestic violence, coercive control, femicide, intimate partner homicide and gender-based violence with terms for AI, machine learning, NLP, predictive analytics, decision support, risk stratification, record linkage and related technologies. Database-specific search strategies are provided in Supplementary Table S4. Searches were intentionally sensitive because preliminary testing showed that the narrower expression “AI and femicide” would miss relevant work on IPV risk pathways and institutional data sources. Reference-list and backward citation checking were also used to identify additional potentially relevant records.

**Figure 1:**
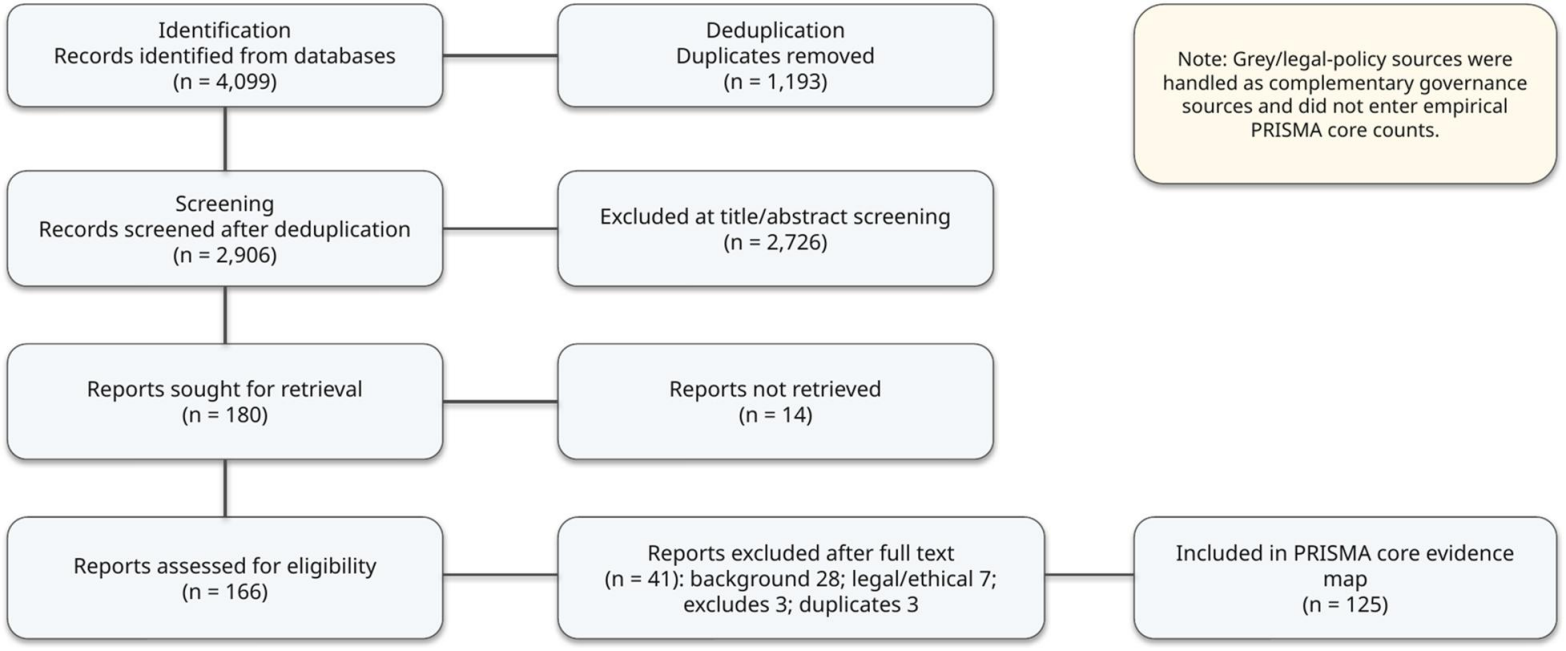
PRISMA-ScR flow diagram. The figure summarises identification, deduplication, screening, full-text retrieval and inclusion in the PRISMA core evidence map. Complementary background/contextual and legal/ethical sources are shown outside the empirical core.

After deduplication, 2,906 records were screened at title and abstract level by two reviewers using the eligibility criteria described above. Records were separated into likely core evidence, background/contextual material, legal/ethical complementary material or exclusions. Full texts were sought for 180 reports. Full-text assessment of the 166 retrieved reports was performed primarily by two reviewers, with support from the author group. Disagreements or uncertain classifications were resolved by consensus among all authors. Fourteen reports could not be retrieved despite legal and institutional access attempts. These reports are listed separately in Supplementary Table S5 on the basis of bibliographic metadata and titles only; because their full texts were unavailable, they were not assessed at full-text level and were not incorporated into the core evidence map. The final core evidence map included 125 reports/studies. Twenty-eight background/contextual sources and seven legal/ethical complementary sources were retained outside the empirical core; three duplicate or variant records were not counted separately. The PRISMA-ScR flow is summarised in Figure 1.

### 2.3 Data charting, evidence synthesis and complementary legal-policy sources

Data charting captured bibliographic information, PCC classification, country or region where available, study type, violence focus, population, relationship status where reported, institutional setting, data source, AI or digital technology type, intended function, target outcome, validation status, performance metrics when reported, femicide/lethality relevance, coercive-control relevance, repeat-victimisation or severe-escalation relevance, and discussion of ethical or governance issues. One reviewer performed the main data charting, and all records were checked and refined with support from the author group. Governance fields included false positives, false negatives, false reassurance, algorithmic bias, discrimination, surveillance risk, privacy or data protection, explainability, human oversight, data minimisation, auditability, institutional responsibility and medico-legal accountability. The expanded record-level PCC classification and data-charting matrix is provided in Supplementary Table S2. Categories were not always mutually exclusive; for example, a single study could involve healthcare data, text mining and risk stratification. Consequently, setting and function are reported descriptively and, where counts are provided, as non-mutually-exclusive mappings rather than as mutually exclusive percentages.

A complementary grey/legal-policy corpus was used to support interpretation of governance, institutional responsibility and public-health implications. These sources included official legal instruments and institutional guidance, including General Data Protection Regulation (GDPR), the European Union (EU) AI Act, Directive (EU) 2024/1385 and the Istanbul Convention (24–27). They also included European Data Protection Board (EDPB) guidance on automated decision-making, data protection impact assessment, privacy by design and AI-model governance (28–31), together with public-health, Group of Experts on Action against Violence against Women and Domestic Violence (GREVIO) and domestic-homicide review sources relevant to risk management, coordination and institutional learning (32–34). These sources were not included in the empirical PRISMA core and did not alter the review counts. They informed the legal, ethical, public-health and medico-legal interpretation of the mapped evidence.

### 2.4 Conceptual synthesis development

The review did not include a formal risk-of-bias or quality appraisal because the purpose was to map the breadth, characteristics and conceptual implications of a heterogeneous evidence base rather than estimate the effectiveness of an intervention. Synthesis was descriptive and thematic. We first mapped technologies, data sources, settings and intended functions; then identified where femicide, lethality, severe escalation, governance and medico-legal issues appeared in the corpus. The initial conceptual synthesis was developed from the charted data and then discussed and refined with all authors. The six layers were identified through thematic analysis of recurring implementation gaps, oversight requirements and accountability points across the empirical corpus, and were then mapped along a risk-recognition pathway from distributed warning signals to institutional response and post-event accountability. This synthesis was interpreted in light of the complementary legal and policy sources. It is not presented as a validated instrument, prediction model, operational checklist, clinical guideline or police tool.

## 3 Results

### 3.1 Study selection and characteristics of included records

The search identified 4,099 raw records. After deduplication, 2,906 records were screened. Full texts were sought for 180 reports, 166 were assessed and 125 were included in the PRISMA core evidence map (Figure 1). The included PRISMA-ScR core records are listed in Supplementary Table S1, and the record-level PCC classification and data-charting matrix is reported in Supplementary Table S2. Table 1 provides a compact summary of the main study characteristics using descriptive, non-mutually-exclusive categories. The included corpus covered a wide range of publication types, including model-development studies, validation studies, observational studies using administrative or clinical data, text-mining studies, social-media or online-community analyses, digital intervention evaluations, conference papers, reviews and conceptual or governance-oriented contributions. This breadth confirms that the field is not organised around one technology, one dataset or one outcome.

**Table 1:** Study characteristics summary of the PRISMA-ScR core evidence map.

Domain	Non-mutually exclusive categories in the core evidence map	n/125 where safely coded	Interpretive note
Population/violence focus	Domestic violence/abuse; intimate partner violence; gender-based violence/violence against women; family violence; femicide/feminicide; stalking; intimate partner/domestic homicide; lethality or severe escalation; coercive control	Domestic violence/abuse 81; IPV 54; gender-based violence/violence against women 27; family violence 15; femicide/feminicide 9; stalking 7; intimate partner/domestic homicide 5; lethality/severe escalation 4; coercive control 4	Most records addressed IPV or domestic violence pathways rather than femicide as a direct endpoint.
AI/digital method	Machine learning; predictive analytics/modeling; deep learning; AI; NLP/text mining; EHR-based identification; data mining; record/data linkage; LLM/generative AI; decision support/risk stratification	Machine learning 73; predictive analytics/modeling 44; deep learning 37; AI 33; NLP/text mining 31; EHR/electronic health records 15; data mining 14; record/data linkage 8; LLM/generative AI 6; decision support/risk stratification 4	Methods were often combined within a single study.
Intended function	Detection/identification; prevention/intervention/referral; prediction/forecasting; risk assessment/stratification; screening; early warning/surveillance; legal/ethical governance; decision support/triage; inter-agency coordination	Detection/identification 112; prevention/intervention/referral 58; prediction/forecasting 56; risk assessment/stratification 35; screening 19; early warning/surveillance 10; legal/ethical governance 10; decision support/triage 8; inter-agency coordination 2	Functions describe intended or discussed use, not demonstrated prevention effectiveness.
Data source/context	Healthcare and EHR data; police/law-enforcement records; legal/judicial documents; online communities and social-media data; survey or population-level data; linked administrative datasets; survivor-facing digital tools	Detailed record-level coding is provided in Supplementary Table S2.	Contexts were classified descriptively because a single record could involve multiple data sources or settings.
Femicide/lethality relevance	Femicide/feminicide; intimate partner homicide/domestic homicide; lethality or severe escalation	14 records had direct femicide, homicide or lethality/severe-escalation coding.	The remaining records contribute indirectly through IPV/domestic-violence risk-pathway evidence.
Validation/implementation status	Model development/validation; model development/evaluation; clinical or implementation trial; observational/data-linkage; qualitative; review/meta-analysis; survey/cross-sectional; protocol, case study, conceptual/technical or unclear	Model development/validation 59; model development/evaluation 12; clinical/implementation trial 7; observational/data-linkage 4; qualitative 13; review/meta-analysis 11; survey/cross-sectional 5; protocol/case/conceptual/unclear 14	Technical feasibility was more common than downstream implementation or outcome evaluation.

Counts are descriptive and non-mutually exclusive except where study-design categories were coded as mutually exclusive in the supplementary evidence map. Detailed record-level PCC classification is provided in Supplementary Table S2.

**Table 2:** AI functions across IPV risk pathways and femicide-prevention implications.

AI-related function	Typical data sources/settings	Pathway contribution	Key limits	Required human oversight
Detection and screening	EHRs, clinical notes, coded records, emergency or primary-care settings	May help flag possible IPV-related presentations or under-coded abuse histories for professional review.	Risk of under-detection, poor coding, confounding and false reassurance when records are incomplete.	Outputs should prompt contextual assessment, not automatic labelling or action.
NLP and text mining	Police narratives, legal documents, clinical notes, online posts	Can extract patterns, injuries, abuse types, coercive-control indicators or help-seeking signals from unstructured text.	Narratives reflect institutional language, reporting patterns and missing context; semantic accuracy does not equal safety impact.	Professionals must interpret outputs in light of chronology, survivor account and service context.
Record linkage and data linkage	Health, police, registry, administrative or multi-agency data	May reveal distributed contacts and repeated victimisation across systems.	High privacy burden; linkage errors; risks of disproportionate surveillance and unclear lawful basis.	Strict purpose limitation, data minimisation, governance review and accountable access controls.
Risk prediction and stratification	Survey data, police/clinical datasets, risk factors, prior violence indicators	May stratify cases for review or identify combinations associated with severe harm.	Model performance is not equivalent to individual femicide prediction or validated prevention.	Scores must be advisory, auditable and overridden when contextual information indicates risk.
Digital triage and decision support	Clinical workflows, service platforms, safety-planning or referral systems	May structure documentation, triage prompts, referral and follow-up.	A prompt without a response pathway is not prevention; poorly designed systems may increase workload.	Clear escalation protocols, training, referral capacity and accountability for action/inaction.
Survivor-facing digital tools	Chatbots, online support, help-seeking platforms	May support access to information or referral when safe and accessible.	Digital safety, perpetrator monitoring, confidentiality and unequal access are major concerns.	Tools must preserve autonomy, privacy and safe exit options; human support should be available.

IPV, intimate partner violence; EHR, electronic health record; NLP, natural language processing. The table summarises mapped functions and conceptual implications; it does not imply demonstrated femicide-prevention effectiveness.

The included records focused primarily on IPV, domestic violence or family violence rather than femicide as a direct endpoint. A smaller subset addressed intimate partner femicide, intimate partner homicide, lethality risk, severe escalation or domestic homicide review-type concerns. This distribution is important for interpretation: the evidence base is relevant to femicide-prevention implications because femicide may occur at the end of escalating IPV trajectories, but most included studies were not designed to evaluate femicide prevention directly. The corpus therefore supports a risk-pathway interpretation rather than an event-prediction interpretation.

Because scoping reviews aim to map rather than rank evidence, the included records were interpreted as a landscape of uses rather than as a hierarchy of effectiveness. Several records were methodological or model-development contributions, while others were exploratory, implementation-oriented or review-based. The field is consequently best understood as an emerging set of partial applications. Some studies show that relevant signals may be extracted from text or structured records; others show that these signals are difficult to translate into practice without careful attention to workflow, safety, confidentiality and professional responsibility. This distinction is important because a technically successful classifier does not automatically become a prevention mechanism.

### 3.2 Technologies, data sources and intended functions

The technologies mapped in the review included machine-learning classifiers, deep-learning approaches, NLP pipelines, large-language-model applications, text and data mining, decision-support tools, record linkage, EHR-based screening or identification, social-media classification, chatbot-supported help-seeking and risk-prediction or risk-stratification models. Police and legal-text studies used NLP or text mining to identify abuse types, injuries, coercive-control behaviours, mental-health mentions, recorded-crime features, future offending or femicide-related case characteristics (16, 17, 35–38). Closely related work examined police-record surveillance outputs, linkage between police and health records, and NLP-assisted improvement of police-recorded domestic-abuse data (39–42). Healthcare-oriented studies used diagnostic codes, clinical notes, electronic health records, emergency-department narratives, dental or injury presentations and linked administrative records to identify IPV or related harms (18, 19, 43, 44). These studies differ substantially in purpose: some make hidden information easier to retrieve, others develop risk or classification models, and others explore digital support or help-seeking needs.

A further pattern concerned the distinction between data extraction and decision support. Some NLP and text-mining studies sought to improve the visibility of IPV-related information already present in narrative records, including police narratives, clinical notes and online disclosures (45–48). This is a relatively bounded use: the technology helps make existing institutional knowledge more searchable or structured. Other studies moved closer to risk prediction, risk stratification or prioritisation, including models for court or legal processes, police risk assessment, recidivism and domestic-violence vulnerability (48–51). These raise more difficult questions because the output may influence how cases are ranked, reviewed or escalated. Extracting an IPV circumstance from a clinical note, classifying abuse type in a police narrative, and assigning a relative risk category to a person or case have different implications for consent, transparency, error, proportionality and reviewability. The mapped literature did not always distinguish these functions clearly. This conceptual imprecision is itself a finding of the review.

The data sources differed in their proximity to institutional action. Clinical notes and diagnostic records are relevant to screening and referral, but they may not capture coercive control, fear, threats or perpetrator access. EHR-based studies ranged from identification through diagnoses and phone calls to association-rule mining and older-women health correlates (19, 43, 44, 52). Other clinical and emergency-care studies addressed traumatic-brain-injury prescreening, hospital identification, dental or injury presentations and mental-health record classification (53–56). Additional emergency and clinical narratives further showed how much depends on documentation practices and the reason for contact (46, 47, 57, 58). Police narratives may contain more detailed event descriptions, but they reflect reporting and recording practices. Linked police-health data and primary-care or family-law linkage studies show the value of institutional linkage, while also exposing the legal and ethical difficulty of bringing separate systems together (41, 59). Social media and online forums can show help-seeking, disclosure and distress, yet they raise privacy and representativeness problems (60–62). Survivor-facing chatbots and digital platforms may reduce barriers to information, but their safety depends on usability, confidentiality, perpetrator monitoring risk and the availability of human services after disclosure (63–65). Across these sources, the review found no evidence that more data alone produces safer outcomes.

Across these technological groups, validation and interpretability were uneven. Some studies reported conventional discrimination metrics, but fewer addressed calibration, transportability across jurisdictions, or performance in settings different from the development dataset. This matters because IPV-related data are strongly shaped by local documentation practices, legal definitions, service access and reporting behaviour. Predictive-modelling studies in domestic violence and violence-against-women contexts used approaches ranging from multimodal machine learning and ensemble learning to XGBoost and feature-selection approaches (66–68). Other studies examined machine learning for violence-against-women classification, IPV measurement, domestic-violence vulnerability and prevalence or incidence estimation (69–72). Further work developed perpetrator-focused, multidimensional victimisation or feature-explanation models across different populations and jurisdictions (73–76). Such studies are important for mapping technical possibilities, but they do not remove the need for external validation, calibration and implementation testing. A model trained on police narratives in one jurisdiction may not translate to another jurisdiction with different recording rules or risk-assessment procedures. Similarly, EHR-based approaches may perform differently in emergency care, primary care, mental health or obstetric settings because the opportunity to ask about violence, the language used by clinicians and the coding practices vary. The review therefore supports a cautious interpretation of technical performance: reported accuracy or area-under-the-curve (AUC) values should be read as evidence of feasibility within a defined dataset, not as evidence that a tool is ready for deployment in femicide-prevention pathways.

A related limitation is that validation was often separated from the conditions under which tools would be used. A model may perform adequately on a retrospective dataset and still fail when deployed in a setting where documentation is incomplete, disclosure is unsafe, staff have little time to interpret outputs, or referral options are unavailable. Some studies explicitly engaged with fairness, interpretability, feature selection or risk-factor explanation, but these concerns were not consistently integrated into workflow, survivor safety or accountability analysis (48, 49, 75). Other population-level models estimated domestic violence, family violence or IPV vulnerability across geographical contexts, including Türkiye, Liberia and Iran, and across population or situational contexts, including homelessness among young adults and COVID-19-related conditions (77–81). These records broaden the empirical map but also reinforce the need to separate model-development value from implementation readiness. The review therefore suggests that implementation evidence should be treated as part of the evidentiary standard, not as a later technical detail. In this field, the relevant question is not only whether a system identifies IPV-related information, but whether the resulting prompt can be reviewed, contextualised and acted upon without increasing risk.

### 3.3 Femicide-prevention implications, governance issues and evidence gaps

Only part of the corpus addressed femicide, intimate partner homicide, lethality or severe escalation directly. These studies are important because they show that AI-related methods can be applied to legal documents, homicide-related data, pregnancy-associated homicide, attempted femicide, news alert detection or multilevel risk-factor modelling (16, 82). Related femicide- or homicide-oriented records included systematic, data-mining and participatory-alert approaches (21, 83–85). Reviews and applied studies also show growing interest in machine learning for femicide detection or IPV-prevention claims, but this literature remains heterogeneous and unevenly connected to real-world safety outcomes (20, 86). The corpus also includes risk-related work on antepartum IPV, stalking and indicators in coded records, which are relevant to escalation pathways but should not be treated as femicide-prediction instruments (87–89). These studies do not establish that individual femicide events can be predicted reliably. Nor do they demonstrate that algorithmic outputs prevent lethal violence. The more consistent finding is that AI-related methods may help organise large volumes of fragmented information, identify patterns in unstructured text, support case-linkage or prompt further professional review. These are pathway functions rather than proof of prevention.

The governance content of the empirical corpus was often less developed than the modelling content. The main ethical, legal and medico-legal safeguards for human-governed AI-supported IPV risk pathways are summarised in Table 3. Many studies reported technical performance but said less about how an output should be interpreted, who should review it, how disagreement should be handled, what level of uncertainty is acceptable, or how survivors' autonomy and safety should be protected. Few studies examined how professionals respond to AI prompts in real settings, whether alerts are ignored, whether low-risk outputs reduce vigilance, or whether high-risk outputs produce intrusive action. False positives and false negatives were often implicit rather than analysed as institutional risks. False negatives may create reassurance where contextual assessment should continue, while false positives may expose survivors or families to unnecessary intrusion, stigma or coercive interventions. Bias and fairness were inconsistently examined, despite the likelihood that IPV reporting, healthcare access, policing, language, migration status, disability, socioeconomic position and race or ethnicity may influence recorded data. Studies of police risk assessment, interpretable recidivism modelling and disengagement from legal proceedings illustrate that fairness, explanation and procedural consequences are not optional add-ons when algorithmic outputs may affect institutional action (48–50).

**Table 3:** Ethical, legal and medico-legal safeguards for AI-supported IPV risk pathways.

Issue	Why it matters in IPV/femicide-prevention pathways	Safeguard or implication
False negatives and false reassurance	A low-risk output may discourage escalation despite coercive control, threats, stalking or separation risk.	Low-risk scores should never substitute for contextual assessment or professional judgment.
False positives and intrusive intervention	Over-classification can stigmatise survivors, trigger unwanted intervention or increase surveillance burdens.	Use proportionate triage, documented review and survivor-centred response options.
Algorithmic bias and discrimination	Data reflect reporting inequalities, policing practices, healthcare access and socioeconomic vulnerability.	Assess fairness, under-protection and over-intervention across groups before deployment.
Privacy and data minimisation	IPV pathways may involve health, sexual, family, location and justice-related data.	Limit processing to necessary variables, purposes and access rights; conduct DPIA where required.
Human oversight	Automated outputs may shape institutional priority or protective responses.	Oversight must be meaningful, informed and able to challenge, override or ignore the output.
Explainability and auditability	Professionals need to understand why an alert was generated and what action followed.	Maintain interpretable outputs, logs, escalation records and reviewable decision trails.
Institutional accountability	Responsibility cannot be displaced to a model, vendor or score.	Post-event review should examine recognition, documentation, communication, escalation and action/inaction.
Legal governance	GDPR, the EU AI Act, Directive (EU) 2024/1385 and Istanbul Convention/GREVIO materials constrain use.	Treat AI as bounded support within lawful, proportionate, human-led and coordinated service pathways.
Automation bias	Professionals may over-trust or passively accept AI outputs because they appear objective or technically authoritative.	Training, workflow design and audit should require active contextual review, documentation of disagreement and the ability to override both high-risk and low-risk outputs.
Automated decisions and high-risk AI governance	Outputs may affect access to referral, shelter assessment, protection, policing priority or healthcare triage.	Avoid solely automated significant decisions; ensure lawful basis, meaningful human review, contestability and, where applicable, EU AI Act high-risk safeguards.

The safeguards are derived from the review synthesis and complementary legal/policy corpus. They are not a validated implementation protocol.

The evidence gaps were especially pronounced for outcome evaluation. Very few records connected AI-supported identification, triage or help-seeking tools to measurable changes in referral, safety planning, protection, perpetrator management, follow-up, survivor-reported outcomes, severe escalation or lethal harm. Survivor-facing digital studies, chatbot evaluations, large-language-model applications and GraphRAG studies shift attention beyond model performance, but they remain early applications rather than validated response pathways (45, 60, 63–65, 90, 91). The corpus therefore cannot support claims that AI prevents femicide. It shows instead where AI-related methods may be studied more responsibly: recognition of distributed signals, structuring of unstructured records, linkage of repeated contacts, prioritisation for human review, documentation of reasoning and auditability of institutional response. For a medico-legal and public-health readership, the key distinction is that prevention is not achieved when a record is classified; it begins only when classification is translated into safe enquiry, professional review, referral, protection, follow-up or coordinated action.

## 4 Conceptual synthesis: from AI tools to accountable risk pathways

The included corpus does not point toward a single dominant technology, outcome definition or implementation pathway. Instead, it shows a fragmented field in which AI-related methods are used for detection, classification, linkage, triage, decision support or risk stratification across different institutional contexts. A conceptual synthesis is therefore useful not to create a linear pipeline or a new risk score, but to locate these methods within the wider sequence of risk recognition, documentation, escalation, response and accountability. This is particularly important because IPV cases are dynamic and institutional information may be partial, delayed or contradictory. A survivor may disclose in one setting and deny abuse in another because of fear, dependence, migration status, disability, children, financial control, digital coercion, spyware, perpetrator monitoring or the risk that the perpetrator will discover the disclosure or associated digital traces. A police record may describe an incident without capturing the longer pattern of coercion, while a healthcare record may document injuries without recording the relationship context. Distributed signals are therefore fragments requiring contextual interpretation rather than data points that automatically produce a decision.

**Figure 2:**
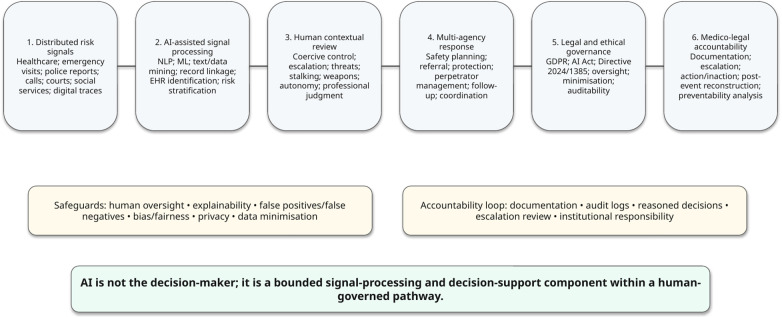
Conceptual synthesis of AI-supported IPV risk pathways. The figure organises the mapped evidence into six layers: distributed risk signals, AI-assisted signal processing, human contextual review, multi-agency response, legal and ethical governance, and medico-legal accountability. AI is represented as a bounded signal-processing component rather than as the decision-maker; digital coercion, automation-bias safeguards, legal governance and medico-legal accountability are addressed in the accompanying synthesis text.

We interpret the evidence through a six-layer conceptual synthesis of AI-supported IPV risk pathways. This synthesis is not a validated intervention, risk score, clinical guideline, police tool or operational checklist. Its purpose is to organise the mapped evidence and clarify where AI-related methods may assist, and where human interpretation, institutional responsibility and legal safeguards remain decisive. The first layer consists of distributed risk signals. These may appear in healthcare encounters, emergency visits, police reports, emergency calls, judicial documents, social-service contacts, shelter referrals or digital contexts. Such signals may include repeated injuries, fear, threats, stalking, coercive control, separation, pregnancy-related vulnerability, child-related risk, weapon access, repeated calls or fragmented prior complaints. The scoping review suggests that AI methods may be relevant because many of these signals are embedded in unstructured or dispersed records rather than in one clean dataset. The six-layer synthesis is summarised in Figure 2.

The second layer is AI-assisted signal processing. This is the only layer in which algorithmic methods are centrally located. NLP, machine learning, text mining, data linkage, EHR-based identification and risk stratification may help process large volumes of information or prompt review. Yet this layer remains bounded. It does not determine what happened, what risk means in context, or what response is proportionate. Studies using police narratives, EHRs, linked datasets, social-media posts and survivor-facing digital tools show the range of potential signal-processing functions (36, 40, 59, 92). Other model-development studies show that risk stratification can be technically explored across populations and contexts, but they also demonstrate why a model output cannot be equated with a preventive decision (69–72).

The third layer is human contextual review. At this stage, algorithmic outputs must be interpreted against chronology, survivor voice, coercive-control context, missing information, professional knowledge and proportionality. Human review is therefore not a procedural formality after signal processing, but the point at which uncertainty, context and possible action are assessed. This layer must also guard against automation bias: without training and institutional safeguards, professionals may over-trust low-risk or high-risk outputs because they appear objective, technical or authoritative.

The fourth layer is multi-agency response. Risk recognition has preventive value only if it connects to action: safe enquiry, referral, safety planning, protection measures, perpetrator management, follow-up and coordination across health, police, courts and social services. A signal without a response pathway is not prevention. This layer is supported more by public-health, risk-management and legal-policy sources than by AI-performance studies, because it concerns organisational capacity, not model accuracy (26, 27). Public-health and monitoring sources likewise emphasise implementation, coordination and risk management as institutional duties rather than algorithmic outputs (32–34). It is also where survivor autonomy and digital safety become central. A tool that prompts disclosure without safe referral, confidentiality or follow-up may increase documentation without increasing protection.

The fifth layer is legal and ethical governance: lawfulness, transparency, data minimisation, privacy, explainability, fairness, auditability and safeguards against discriminatory or disproportionate use define the conditions under which AI-supported pathways may be acceptable. The sixth layer is medico-legal accountability. This layer concerns how warning signs, AI-generated alerts, missed alerts, suppressed or overridden prompts, uncertainty, missing information, professional reasoning, escalation decisions and institutional responses are documented in a way that supports continuity, institutional learning and post-event reconstruction. It is the layer at which AI methods become relevant not as accountable actors, but as traceable elements within a reviewable human and institutional decision pathway.

This synthesis deliberately resists technological solutionism. AI is only one bounded component within a pathway between distributed warning signs and human, multi-agency, legally accountable action. The conceptual value of the synthesis lies in separating signal processing from contextual judgment, judgment from response, and response from accountability. It also requires any AI-supported proposal to specify which layer it addresses and which layers remain outside the model. A text-mining tool may help identify abuse-related information, but it does not decide whether a survivor is safe. A linkage model may show repeated contacts across systems, but it does not create a multi-agency response. A chatbot may provide information or classify help-seeking needs, but it cannot replace confidential advocacy or emergency protection (63, 64, 90). A predictive classifier may rank cases, but it cannot determine whether an intervention is lawful, proportionate or acceptable to the survivor (49, 51). The same framework disciplines the language of prevention: most AI-related work in the included corpus belongs, at most, to secondary or tertiary prevention, supporting recognition of existing or escalating violence, but it does not address the structural determinants of gender-based violence and does not by itself provide shelter, legal protection, economic support or perpetrator accountability. AI-supported outputs must therefore remain contestable, interpretable and subordinate to professional reasoning, particularly in coercive-control contexts where visible injury may be absent and disclosure may be constrained by fear or surveillance.

## 5 Discussion

This scoping review maps a growing but heterogeneous body of literature on AI-related methods in IPV and domestic-violence risk contexts. The principal finding is not that AI can predict femicide, nor that AI-supported tools have been shown to prevent lethal violence. Rather, the evidence shows that AI-related methods are being applied to parts of the institutional ecology in which IPV risk is recorded: clinical notes, EHRs, police narratives, legal documents, linked administrative data, social media and digital help-seeking environments. These applications are relevant because femicide-prevention work often depends on earlier recognition of escalation, but they remain several steps removed from demonstrating prevention. The strongest contribution of the field is therefore organisational rather than deterministic: AI may help institutions process, link or prioritise information that would otherwise remain fragmented.

A useful way to interpret these findings is to separate epistemic value from preventive value. AI may have epistemic value when it helps reveal patterns in records that are otherwise difficult to review at scale. This is particularly relevant for narrative police records, EHR notes and linked administrative data. Preventive value requires more. It depends on whether the signal reaches a trained person, whether that person has time and authority to act, whether services are available, whether survivor safety is protected, and whether the action taken is proportionate. Much of the existing literature stops at the epistemic level. It asks whether a model can detect, classify or stratify. The harder question is whether such detection changes what institutions do next.

Individual femicide prediction is a scientifically fragile and ethically dangerous frame because femicide is a low-base-rate, context-dependent and socially embedded outcome. A model trained on partial institutional data may appear technically impressive while still being vulnerable to under-reporting, missingness, selection bias and proxy discrimination. More importantly, even a statistically plausible risk output does not answer the practical questions on which prevention depends: who reviews the case, what information is missing, how survivor autonomy is protected, what response is available, and how the perpetrator's behaviour is managed. Risk-pathway support is more defensible because it asks AI to assist with limited tasks—such as flagging records for review, extracting IPV-related circumstances from text, linking repeated contacts or supporting triage—while leaving contextual interpretation and action to accountable human systems.

False reassurance is a particularly important medico-legal risk. A low or absent risk flag may be misread as absence of danger, especially in busy clinical, police or social-service environments. This risk is not merely technical; it concerns institutional behaviour. If professionals rely on a low-risk output despite threats, stalking, strangulation, separation, weapon access, repeated contacts or survivor fear, the problem is not only model error but also a failure of contextual judgment. False positives also matter. Over-identification can expose survivors to unwanted interventions, unnecessary surveillance, loss of trust, or escalation by perpetrators if digital or institutional actions are not handled safely. A responsible system must therefore treat both false negatives and false positives as safety and rights issues, not as abstract performance metrics.

The analysis also cautions against a victim-only risk lens. Femicide prevention cannot be reduced to identifying high-risk victims. It must also address perpetrator behaviour, coercive control, threats, stalking, weapon access, breaches of orders, separation dynamics and institutional capacity to intervene. Tools that focus only on the victim's records may miss the relational and behavioural nature of danger. This has medico-legal significance because preventability is rarely a property of one data point. It concerns the chronology of warning signs, the behaviour of the perpetrator, the response of institutions and the availability of protection. Studies of perpetrator-focused prediction, IPV perpetration among homeless young adults, stalking and legal-process disengagement illustrate why the unit of analysis should often be the pathway or case context rather than the victim alone (50, 74, 78, 88). AI-supported approaches should therefore be evaluated not only for their ability to classify victim vulnerability, but also for their capacity to support broader case understanding, perpetrator management and inter-agency coordination.

Bias, surveillance and discrimination are similarly central. IPV data are not neutral. They reflect who reports, who is believed, who accesses healthcare, how police narratives are written, which injuries are coded, and which communities are over- or under-policed. Algorithmic systems trained on such data may reproduce under-protection of some groups and over-intervention in others. The use of digital traces, online posts or linked institutional datasets may further increase the risk of disproportionate monitoring, particularly for migrants, racialised women, disabled women, LGBTQ + survivors or those economically dependent on perpetrators. Social-media and online-community studies are useful for detecting expressions of distress or help-seeking, but they also make clear that digital data are context-dependent, unevenly representative and ethically sensitive (93, 94). Deep-learning studies using online posts, Facebook-based datasets, Persian-language social-media content and other online-text corpora extend this map but do not resolve the underlying representativeness and privacy concerns (95–97). These risks do not mean that AI-related methods should never be studied; they mean that any implementation must be narrow, transparent, proportionate and accountable.

The legal and policy corpus reinforces this conclusion. GDPR principles limit indiscriminate data aggregation and require lawfulness, fairness, transparency, purpose limitation, minimisation, accuracy and accountability (24). Special-category data and high-risk processing raise additional safeguards (24, 29, 30). Depending on design and effect, automated decision-making safeguards may be relevant, particularly if outputs influence access to protection, policing priority or institutional action (28). The EU AI Act adds a risk-based governance frame, including human oversight and, where applicable, fundamental-rights impact assessment logic (25). EDPB guidance on AI models and data protection by design further supports a cautious approach to lawfulness, fairness, minimisation and downstream use (30, 31). Directive (EU) 2024/1385, the Istanbul Convention and GREVIO materials shift the focus toward prevention, protection, support, training, coordination and risk management rather than technological prediction alone (26, 27, 33, 34). These sources do not prohibit every AI-supported pathway, but they make clear that deployment in this field would require strict governance and cannot be justified by general claims about innovation or efficiency.

A concrete example illustrates why these safeguards matter. If a hospital AI-supported triage system classified a survivor as “low risk” and that output automatically reduced access to specialist IPV referral, shelter assessment, protection planning or professional review, the issue would no longer be mere technical prioritisation. It would concern automated decision-making with potentially legal or similarly significant effects, requiring a clear lawful basis, meaningful human intervention, contestability, documentation and safeguards against false reassurance. GDPR Article 22 may be relevant where decisions are based solely on automated processing and produce legal or similarly significant effects, while broader GDPR principles remain relevant even when a human remains in the loop (24, 28). Depending on intended purpose, use context and effect, some law-enforcement triage, victim-risk assessment, emergency-call prioritisation or healthcare triage systems may also fall within the EU AI Act's high-risk governance logic, including human oversight and fundamental-rights safeguards where applicable (25). Not every IPV-related AI system will automatically fall into these categories, but systems that shape access to protection, policing priority or medical prioritisation require particularly strict justification and oversight.

A further implication concerns the relationship between digital health and legal accountability. Digital systems can create records of alerts, overrides, missing information and decisions. Properly governed, such logs may support continuity and institutional learning. Poorly governed, they may create defensive documentation, automation bias or confusion about who is responsible. In medico-legal reconstruction, an ignored alert may be relevant, but so may blind reliance on a reassuring output. Studies of linked police-health data, clinical-record mining and text-mined police narratives show how digital traces can make institutional knowledge more visible, but visibility is not the same as accountability (39, 41, 42, 54). Accountability should therefore be understood as a design requirement, not only as a post-event process. Systems should be built so that professionals can explain why a signal was generated, how it was reviewed, what contextual information was considered, and why a particular action was or was not taken.

The medico-legal contribution of this review lies in reframing foreseeability. Foreseeability is not deterministic prediction. In post-event reconstruction, the relevant questions are whether warning signs were available, how they were documented, whether information was communicated, whether escalation was recognised, whether actions were proportionate and whether institutional procedures were followed. AI-generated alerts, logs or risk flags may become evidentially relevant in this reconstruction, but they cannot become the accountable actor. Responsibility remains with institutions and professionals who design, deploy, interpret and act—or fail to act—within the pathway. This is why the conceptual synthesis links prevention to accountability: an early-warning signal is meaningful only when it is embedded in a documented, reviewable and resourced response system.

Future research should move beyond model performance alone and test whether AI-supported prompts can be understood, governed and acted upon in real service pathways. Studies need external validation, fairness assessment, calibration, human-factors evaluation, survivor acceptability, digital-safety analysis and implementation research. Outcome evaluation should examine whether AI-supported tools change professional behaviour, referral, case conferencing, safety planning, perpetrator management or continuity of care, while also measuring false reassurance, intrusive false positives, privacy harms, discrimination, workload, interpretability and institutional response (67, 70, 75, 77). For digital health, the implication is that this field should not be framed as algorithmic prediction of rare lethal events, but as responsible infrastructure for safer, more consistent, better documented and less fragmented human action. Work on IPV surveillance, prevalence estimation and coded-record indicators shows that measurement and identification remain necessary research tasks, but these tasks must be connected to service pathways if they are to have preventive significance (73, 80, 89). Without these conditions, AI may increase documentation without improving safety, or produce alerts that institutions cannot meaningfully act upon.

The policy implications are consequently modest but important. Institutions considering AI-supported IPV tools should begin with the response pathway, not with the model. They should define what problem the tool addresses, who will review its outputs, what safeguards apply, what actions are available, how survivor autonomy will be protected and how errors will be audited. They should also avoid introducing systems that merely shift responsibility to frontline workers without providing resources, training or service capacity. In this field, the ethical test of a digital system is not whether it can produce a score, but whether it contributes to safer, more accountable and less fragmented care and protection. Integrative and systematic reviews of AI in IPV prevention already point to similar concerns about heterogeneity, limited implementation evidence and the need for human oversight; the present review extends this by foregrounding femicide-prevention implications and medico-legal accountability (20, 86).

## 6 Strengths and limitations

This review has several strengths. It maps an interdisciplinary evidence base across health, digital health, criminology, policing, legal-document analysis, online support and AI governance. It uses a transparent PRISMA-ScR process, retains all included core studies in Supplementary Material, separates empirical evidence from complementary legal and policy sources, and adds a record-level PCC classification matrix to improve traceability of the corpus. It also brings a medico-legal perspective to a field often framed primarily as technical prediction or digital innovation. By citing representative studies across clusters while retaining the complete set of 125 records in Supplementary Tables S1, S2, the review avoids both under-documenting the corpus and turning the article into a catalogue.

The limitations are equally important. The included records were highly heterogeneous in design, data source, outcome definition and technical maturity, precluding meta-analysis. The predominance of studies focused on IPV or domestic violence generally, rather than femicide, lethality or severe escalation specifically, should be interpreted as a characteristic of the corpus with important inferential limits rather than as evidence that the review can support femicide-event prediction. The review supports a risk-pathway interpretation: femicide-prevention implications are derived from literature on recognition, documentation, linkage and escalation of IPV-related signals, not from direct evidence that AI predicts or prevents lethal outcomes. The review also does not map the broader AI literature on sexual violence, sexual-assault forensics or non-partner sexual victimisation. Its focus is narrower: AI-related methods in IPV and domestic-abuse risk pathways, where sexual violence may be one component of coercive, escalating or lethality-relevant abuse. The review relies on published and retrievable records. Fourteen reports could not be retrieved despite legal and institutional access attempts and are listed in Supplementary Table S5 using metadata-level information only. Because these full texts were unavailable, their detailed PCC, methodological, validation and implementation characteristics could not be assessed; their non-retrieval may have introduced retrieval bias if unavailable reports differed systematically from retrieved records in publication type, technical detail, local implementation context or reporting completeness. Complementary legal and policy sources helped contextualise governance but do not constitute empirical evidence of AI effectiveness. Finally, the conceptual synthesis has not been validated as an intervention, risk-assessment tool or operational protocol. It should be treated as an interpretive structure for future research and policy discussion. Terms such as prediction, risk assessment, risk stratification and prevention were used inconsistently across the literature, so the synthesis imposed conceptual distinctions that improve interpretability but may not always match the terminology used by the original authors. Not every included study is discussed individually in the main text because the review synthesises a heterogeneous scoping corpus through representative clusters; all 125 records remain traceable in Supplementary Tables S1, S2.

## 7 Conclusion

AI-related methods are increasingly being studied across IPV and domestic-violence risk contexts, but the evidence does not support the prediction of individual femicide events or demonstrate that AI prevents lethal violence. The more defensible contribution lies in bounded support for recognition, linkage, documentation and escalation of risk signals within human-led institutional pathways. Such support is valuable only when it is connected to survivor-centred practice, professional judgment, multi-agency response, legal safeguards and accountable documentation. A conceptual synthesis of these pathways can help clarify where AI may assist and where medico-legal accountability must remain with institutions and professionals. In femicide-prevention contexts, a signal without a lawful, proportionate and resourced response is not prevention.

## Data Availability

The original contributions presented in the study are included in the article/**Supplementary Material**, further inquiries can be directed to the corresponding author.
