# Supplementary material for: Artificial intelligence in intimate partner violence risk pathways: a PRISMA-ScR review of femicide prevention and medico-legal accountability
Source: Front Digit Health. 2026 Jun 23;8:1877651. doi: 10.3389/fdgth.2026.1877651 (PMC13337629; doi:10.3389/fdgth.2026.1877651)
Supplement: Supplementary file 1 [file Table1.docx]

**Supplementary Materials**

Artificial Intelligence in Intimate Partner Violence Risk Pathways: A PRISMA-ScR Review of Femicide Prevention and Medico-Legal Accountability

Supplementary Tables 1-5

# Supplementary Materials overview

This supplementary file provides supporting materials for the PRISMA-ScR evidence map and conceptual synthesis. It includes the list of included empirical records, a record-level Population-Concept-Context (PCC) classification and data-charting matrix, the grey/legal-policy and institutional source audit, the database-specific search strategies, and the list of reports sought for retrieval but not retrieved.

# Supplementary Table 1. Included PRISMA-ScR core records

This table lists the 125 records included in the PRISMA-ScR core evidence map. Internal screening/export identifiers and filename-matching fields were removed. The numbering is provided only for readability and is not an internal screening code.

| **No.** | **Author/year** | **Title** | **Source/journal** | **Country/region** | **Study/design type** | **IPV/femicide pathway relevance** | **AI/digital method** | **Main relevance to the review** |
| --- | --- | --- | --- | --- | --- | --- | --- | --- |
| 1 | Garcia-Vergara et al. (2023) | Artificial intelligence extracts key insights from legal documents to predict intimate partner femicide. | Scientific reports; Sci Rep | Spain | model development/validation | femicide/feminicide; lethality/severe escalation | artificial intelligence; machine learning; NLP/text mining; predictive analytics/modeling | Links femicide/feminicide; lethality/severe escalation with detection/identification; prediction/forecasting; prevention/intervention/referral through artificial intelligence... |
| 2 | Xue et al. (2020) | The Hidden Pandemic of Family Violence During COVID-19: Unsupervised Learning of Tweets. | Journal of medical Internet research; J Med Internet Res | United States; Canada; China | model development/validation | intimate partner violence; domestic violence/abuse; family violence; coercive control... | machine learning; NLP/text mining; geospatial/search/speech/computer vision | Links intimate partner violence; domestic violence/abuse; family violence; coercive control; gender-based violence/violence against women with detection/identification; risk... |
| 3 | Verrey et al. (2023) | Using machine learning to forecast domestic homicide via police data and super learning. | Scientific reports; Sci Rep | United Kingdom; Israel | model development/validation | domestic violence/abuse; intimate partner homicide/domestic homicide; lethality/severe escalation | artificial intelligence; machine learning; predictive analytics/modeling; decision support/risk stratification | Links domestic violence/abuse; intimate partner homicide/domestic homicide; lethality/severe escalation with detection/identification; risk assessment/stratification... |
| 4 | da Silva B.B.A. et al. (2026) | Application of Machine Learning in the Detection and Prevention of Femicide: A Systematic Review | Lecture Notes in Networks and Systems | United States; Spain; Brazil | review | femicide/feminicide; gender-based violence/violence against women | machine learning; deep learning; predictive analytics/modeling | Links femicide/feminicide; gender-based violence/violence against women with detection/identification; prediction/forecasting; prevention/intervention/referral through machine... |
| 5 | Peddireddy et al. (2024) | Intimate Partner Homicide Among Women of Childbearing Age: Identifying Multilevel Risk Factors with Machine Learning. | AMIA ... Annual Symposium proceedings. AMIA Symposium; AMIA Annu Symp Proc | United States | model development/validation | intimate partner violence; intimate partner homicide/domestic homicide | machine learning | Links intimate partner violence; intimate partner homicide/domestic homicide with detection/identification; risk assessment/stratification; prediction/forecasting... |
| 6 | Trias Capella M.E. et al. (2024) | Treatment of the information on gender-based violence. With contributions from artificial intelligence; [Tratamiento de la informacion de violencia de género. Con aportaciones de la inteligencia artificial] | Revista Espanola de Medicina Legal | Spain | not reported/unclear | femicide/feminicide; gender-based violence/violence against women | artificial intelligence | Links femicide/feminicide; gender-based violence/violence against women with detection/identification; prevention/intervention/referral through artificial intelligence. |
| 7 | Hui et al. (2023) | Examining the Supports and Advice That Women With Intimate Partner Violence Experience Received in Online Health Communities: Text Mining Approach. | Journal of medical Internet research; J Med Internet Res | United States; China | model development/validation | intimate partner violence | NLP/text mining; geospatial/search/speech/computer vision | Links intimate partner violence with detection/identification; prediction/forecasting; prevention/intervention/referral through NLP/text mining; geospatial/search/speech/computer... |
| 8 | Harris et al. (2026) | Addressing diagnostic code variability in intimate partner violence surveillance through natural language processing: Evidence from substance use disorder populations. | Drug and alcohol dependence; Drug Alcohol Depend | United States | model development/validation | intimate partner violence | artificial intelligence; NLP/text mining; data mining; EHR/electronic health records | Links intimate partner violence with detection/identification; screening; risk assessment/stratification; early warning/surveillance; prevention/intervention/referral... |
| 9 | More S. et al. (2023) | A Proposal of Data Mining Model for the Classification of an Act of Violence as a Case of Attempted Femicide in the Peruvian Scope | Lecture Notes in Networks and Systems | India; Peru | model development/validation | intimate partner violence; femicide/feminicide; gender-based violence/violence against women | machine learning; predictive analytics/modeling; data mining | Links intimate partner violence; femicide/feminicide; gender-based violence/violence against women with detection/identification; prediction/forecasting... |
| 10 | Karystianis et al. (2022) | Surveillance of Domestic Violence Using Text Mining Outputs From Australian Police Records. | Frontiers in psychiatry; Front Psychiatry | United States; Australia; United Kingdom | not reported/unclear | domestic violence/abuse | NLP/text mining | Links domestic violence/abuse with detection/identification; early warning/surveillance; prevention/intervention/referral; legal/ethical governance through NLP/text mining. |
| 11 | Karystianis et al. (2022) | Mental Illness Concordance Between Hospital Clinical Records and Mentions in Domestic Violence Police Narratives: Data Linkage Study. | JMIR formative research; JMIR Form Res | Australia; United Kingdom; South Wales | observational/data-linkage study | domestic violence/abuse | NLP/text mining; record/data linkage | Links domestic violence/abuse with detection/identification; risk assessment/stratification; early warning/surveillance; inter-agency coordination; legal/ethical governance... |
| 12 | Randell et al. (2022) | Intimate Partner Violence and the Pediatric Electronic Health Record: A Qualitative Study. | Academic pediatrics; Acad Pediatr | not reported | qualitative study | intimate partner violence; stalking | EHR/electronic health records | Links intimate partner violence; stalking with detection/identification through EHR/electronic health records. |
| 13 | Gu et al. (2026) | Leveraging multimodal machine learning for accurate risk identification of intimate partner violence. | npj women's health; NPJ Womens Health | United States | model development/validation | intimate partner violence; domestic violence/abuse | machine learning; deep learning; NLP/text mining; LLM/generative AI; predictive analytics/modeling; decision... | Links intimate partner violence; domestic violence/abuse with detection/identification; screening; prediction/forecasting; decision support/triage... |
| 14 | Lenert et al. (2024) | Electronic Health Record-Based Screening for Intimate Partner Violence: A Cluster Randomized Clinical Trial. | JAMA network open; JAMA Netw Open | not reported | clinical trial / implementation trial | intimate partner violence | decision support/risk stratification; EHR/electronic health records | Links intimate partner violence with detection/identification; screening; risk assessment/stratification; early warning/surveillance; decision support/triage... |
| 15 | Karystianis G. et al. (2024) | Text mining domestic violence police narratives to identify behaviours linked to coercive control | Crime Science | United States; Australia; United Kingdom | not reported/unclear | domestic violence/abuse; family violence; coercive control | NLP/text mining | Links domestic violence/abuse; family violence; coercive control with detection/identification through NLP/text mining. |
| 16 | Syed et al. (2021) | Predictive value of indicators for identifying child maltreatment and intimate partner violence in coded electronic health records: a systematic review and meta-analysis. | Archives of disease in childhood; Arch Dis Child | United Kingdom | review | intimate partner violence; family violence | deep learning; EHR/electronic health records | Links intimate partner violence; family violence with detection/identification; prediction/forecasting; early warning/surveillance; prevention/intervention/referral... |
| 17 | Adily et al. (2021) | Text mining police narratives to identify types of abuse and victim injuries in family and domestic violence events | TRENDS AND ISSUES IN CRIME AND CRIMINAL JUSTICE | not reported | not reported/unclear | domestic violence/abuse; family violence | artificial intelligence; NLP/text mining | Links domestic violence/abuse; family violence with detection/identification; prediction/forecasting through artificial intelligence; NLP/text mining. |
| 18 | Karystianis et al. (2021) | Utilizing Text Mining, Data Linkage and Deep Learning in Police and Health Records to Predict Future Offenses in Family and Domestic Violence. | Frontiers in digital health; Front Digit Health | United States; Australia; United Kingdom | model development/validation | intimate partner violence; domestic violence/abuse; family violence | deep learning; NLP/text mining; predictive analytics/modeling; record/data linkage | Links intimate partner violence; domestic violence/abuse; family violence with detection/identification; risk assessment/stratification; prediction/forecasting through deep... |
| 19 | Bhargava et al. (2011) | A predictive model to help identify intimate partner violence based on diagnoses and phone calls. | American journal of preventive medicine; Am J Prev Med | not reported | model development/validation | intimate partner violence | predictive analytics/modeling; EHR/electronic health records | Links intimate partner violence with detection/identification; prediction/forecasting; prevention/intervention/referral; legal/ethical governance through predictive... |
| 20 | Tabaie et al. (2024) | Identifying Latent Patterns of Intimate Partner Violence Using Electronic Health Records and Association Rule Mining. | AMIA ... Annual Symposium proceedings. AMIA Symposium; AMIA Annu Symp Proc | United States | not reported/unclear | intimate partner violence | data mining; EHR/electronic health records | Links intimate partner violence with detection/identification; screening through data mining; EHR/electronic health records. |
| 21 | Hui et al. (2023) | Harnessing Machine Learning in Tackling Domestic Violence-An Integrative Review. | International journal of environmental research and public health; Int J Environ Res Public Health | United States; Australia | review | intimate partner violence; domestic violence/abuse | machine learning; NLP/text mining; EHR/electronic health records; geospatial/search/speech/computer vision | Links intimate partner violence; domestic violence/abuse with detection/identification; prediction/forecasting through machine learning; NLP/text mining; EHR/electronic health... |
| 22 | Yılmaz et al. (2023) | Adverse health correlates of intimate partner violence against older women: Mining electronic health records. | PLoS ONE | United States; Turkey; Kenya | not reported/unclear | intimate partner violence | EHR/electronic health records | Links intimate partner violence with detection/identification; screening through EHR/electronic health records. |
| 23 | Johnson et al. (2023) | Risk Factors Associated With Primary Care-Reported Domestic Violence for Women Involved in Family Law Care Proceedings: Data Linkage Observational Study. | Journal of medical Internet research; J Med Internet Res | United Kingdom | review | domestic violence/abuse | record/data linkage; EHR/electronic health records | Links domestic violence/abuse with detection/identification; risk assessment/stratification; prevention/intervention/referral through record/data linkage; EHR/electronic health... |
| 24 | Whitten et al. (2022) | Early developmental vulnerabilities following exposure to domestic violence and abuse: Findings from an Australian population cohort record linkage study. | Journal of psychiatric research; J Psychiatr Res | Australia; South Wales | observational/data-linkage study | domestic violence/abuse | record/data linkage | Links domestic violence/abuse with prevention/intervention/referral through record/data linkage. |
| 25 | Adily et al. (2021) | Text mining police narratives for mentions of mental disorders in family and domestic violence events | TRENDS AND ISSUES IN CRIME AND CRIMINAL JUSTICE | Australia | review | intimate partner violence; domestic violence/abuse; family violence; gender-based... | NLP/text mining | Links intimate partner violence; domestic violence/abuse; family violence; gender-based violence/violence against women; stalking with detection/identification through NLP/text... |
| 26 | Liu et al. (2020) | Interplay between traumatic brain injury and intimate partner violence: data driven analysis utilizing electronic health records. | BMC women's health; BMC Womens Health | United States | observational/data-linkage study | intimate partner violence; domestic violence/abuse | data mining; EHR/electronic health records | Links intimate partner violence; domestic violence/abuse with detection/identification; screening through data mining; EHR/electronic health records. |
| 27 | Karystianis et al. (2020) | Prevalence of Mental Illnesses in Domestic Violence Police Records: Text Mining Study. | Journal of medical Internet research; J Med Internet Res | United States; Australia; United Kingdom | not reported/unclear | domestic violence/abuse | NLP/text mining | Links domestic violence/abuse with detection/identification; prevention/intervention/referral through NLP/text mining. |
| 28 | Karystianis et al. (2019) | Automated Analysis of Domestic Violence Police Reports to Explore Abuse Types and Victim Injuries: Text Mining Study. | Journal of medical Internet research; J Med Internet Res | Australia; United Kingdom; South Wales | not reported/unclear | domestic violence/abuse; gender-based violence/violence against women | NLP/text mining | Links domestic violence/abuse; gender-based violence/violence against women with detection/identification through NLP/text mining. |
| 29 | Karakurt et al. (2017) | Mining Electronic Health Records Data: Domestic Violence and Adverse Health Effects. | Journal of family violence; J Fam Violence | United States | not reported/unclear | intimate partner violence; domestic violence/abuse | data mining; EHR/electronic health records | Links intimate partner violence; domestic violence/abuse with detection/identification; prevention/intervention/referral through data mining; EHR/electronic health records. |
| 30 | Kasa et al. (2024) | Exploring the Impact of Race on Addressing Intimate Partner Violence in the Emergency Department. | Pediatric emergency care; Pediatr Emerg Care | not reported | qualitative study | intimate partner violence; domestic violence/abuse | EHR/electronic health records | Links intimate partner violence; domestic violence/abuse with detection/identification; screening; decision support/triage; prevention/intervention/referral; legal/ethical... |
| 31 | Dogan et al. (2022) | Participatory Machine Learning Models in Feminicide News Alert Detection | THIRTY-SIXTH AAAI CONFERENCE ON ARTIFICIAL INTELLIGENCE / THIRTY-FOURTH CONFERENCE ON INNOVATIVE... | United States | qualitative study | femicide/feminicide; gender-based violence/violence against women; lethality/severe escalation | artificial intelligence; machine learning; NLP/text mining | Links femicide/feminicide; gender-based violence/violence against women; lethality/severe escalation with detection/identification; prediction/forecasting; early... |
| 32 | Prakash et al. (2024) | Antepartum Intimate Partner Violence: Development of a Risk Prediction Model. | Journal of women's health (2002); J Womens Health (Larchmt) | United States | qualitative study | intimate partner violence; domestic violence/abuse | deep learning; predictive analytics/modeling; decision support/risk stratification; EHR/electronic health... | Links intimate partner violence; domestic violence/abuse with detection/identification; screening; risk assessment/stratification; prediction/forecasting through deep learning... |
| 33 | Li et al. (2026) | The Role of Artificial Intelligence for Intimate Partner Violence Prevention: A Systematic Review. | Journal of clinical nursing; J Clin Nurs | United States | review | intimate partner violence | artificial intelligence; machine learning; deep learning; NLP/text mining; LLM/generative AI; predictive... | Links intimate partner violence with detection/identification; screening; risk assessment/stratification; prediction/forecasting; prevention/intervention/referral; legal/ethical... |
| 34 | D. D. Awasekar et al. (2025) | NyayaPhala (ProtectJC 2.0): Building India's First Statute-Aligned Legal Corpus for Training LAMP2 2.0 Machine Learning Models to Predict Reliefs in Domestic Violence Cases | 2025 IEEE 4th International Conference for Advancement in Technology, ICONAT 2025 | India | model development/validation | domestic violence/abuse | artificial intelligence; machine learning; predictive analytics/modeling; decision support/risk stratification | Links domestic violence/abuse with prediction/forecasting; decision support/triage; prevention/intervention/referral through artificial intelligence; machine learning; predictive... |
| 35 | Guan et al. (2025) | Classifying the Information Needs of Survivors of Domestic Violence in Online Health Communities Using Large Language Models: Prediction Model Development and Evaluation Study. | Journal of medical Internet research; J Med Internet Res | United States; China | model development/validation | intimate partner violence; domestic violence/abuse | artificial intelligence; NLP/text mining; LLM/generative AI; predictive analytics/modeling | Links intimate partner violence; domestic violence/abuse with detection/identification; prediction/forecasting; prevention/intervention/referral through artificial intelligence... |
| 36 | Kaikhosrovi et al. (2025) | State-wide analysis of trends and patterns of emergency department presentations of violence against women in New South Wales, Australia 2015-2022: a data linkage study. | BMJ Open | Australia; South Wales | observational/data-linkage study | intimate partner violence; domestic violence/abuse; family violence; gender-based... | artificial intelligence; data mining; record/data linkage | Links intimate partner violence; domestic violence/abuse; family violence; gender-based violence/violence against women with detection/identification; screening... |
| 37 | Özsezer et al. (2026) | The Dark Side of Love: Prediction of Digital Intimate Partner Violence and Associated Factors Among University Students Using Machine Learning. | Journal of interpersonal violence; J Interpers Violence | Turkey | model development/validation | intimate partner violence; domestic violence/abuse | machine learning; predictive analytics/modeling; geospatial/search/speech/computer vision | Links intimate partner violence; domestic violence/abuse with detection/identification; risk assessment/stratification; prediction/forecasting; early warning/surveillance... |
| 38 | F. Mousavi et al. (2025) | Real-Time Detection of Domestic Violence Indicators on Twitter Using NLP and Deep Learning | 2025 12th International Conference on Reliability, Infocom Technologies and Optimization ,Trends... | United Kingdom | model development/validation | intimate partner violence; domestic violence/abuse; gender-based violence/violence against women | artificial intelligence; machine learning; deep learning; NLP/text mining; predictive analytics/modeling... | Links intimate partner violence; domestic violence/abuse; gender-based violence/violence against women with detection/identification; prediction/forecasting... |
| 39 | Saudagar S. et al. (2025) | Towards Safer Homes: AI-Driven Predictions for Domestic Violence Prevention | Communications in Computer and Information Science | India; Bangladesh; Liberia | model development/validation | intimate partner violence; domestic violence/abuse; family violence; gender-based... | artificial intelligence; machine learning; deep learning; NLP/text mining; predictive analytics/modeling | Links intimate partner violence; domestic violence/abuse; family violence; gender-based violence/violence against women with detection/identification; risk... |
| 40 | A. F. A. H. Alnuaimi et al. (2025) | An Empirical Ensemble Machine Learning Approach for the Classification and Prediction of Domestic Violence Crimes in Iraq | 2025 3rd International Conference on Business Analytics for Technology and Security (ICBATS) | Iraq | model development/validation | domestic violence/abuse | machine learning; predictive analytics/modeling; data mining | Links domestic violence/abuse with detection/identification; prediction/forecasting; prevention/intervention/referral through machine learning; predictive analytics/modeling... |
| 41 | Cruz-Mendoza M.C. et al. (2025) | Machine Learning Applied to Improve Prevention of, Response to, and Understanding of Violence Against Women | INFORMATICS-BASEL | United States; India; Mexico | model development/validation | intimate partner violence; intimate partner homicide/domestic homicide; gender-based... | machine learning; deep learning; geospatial/search/speech/computer vision | Links intimate partner violence; intimate partner homicide/domestic homicide; gender-based violence/violence against women with detection/identification; prediction/forecasting... |
| 42 | Warnars H.L.H.S. et al. (2025) | Prediction of Violence Against Women Using Ensemble Learning Models: A Comparative Study of LightGBM, XGBoost, and Others | International Journal of Safety and Security Engineering | United Kingdom | model development/validation | domestic violence/abuse; gender-based violence/violence against women | artificial intelligence; machine learning; predictive analytics/modeling | Links domestic violence/abuse; gender-based violence/violence against women with detection/identification; prediction/forecasting; prevention/intervention/referral through... |
| 43 | González-Sanabria J.-S. et al. (2025) | XGBoost Classifier-Based Model to Predict the Nature of Gender-Based Violence. Case Study: Santander, Colombia | Journal of Universal Computer Science | Spain; Colombia | model development/validation | femicide/feminicide; gender-based violence/violence against women | machine learning | Links femicide/feminicide; gender-based violence/violence against women with detection/identification; prediction/forecasting; early warning/surveillance... |
| 44 | Shashidhara et al. (2024) | Using Machine Learning Prediction to Create a 15-question IPV Measurement Tool. | Journal of interpersonal violence; J Interpers Violence | United States; Spain; Australia | review | intimate partner violence; domestic violence/abuse | machine learning; predictive analytics/modeling | Links intimate partner violence; domestic violence/abuse with detection/identification; risk assessment/stratification; prediction/forecasting; legal/ethical governance through... |
| 45 | Aguilar et al. (2023) | A Comparative Study of Three Pre-trained Convolutional Neural Networks in the Detection of Violence Against Women | CIENCIA ERGO-SUM | not reported | model development/validation | domestic violence/abuse; gender-based violence/violence against women | artificial intelligence; deep learning; geospatial/search/speech/computer vision | Links domestic violence/abuse; gender-based violence/violence against women with detection/identification; prevention/intervention/referral through artificial intelligence; deep... |
| 46 | Jung et al. (2022) | Does Stalking Behavior Improve Risk Prediction of Intimate Partner Violence? | Victims and Offenders | Canada | model development/validation | intimate partner violence; coercive control; intimate partner homicide/domestic homicide; stalking | predictive analytics/modeling | Links intimate partner violence; coercive control; intimate partner homicide/domestic homicide; stalking with detection/identification; risk assessment/stratification... |
| 47 | Ludwig (2022) | Using Machine Learning to Identify High Risk Domestic Violence Offenders in NYC, Final Summary Overview | not available in the provided files | not reported | clinical trial / implementation trial | intimate partner violence; domestic violence/abuse; gender-based violence/violence against women | machine learning; predictive analytics/modeling; record/data linkage | Links intimate partner violence; domestic violence/abuse; gender-based violence/violence against women with detection/identification; prediction/forecasting... |
| 48 | Hossain M.M. et al. (2021) | Prediction on domestic violence in bangladesh during the covid-19 outbreak using machine learning methods | Applied System Innovation | Bangladesh; Italy; China | model development/validation | domestic violence/abuse; family violence; gender-based violence/violence against women | machine learning | Links domestic violence/abuse; family violence; gender-based violence/violence against women with prediction/forecasting; prevention/intervention/referral through machine learning. |
| 49 | Choi et al. (2016) | A Predictive Model of Domestic Violence in Multicultural Families Focusing on Perpetrator. | Asian nursing research; Asian Nurs Res (Korean Soc Nurs Sci) | Korea; South Korea | model development/validation | domestic violence/abuse; family violence | predictive analytics/modeling | Links domestic violence/abuse; family violence with prediction/forecasting; prevention/intervention/referral through predictive analytics/modeling. |
| 50 | Halpern et al. (2006) | A predictive model to identify women with injuries related to intimate partner violence. | Journal of the American Dental Association (1939); J Am Dent Assoc | not reported | clinical trial / implementation trial | intimate partner violence | predictive analytics/modeling | Links intimate partner violence with detection/identification; screening; risk assessment/stratification; prediction/forecasting; prevention/intervention/referral through... |
| 51 | Sprecher et al. (2004) | A neural network model analysis to identify victims of intimate partner violence. | The American journal of emergency medicine; Am J Emerg Med | not reported | model development/validation | intimate partner violence; domestic violence/abuse | deep learning | Links intimate partner violence; domestic violence/abuse with detection/identification; screening through deep learning. |
| 52 | Barboza-Salerno et al. (2026) | Sex-based differences in injury patterns and hospitalization from emergency department narratives of intimate partner violence, United States, 2013-2024. | The American journal of emergency medicine; Am J Emerg Med | United States | review | intimate partner violence | NLP/text mining | Links intimate partner violence with detection/identification; screening; prediction/forecasting; early warning/surveillance; prevention/intervention/referral; legal/ethical... |
| 53 | Turner et al. (2022) | Predicting Domestic Abuse (Fairly) and Police Risk Assessment; [La predicción (equitativa) de la violencia doméstica y la evaluación policial de riesgo] | Psychosocial intervention; Psychosoc Interv | Spain; United Kingdom | model development/validation | intimate partner violence; domestic violence/abuse; stalking | machine learning; predictive analytics/modeling | Links intimate partner violence; domestic violence/abuse; stalking with detection/identification; risk assessment/stratification; prediction/forecasting... |
| 54 | Warnars H.L.H.S. et al. (2026) | The Effect of Feature Selection Based on CatBoost, LIME, SHAP, and Random Forest in Identifying the Risk of Violence Against Women | Studies in Computational Intelligence | United Kingdom | model development/validation | gender-based violence/violence against women | machine learning; predictive analytics/modeling; geospatial/search/speech/computer vision | Links gender-based violence/violence against women with detection/identification; risk assessment/stratification; prediction/forecasting; early warning/surveillance... |
| 55 | Başaran et al. (2025) | Determining domestic violence against women using machine learning methods: The case of Türkiye. | Journal of evaluation in clinical practice; J Eval Clin Pract | Turkey | model development/validation | intimate partner violence; domestic violence/abuse; gender-based violence/violence against women... | artificial intelligence; machine learning; predictive analytics/modeling | Links intimate partner violence; domestic violence/abuse; gender-based violence/violence against women; stalking with detection/identification; risk assessment/stratification... |
| 56 | Cook et al. (2025) | Improving police recorded crime data for domestic violence and abuse through natural language processing. | Frontiers in sociology; Front Sociol | United Kingdom | model development/validation | domestic violence/abuse | machine learning; deep learning; NLP/text mining | Links domestic violence/abuse with detection/identification; risk assessment/stratification; prediction/forecasting; prevention/intervention/referral through machine learning... |
| 57 | Kurniawan T.B. et al. (2025) | Detecting Gender-Based Violence Discourse Using Deep Learning: A CNN-LSTM Hybrid Model Approach | Journal of Applied Data Sciences | not reported | model development/validation | gender-based violence/violence against women | artificial intelligence; machine learning; deep learning; geospatial/search/speech/computer vision | Links gender-based violence/violence against women with detection/identification through artificial intelligence; machine learning; deep learning... |
| 58 | Alnuaimi A.F.A.H. et al. (2025) | Statistical Comparison of Some Machine Learning Techniques: A Case Study for Classifying Domestic Violence Crimes in Iraq | Iraqi Journal of Science | Iraq | model development/validation | domestic violence/abuse | machine learning; deep learning; predictive analytics/modeling | Links domestic violence/abuse with detection/identification; prediction/forecasting; prevention/intervention/referral through machine learning; deep learning; predictive... |
| 59 | Hadjimatheou K. et al. (2024) | Using unsupervised machine learning to find profiles of domestic abuse perpetrators | POLICING-A JOURNAL OF POLICY AND PRACTICE | United Kingdom | qualitative study | domestic violence/abuse | machine learning; geospatial/search/speech/computer vision | Links domestic violence/abuse with detection/identification; prevention/intervention/referral through machine learning; geospatial/search/speech/computer vision. |
| 60 | De Filippo et al. (2023) | Effects of digital chatbot on gender attitudes and exposure to intimate partner violence among young women in South Africa. | PLOS digital health; PLOS Digit Health | United States; United Kingdom; South Africa | clinical trial / implementation trial | intimate partner violence | chatbot/digital assistant | Links intimate partner violence with detection/identification; screening; prevention/intervention/referral through chatbot/digital assistant. |
| 61 | Poelmans J. et al. (2011) | Text mining with emergent self organizing maps and multi-dimensional scaling: A comparative study on domestic violence | Applied Soft Computing Journal | Netherlands | model development/validation | domestic violence/abuse | NLP/text mining; data mining | Links domestic violence/abuse with detection/identification; decision support/triage through NLP/text mining; data mining. |
| 62 | Thompson Lee et al. (2026) | Harnessing the Power of Machine Learning to Prevent Gender-Based Violence: Using Big Data Techniques to Enhance Research on Violence Against Women. | Violence Against Women | United States | qualitative study | domestic violence/abuse; gender-based violence/violence against women | artificial intelligence; machine learning; deep learning; data mining; geospatial/search/speech/computer... | Links domestic violence/abuse; gender-based violence/violence against women with detection/identification; prediction/forecasting; prevention/intervention/referral through... |
| 63 | Vogt et al. (2026) | From needs assessment to usability testing: evaluating the AinoAid™ chatbot for domestic violence support. | BMC women's health; BMC Womens Health | Spain | qualitative study | domestic violence/abuse; intimate partner homicide/domestic homicide; gender-based... | artificial intelligence; chatbot/digital assistant | Links domestic violence/abuse; intimate partner homicide/domestic homicide; gender-based violence/violence against women with detection/identification; risk... |
| 64 | Awasekar D.D. et al. (2025) | Empowering Women Through AI: A Comprehensive Chatbot for Domestic Violence Awareness and Legal Support in India | FUTUREPROOFING ENGINEERING EDUCATION FOR GLOBAL RESPONSIBILITY, ICL2024, VOL 4 | India | model development/validation | domestic violence/abuse | artificial intelligence; machine learning; NLP/text mining; chatbot/digital assistant | Links domestic violence/abuse with prevention/intervention/referral; legal/ethical governance through artificial intelligence; machine learning; NLP/text mining; chatbot/digital... |
| 65 | Ogğuztüzün et al. (2024) | Interpretable Machine Learning to Identify Risk Factors for Recidivism in Intimate Partner Violence. | AMIA ... Annual Symposium proceedings. AMIA Symposium; AMIA Annu Symp Proc | United States | qualitative study | intimate partner violence; domestic violence/abuse | machine learning; predictive analytics/modeling | Links intimate partner violence; domestic violence/abuse with detection/identification; risk assessment/stratification; prediction/forecasting; prevention/intervention/referral... |
| 66 | Escobar-Linero et al. (2023) | Using machine learning-based systems to help predict disengagement from the legal proceedings by women victims of intimate partner violence in Spain. | PLoS ONE | Spain; India | model development/validation | intimate partner violence; gender-based violence/violence against women | artificial intelligence; machine learning; predictive analytics/modeling | Links intimate partner violence; gender-based violence/violence against women with detection/identification; prediction/forecasting; prevention/intervention/referral through... |
| 67 | Berk R.A. et al. (2016) | Forecasting Domestic Violence: A Machine Learning Approach to Help Inform Arraignment Decisions | Journal of Empirical Legal Studies | not reported | clinical trial / implementation trial | domestic violence/abuse | machine learning; predictive analytics/modeling | Links domestic violence/abuse with prediction/forecasting; decision support/triage; prevention/intervention/referral through machine learning; predictive analytics/modeling. |
| 68 | Um et al. (2025) | Predicting Intimate Partner Violence Perpetration Among Young Adults Experiencing Homelessness in Seven U.S. Cities Using Interpretable Machine Learning. | Journal of interpersonal violence; J Interpers Violence | United States | model development/validation | intimate partner violence; stalking | machine learning; predictive analytics/modeling | Links intimate partner violence; stalking with detection/identification; prediction/forecasting; prevention/intervention/referral through machine learning; predictive... |
| 69 | Awasekar D. et al. (2025) | Artificial Intelligence for Legal Assistance: A Prescriptive Analytics Model Integrating Social Emotional Learning for Assisting Victims of Domestic Violence in India | Lecture Notes in Educational Technology | United States; India; Turkey | qualitative study | domestic violence/abuse | artificial intelligence; chatbot/digital assistant; geospatial/search/speech/computer vision | Links domestic violence/abuse with detection/identification; prevention/intervention/referral through artificial intelligence; chatbot/digital assistant... |
| 70 | Hui et al. (2026) | Perspectives and preferences of domestic violence survivors regarding digital platform and AI chatbot for help-seeking: A qualitative study. | PLOS ONE | United States | qualitative study | intimate partner violence; domestic violence/abuse; family violence | artificial intelligence; chatbot/digital assistant | Links intimate partner violence; domestic violence/abuse; family violence with detection/identification; prevention/intervention/referral; legal/ethical governance through... |
| 71 | Verma P. et al. (2026) | Predictive Modeling of Domestic Violence in India: A Machine Learning Perspective | Journal of Statistical Theory and Applications | India | model development/validation | intimate partner violence; domestic violence/abuse; gender-based violence/violence against women | machine learning; deep learning; predictive analytics/modeling | Links intimate partner violence; domestic violence/abuse; gender-based violence/violence against women with detection/identification; prediction/forecasting... |
| 72 | Hernandez-Zetina S. et al. (2026) | Developing a Predictive Model for Gender-Based Violence in Urban Areas Using Open Data | Geomatics | Spain; Mexico | model development/validation | gender-based violence/violence against women | machine learning; deep learning; predictive analytics/modeling; geospatial/search/speech/computer vision | Links gender-based violence/violence against women with detection/identification; prediction/forecasting; prevention/intervention/referral through machine learning; deep... |
| 73 | Giorgio A. (2026) | A Smart App for the Prevention of Gender-Based Violence Using Artificial Intelligence | Electronics (Switzerland) | Italy | not reported/unclear | femicide/feminicide; gender-based violence/violence against women | artificial intelligence; geospatial/search/speech/computer vision | Links femicide/feminicide; gender-based violence/violence against women with detection/identification; early warning/surveillance; prevention/intervention/referral through... |
| 74 | Hui et al. (2025) | Leveraging GraphRAG with Large Language Models to Identify Help-Seeking Information Among Domestic Violence Survivors from Qualitative Interviews. | Studies in health technology and informatics; Stud Health Technol Inform | United States | clinical trial / implementation trial | domestic violence/abuse | deep learning; NLP/text mining; LLM/generative AI | Links domestic violence/abuse with detection/identification; prevention/intervention/referral through deep learning; NLP/text mining; LLM/generative AI. |
| 75 | Zhang et al. (2025) | Probing Social Support and Self-Disclosure Within Chinese Online Domestic Violence Support Groups: Leveraging Multiple Machine Learning Approaches. | Journal of community psychology; J Community Psychol | China | model development/validation | domestic violence/abuse | machine learning; deep learning; NLP/text mining; geospatial/search/speech/computer vision | Links domestic violence/abuse with detection/identification; prevention/intervention/referral through machine learning; deep learning; NLP/text mining... |
| 76 | Talwalk et al. (2025) | Domestic Abuse Survivor Assistance Through Forecasting and Engagement with Artificial Intelligence Solutions | COMPUTATIONAL SCIENCE AND COMPUTATIONAL INTELLIGENCE, CSCI 2024, PT II | United States; Canada | model development/validation | intimate partner violence; domestic violence/abuse; coercive control | artificial intelligence; predictive analytics/modeling | Links intimate partner violence; domestic violence/abuse; coercive control with detection/identification; prediction/forecasting; prevention/intervention/referral; legal/ethical... |
| 77 | Gonzálvez-Gallego N. et al. (2024) | Do search queries predict violence against women? A forecasting model based on Google Trends | Journal of Forecasting | United States; Spain | model development/validation | intimate partner violence; domestic violence/abuse; femicide/feminicide; gender-based... | predictive analytics/modeling; geospatial/search/speech/computer vision | Links intimate partner violence; domestic violence/abuse; femicide/feminicide; gender-based violence/violence against women; stalking with detection/identification; risk... |
| 78 | Salehi et al. (2023) | Domestic violence risk prediction in Iran using a machine learning approach by analyzing Persian textual content in social media. | Heliyon | Iran | clinical trial / implementation trial | domestic violence/abuse; family violence | machine learning; NLP/text mining; predictive analytics/modeling; geospatial/search/speech/computer vision | Links domestic violence/abuse; family violence with detection/identification; screening; prediction/forecasting; prevention/intervention/referral; legal/ethical governance... |
| 79 | Sisodia A. et al. (2023) | A Machine Learning Approach to Predict Poor Mental Health of Intimate Partner Violence Survivors | 2023 5th International Conference on Advances in Computational Tools for Engineering Applications... | India; Canada | review | intimate partner violence; gender-based violence/violence against women | machine learning; predictive analytics/modeling; geospatial/search/speech/computer vision | Links intimate partner violence; gender-based violence/violence against women with detection/identification; prediction/forecasting through machine learning; predictive... |
| 80 | Reyner-Fuentes et al. (2023) | Prediction of the Gender-based Violence Victim Condition using Speech: What do Machine Learning Models rely on? | Proceedings of the Annual Conference of the International Speech Communication Association... | Spain | qualitative study | gender-based violence/violence against women | artificial intelligence; machine learning; deep learning; geospatial/search/speech/computer vision | Links gender-based violence/violence against women with detection/identification; prediction/forecasting; legal/ethical governance through artificial intelligence; machine... |
| 81 | L. R. Abdulkareem et al. (2022) | Using ANN to Predict Gender-Based Violence in Iraq: How AI and data mining technologies revolutionized social networks to make a safer world | ISMSIT 2022 - 6th International Symposium on Multidisciplinary Studies and Innovative... | Iraq | model development/validation | gender-based violence/violence against women | artificial intelligence; machine learning; deep learning; predictive analytics/modeling; data mining... | Links gender-based violence/violence against women with detection/identification; prediction/forecasting; prevention/intervention/referral through artificial intelligence... |
| 82 | Victor et al. (2021) | Automated identification of domestic violence in written child welfare records: Leveraging text mining and machine learning to enhance social work research and evaluation. | Journal of the Society for Social Work and Research | United States | model development/validation | domestic violence/abuse | machine learning; deep learning; NLP/text mining; predictive analytics/modeling; record/data linkage | Links domestic violence/abuse with detection/identification; risk assessment/stratification; prediction/forecasting; prevention/intervention/referral through machine learning... |
| 83 | Castorena C.M. et al. (2021) | Deep neural network for gender-based violence detection on twitter messages | Mathematics | Mexico | model development/validation | gender-based violence/violence against women | machine learning; deep learning; geospatial/search/speech/computer vision | Links gender-based violence/violence against women with detection/identification through machine learning; deep learning; geospatial/search/speech/computer vision. |
| 84 | Rodríguez-Rodríguez I. et al. (2020) | Modeling and forecasting gender-based violence through machine learning techniques | Applied Sciences (Switzerland) | Spain; Italy | model development/validation | intimate partner violence; domestic violence/abuse; gender-based violence/violence against women | machine learning; deep learning; predictive analytics/modeling | Links intimate partner violence; domestic violence/abuse; gender-based violence/violence against women with prediction/forecasting; prevention/intervention/referral through... |
| 85 | Subramani et al. (2019) | Deep Learning for Multi-Class Identification from Domestic Violence Online Posts | IEEE Access | Australia; China | model development/validation | domestic violence/abuse; gender-based violence/violence against women | deep learning; geospatial/search/speech/computer vision | Links domestic violence/abuse; gender-based violence/violence against women with detection/identification; prediction/forecasting; prevention/intervention/referral through deep... |
| 86 | S. Subramani et al. (2018) | Domestic violence crisis identification from facebook posts based on deep learning | IEEE Access | Australia | model development/validation | domestic violence/abuse | machine learning; deep learning; geospatial/search/speech/computer vision | Links domestic violence/abuse with detection/identification; prevention/intervention/referral through machine learning; deep learning; geospatial/search/speech/computer vision. |
| 87 | Petering R. et al. (2018) | Artificial Intelligence to Predict Intimate Partner Violence Perpetration | Artificial Intelligence and Social Work | United States | model development/validation | intimate partner violence; domestic violence/abuse | artificial intelligence; machine learning | Links intimate partner violence; domestic violence/abuse with detection/identification; prediction/forecasting; decision support/triage; prevention/intervention/referral through... |
| 88 | Van Hightower et al. (2000) | Predictive models of domestic violence and fear of intimate partners among migrant and seasonal farm worker women. | Journal of Family Violence | not reported | model development/validation | domestic violence/abuse; family violence | deep learning; predictive analytics/modeling | Links domestic violence/abuse; family violence with screening; prediction/forecasting; prevention/intervention/referral through deep learning; predictive analytics/modeling. |
| 89 | Marisetti M. et al. (2026) | Real Time Sentiment Analysis of Domestic Violence Tweets | Lecture Notes in Electrical Engineering | United States; Spain; Australia | model development/validation | intimate partner violence; domestic violence/abuse | artificial intelligence; machine learning; geospatial/search/speech/computer vision | Links intimate partner violence; domestic violence/abuse with detection/identification; prevention/intervention/referral; legal/ethical governance through artificial... |
| 90 | Le B.L. et al. (2026) | Beyond physical violence: A machine learning framework for predicting IPV victimisation using multidimensional predictors | European Journal of Criminology | United Kingdom | protocol | intimate partner violence; domestic violence/abuse; femicide/feminicide; lethality/severe escalation | machine learning; predictive analytics/modeling | Links intimate partner violence; domestic violence/abuse; femicide/feminicide; lethality/severe escalation with detection/identification; risk assessment/stratification... |
| 91 | Singh S. et al. (2026) | Machine Learning in Domestic Violence: Current Trends and Future Prospectives | Lecture Notes in Networks and Systems | United States; Brazil; Australia | model development/validation | domestic violence/abuse | artificial intelligence; machine learning; deep learning | Links domestic violence/abuse with prevention/intervention/referral through artificial intelligence; machine learning; deep learning. |
| 92 | Sood V. (2025) | Empowering Interventions: AI and Machine Learning Solutions for Predicting and Managing Domestic Violence | Lecture Notes in Networks and Systems | United States; Brazil; India | model development/validation | domestic violence/abuse | artificial intelligence; machine learning; deep learning; predictive analytics/modeling; decision... | Links domestic violence/abuse with prediction/forecasting; decision support/triage; prevention/intervention/referral through artificial intelligence; machine learning; deep... |
| 93 | Hui et al. (2024) | Predicting the Information Need for Domestic Violence Survivors Based on the Fine-Tuned Large Language Model. | Studies in health technology and informatics; Stud Health Technol Inform | United States | model development/validation | intimate partner violence; domestic violence/abuse | artificial intelligence; machine learning; NLP/text mining; LLM/generative AI; predictive analytics/modeling | Links intimate partner violence; domestic violence/abuse with detection/identification; prediction/forecasting; prevention/intervention/referral through artificial intelligence... |
| 94 | Sachdeva et al. (2024) | A Traumatic Brain Injury Prescreening Tool for Intimate Partner Violence Patients Using Initial Clinical Reports and Machine Learning. | AMIA Joint Summits on Translational Science proceedings. AMIA Joint Summits on Translational... | not reported | model development/validation | intimate partner violence; domestic violence/abuse | machine learning; EHR/electronic health records | Links intimate partner violence; domestic violence/abuse with detection/identification; screening through machine learning; EHR/electronic health records. |
| 95 | Szyfer Lipinsky et al. (2024) | Predicting psychopathology in Jewish ultra-orthodox IPV survivors: A machine learning approach. | Journal of Loss and Trauma | Israel | qualitative study | intimate partner violence | machine learning; predictive analytics/modeling | Links intimate partner violence with detection/identification; risk assessment/stratification; prediction/forecasting; prevention/intervention/referral through machine learning... |
| 96 | Contreras-Jiménez et al. (2024) | Mapping Gender-Based Violence: Integrating Machine Learning and Geospatial Analysis in Mexico City | Communications in Computer and Information Science | United States; Mexico; South Africa | model development/validation | gender-based violence/violence against women | machine learning; predictive analytics/modeling; data mining; geospatial/search/speech/computer vision | Links gender-based violence/violence against women with detection/identification; prediction/forecasting; prevention/intervention/referral through machine learning; predictive... |
| 97 | Rahman et al. (2023) | A comparative study of machine learning algorithms for predicting domestic violence vulnerability in Liberian women. | BMC women's health; BMC Womens Health | Bangladesh; Liberia | model development/validation | intimate partner violence; domestic violence/abuse; gender-based violence/violence against women | machine learning; predictive analytics/modeling | Links intimate partner violence; domestic violence/abuse; gender-based violence/violence against women with detection/identification; risk assessment/stratification... |
| 98 | Chen et al. (2023) | Using machine learning to estimate the incidence rate of intimate partner violence. | Scientific reports; Sci Rep | China | qualitative study | intimate partner violence; domestic violence/abuse | machine learning | Links intimate partner violence; domestic violence/abuse with prediction/forecasting; prevention/intervention/referral; legal/ethical governance through machine learning. |
| 99 | Todorovic et al. (2022) | Prevalence, increase and predictors of family violence during the COVID-19 pandemic, using modern machine learning approaches. | Frontiers in psychiatry; Front Psychiatry | United States; Netherlands | model development/validation | intimate partner violence; family violence | machine learning | Links intimate partner violence; family violence with detection/identification; prediction/forecasting through machine learning. |
| 100 | Kennedy et al. (2023) | Insights from linking police domestic abuse data and health data in South Wales, UK: a linked routine data analysis using decision tree classification. | The Lancet. Public health; Lancet Public Health | United Kingdom; South Wales | model development/validation | domestic violence/abuse; stalking | record/data linkage | Links domestic violence/abuse; stalking with detection/identification; risk assessment/stratification; prevention/intervention/referral; inter-agency coordination; legal/ethical... |
| 101 | Tabaie et al. (2022) | A Novel Technique to Identify Intimate Partner Violence in a Hospital Setting. | The western journal of emergency medicine; West J Emerg Med | not reported | model development/validation | intimate partner violence | deep learning; NLP/text mining; EHR/electronic health records | Links intimate partner violence with detection/identification; screening; prevention/intervention/referral through deep learning; NLP/text mining; EHR/electronic health records. |
| 102 | D. D. Awasekar et al. (2025) | AI-Driven Extraction and Verification of Semi-Categorized Legal Documents: The LAMP2 4.0 Retrieval-Augmented Generation Framework for Statute-Aligned Legal Relief Prediction | 2025 3rd DMIHER International Conference on Artificial Intelligence in Healthcare, Education and... | India | model development/validation | domestic violence/abuse | artificial intelligence; NLP/text mining; LLM/generative AI | Links domestic violence/abuse with detection/identification; prediction/forecasting; prevention/intervention/referral through artificial intelligence; NLP/text mining... |
| 103 | Botelle et al. (2022) | Can natural language processing models extract and classify instances of interpersonal violence in mental healthcare electronic records: an applied evaluative study. | BMJ Open | not reported | model development/validation | domestic violence/abuse | artificial intelligence; deep learning; NLP/text mining; data mining; EHR/electronic health records | Links domestic violence/abuse with detection/identification through artificial intelligence; deep learning; NLP/text mining; data mining; EHR/electronic health records. |
| 104 | González et al. (2021) | A sentiment analysis and unsupervised learning approach to digital violence a... | Proceedings - 2021 4th International Conference on Information and Computer T... | Mexico | model development/evaluation | family violence; homicide/lethality; violence against women | AI/algorithmic tool | Links family violence; homicide/lethality; violence against women with detection/identification through AI/algorithmic tool. |
| 105 | V. Ezhumalai et al. (2025) | Artificial Intelligence keeps women safe: Preventing online and domestic Viol... | 2025 International Conference on Artificial Intelligence and Data Engineering... | India | model development/evaluation | domestic violence/abuse; harassment | NLP; machine learning; predictive analytics/risk prediction | Links domestic violence/abuse; harassment with detection/identification; risk assessment/stratification; prediction; early warning; decision support/assistance; prevention... |
| 106 | S. Talwalkar et al. (2023) | Designing User-Centered Artificial Intelligence to Assist in Recovery from Do... | Proceedings - 2023 Congress in Computer Science, Computer Engineering, and Ap... | United States | model development/evaluation | IPV; domestic violence/abuse | machine learning; data mining | Links IPV; domestic violence/abuse with detection/identification; risk assessment/stratification; prediction; decision support/assistance; prevention through machine learning... |
| 107 | Fathima K.S. et al. (2023) | Domestic Violence Detection System Using Natural Language Processing | Proceedings - 2023 International Conference on Innovations in Engineering and... | India | survey / cross-sectional | domestic violence/abuse | NLP; machine learning | Links domestic violence/abuse with detection/identification; decision support/assistance; prevention through NLP; machine learning. |
| 108 | Ngũnjiri et al. (2023) | Utilizing User Preferences in Designing the AGILE (Accelerating Access to Gen... | International journal of environmental research and public health; Int J Envi... | Kenya; United States | conceptual/technical paper | IPV; homicide/lethality; GBV | NLP; chatbot/virtual assistant | Links IPV; homicide/lethality; GBV with risk assessment/stratification; decision support/assistance; prevention through NLP; chatbot/virtual assistant. |
| 109 | A. K. Tiwari et al. (2024) | Leveraging Artificial Intelligence to Address Domestic Violence Against Women... | Proceedings of the 2024 International Conference on Innovative Computing, Int... | India; United States | model development/evaluation | domestic violence/abuse; violence against women | NLP; machine learning; predictive analytics/risk prediction; chatbot/virtual assistant | Links domestic violence/abuse; violence against women with detection/identification; risk assessment/stratification; prediction; early warning; decision support/assistance... |
| 110 | P. P. Shifidi et al. (2023) | Machine Learning-Based Analytical Process for Predicting the Occurrence of Ge... | 2023 International Conference on Emerging Trends in Networks and Computer Com... | Namibia | case study / model development | IPV; femicide; GBV | machine learning | Links IPV; femicide; GBV with detection/identification; risk assessment/stratification; prediction; decision support/assistance; prevention through machine learning. |
| 111 | R. Roy et al. (2023) | Meta-Analysis of Artificial Intelligence Solution for Prevention of Violence ... | 2023 International Conference on IoT, Communication and Automation Technology... | India | review / meta-analysis | violence against women; harassment | machine learning; chatbot/virtual assistant; EHR-based identification | Links violence against women; harassment with detection/identification; risk assessment/stratification; prediction; prevention through machine learning; chatbot/virtual... |
| 112 | Soldevilla et al. (2021) | Natural language processing through BERT for identifying gender-based violenc... | 2021 IEEE INTERNATIONAL CONFERENCE ON INFORMATION COMMUNICATION AND SOFTWARE ... | Peru | survey / cross-sectional | domestic violence/abuse; GBV; violence against women; harassment | NLP; machine learning | Links domestic violence/abuse; GBV; violence against women; harassment with detection/identification; classification; prediction; decision support/assistance through NLP; machine... |
| 113 | V. Itham et al. (2024) | Real-Time Sentiment Analysis of Domestic Violence Tweets: Informing Intervent... | Proceedings of the 2024 13th International Conference on System Modeling and ... | India | model development/evaluation | IPV; domestic violence/abuse | NLP; deep learning; machine learning; text mining/topic modelling | Links IPV; domestic violence/abuse with detection/identification; classification; prevention through NLP; deep learning; machine learning; text mining/topic modelling. |
| 114 | Stephanie E.M.A. et al. (2024) | Study of violence against women and its characteristics through the applicati... | International Journal of Data Science and Analytics | Mexico; Spain | model development/evaluation | domestic violence/abuse; femicide; GBV; violence against women | NLP; deep learning; machine learning; text mining/topic modelling | Links domestic violence/abuse; femicide; GBV; violence against women with detection/identification; classification; decision support/assistance through NLP; deep learning... |
| 115 | Alzyout M. et al. (2021) | Sentiment Analysis of Arabic Tweets about Violence against Women using Machin... | 2021 12th International Conference on Information and Communication Systems, ... | Jordan; Arab world; United States | model development/evaluation | violence against women | deep learning; machine learning | Links violence against women with detection/identification; classification; decision support/assistance through deep learning; machine learning. |
| 116 | D. D. Awasekar et al. (2025) | SWATI AI : Advancing AI Models from Rule-Based Frameworks to NLP-Driven Presc... | 2025 1st International Conference on AIML-Applications for Engineering and Te... | India | model development/evaluation | domestic violence/abuse | LLM; NLP; machine learning; chatbot/virtual assistant | Links domestic violence/abuse with detection/identification; prediction; decision support/assistance through LLM; NLP; machine learning; chatbot/virtual assistant. |
| 117 | Castro et al. (2019) | User acceptance of predictive analytics on violence against women and childre... | 2019 IEEE 9th International Conference on System Engineering and Technology, ... | Philippines | survey / cross-sectional | violence against women | predictive analytics/risk prediction | Links violence against women with risk assessment/stratification; prediction; decision support/assistance through predictive analytics/risk prediction. |
| 118 | Yallico Arias T. et al. (2022) | Automatic Detection of Levels of Intimate Partner Violence Against Women with... | Communications in Computer and Information Science | Peru; United States | model development/evaluation | IPV; domestic violence/abuse; femicide; violence against women | NLP; deep learning; machine learning | Links IPV; domestic violence/abuse; femicide; violence against women with detection/identification; risk assessment/stratification; decision support/assistance; prevention... |
| 119 | Gutam B.G. et al. (2025) | A Machine Learning and Community-Driven Approach for Feminist Cyber Resistanc... | 2025 4th OPJU International Technology Conference on Smart Computing for Inno... | India; United States | model development/evaluation | GBV; stalking; harassment | machine learning | Links GBV; stalking; harassment with detection/identification; classification; decision support/assistance through machine learning. |
| 120 | Karystianis et al. (2018) | Automatic Extraction of Mental Health Disorders From Domestic Violence Police... | Journal of medical Internet research; J Med Internet Res | Australia; United States | conceptual/technical paper | domestic violence/abuse; violence against women | text mining/topic modelling | Links domestic violence/abuse; violence against women with detection/identification; risk assessment/stratification; decision support/assistance through text mining/topic... |
| 121 | O. M. Cumbicus-Pineda et al. (2021) | Data Mining to Determine the Causes of Gender-Based Violence against Women in... | 2021 IEEE Fifth Ecuador Technical Chapters Meeting (ETCM) | Ecuador; United States | model development/evaluation | domestic violence/abuse; GBV; violence against women | NLP; deep learning; machine learning; data mining | Links domestic violence/abuse; GBV; violence against women with detection/identification; classification; prediction; decision support/assistance through NLP; deep learning... |
| 122 | Ismail O. et al. (2025) | Digitalizing Prevention and Detection of Violence Against Women and Girls Thr... | AI-SI 2025 - IEEE International Conference on Artificial Intelligence for Sus... | not reported | survey / cross-sectional | domestic violence/abuse; GBV; violence against women; stalking; harassment | NLP; deep learning; machine learning; predictive analytics/risk prediction; chatbot/virtual assistant | Links domestic violence/abuse; GBV; violence against women; stalking; harassment with detection/identification; classification; risk assessment/stratification; prediction... |
| 123 | Vowels et al. (2026) | Large language models for psychosocial risk assessment: A multi-method evalua... | PLOS digital health; PLOS Digit Health | United States | model development/evaluation | IPV | LLM; chatbot/virtual assistant | Links IPV with detection/identification; risk assessment/stratification; decision support/assistance through LLM; chatbot/virtual assistant. |
| 124 | Dehingia et al. (2022) | Help seeking behavior by women experiencing intimate partner violence in indi... | PLoS ONE | India; United States | survey / cross-sectional | IPV; GBV | machine learning | Links IPV; GBV with detection/identification; screening; risk assessment/stratification; decision support/assistance; prevention through machine learning. |
| 125 | Salehi et al. (2024) | Classification of domestic violence Persian textual content in social media b... | Heliyon | Iran | review / meta-analysis | domestic violence/abuse; violence against women | text mining/topic modelling; EHR-based identification | Links domestic violence/abuse; violence against women with detection/identification; classification; decision support/assistance; prevention through text mining/topic modelling... |

# Supplementary Table 2. Record-level PCC classification and data-charting matrix

This table provides record-level Population-Concept-Context (PCC) classification and data-charting fields for the 125 records included in the PRISMA-ScR core evidence map. For readability, the matrix is split into Supplementary Table 2A (PCC classification) and Supplementary Table 2B (technical, implementation and governance charting). Categories are not mutually exclusive. Where relationship status, LGBTQ+/transgender relevance or violence subtype was not reported by the original record or could not be identified from the available charting information, it was coded as "not reported/unclear" rather than inferred.

Supplementary Table 2A. PCC classification fields

| **Supplementary record no.** | **Author/year** | **Title** | **PCC - Population** | **Relationship status, if reported** | **Women/girls focus** | **Any-person IPV exposure** | **LGBTQ+/transgender relevance, if reported** | **Violence type** | **PCC - Concept / AI method** | **PCC - Context / setting** |
| --- | --- | --- | --- | --- | --- | --- | --- | --- | --- | --- |
| 1 | Garcia-Vergara et al. (2023) | Artificial intelligence extracts key insights from legal documents to predict intimate partner femicide. | Persons/cases affected by: femicide/feminicide; lethality/severe escalation | intimate-partner relationship; specific status not reported/unclear | yes | yes - IPV/domestic-abuse pathway | not reported/unclear | femicide/feminicide; lethality/severe escalation | artificial intelligence; machine learning; NLP/text mining; predictive analytics/modeling | police/legal/medico-legal |
| 2 | Xue et al. (2020) | The Hidden Pandemic of Family Violence During COVID-19: Unsupervised Learning of Tweets. | Persons/cases affected by: intimate partner violence; domestic violence/abuse; family violence; coercive control... | intimate-partner relationship; specific status not reported/unclear | not reported/unclear | yes - IPV/domestic-abuse pathway | not reported/unclear | intimate partner violence; domestic violence/abuse; family violence; coercive control... | machine learning; NLP/text mining; geospatial/search/speech/computer vision | healthcare/digital health |
| 3 | Verrey et al. (2023) | Using machine learning to forecast domestic homicide via police data and super learning. | Persons/cases affected by: domestic violence/abuse; intimate partner homicide/domestic homicide; lethality/severe escalation | intimate-partner relationship; specific status not reported/unclear | not reported/unclear | yes - IPV/domestic-abuse pathway | not reported/unclear | domestic violence/abuse; intimate partner homicide/domestic homicide; lethality/severe escalation | artificial intelligence; machine learning; predictive analytics/modeling; decision support/risk stratification | police/legal/medico-legal |
| 4 | da Silva B.B.A. et al. (2026) | Application of Machine Learning in the Detection and Prevention of Femicide: A Systematic Review | Persons/cases affected by: femicide/feminicide; gender-based violence/violence against women | not reported/unclear | yes | yes - IPV/domestic-abuse pathway | not reported/unclear | femicide/feminicide; gender-based violence/violence against women | machine learning; deep learning; predictive analytics/modeling | police/legal/medico-legal; digital/online |
| 5 | Peddireddy et al. (2024) | Intimate Partner Homicide Among Women of Childbearing Age: Identifying Multilevel Risk Factors with Machine Learning. | Persons/cases affected by: intimate partner violence; intimate partner homicide/domestic homicide | intimate-partner relationship; specific status not reported/unclear | yes | yes - IPV/domestic-abuse pathway | not reported/unclear | intimate partner violence; intimate partner homicide/domestic homicide | machine learning | police/legal/medico-legal |
| 6 | Trias Capella M.E. et al. (2024) | Treatment of the information on gender-based violence. With contributions from artificial intelligence; [Tratamiento de la informacion de violencia de género. Con aportaciones de la inteligencia artificial] | Persons/cases affected by: femicide/feminicide; gender-based violence/violence against women | not reported/unclear | yes | yes - IPV/domestic-abuse pathway | not reported/unclear | femicide/feminicide; gender-based violence/violence against women | artificial intelligence | police/legal/medico-legal |
| 7 | Hui et al. (2023) | Examining the Supports and Advice That Women With Intimate Partner Violence Experience Received in Online Health Communities: Text Mining Approach. | Persons/cases affected by: intimate partner violence | intimate-partner relationship; specific status not reported/unclear | yes | yes - IPV/domestic-abuse pathway | not reported/unclear | intimate partner violence | NLP/text mining; geospatial/search/speech/computer vision | healthcare/digital health; digital/online |
| 8 | Harris et al. (2026) | Addressing diagnostic code variability in intimate partner violence surveillance through natural language processing: Evidence from substance use disorder populations. | Persons/cases affected by: intimate partner violence | intimate-partner relationship; specific status not reported/unclear | not reported/unclear | yes - IPV/domestic-abuse pathway | not reported/unclear | intimate partner violence | artificial intelligence; NLP/text mining; data mining; EHR/electronic health records | healthcare/digital health; population/public health |
| 9 | More S. et al. (2023) | A Proposal of Data Mining Model for the Classification of an Act of Violence as a Case of Attempted Femicide in the Peruvian Scope | Persons/cases affected by: intimate partner violence; femicide/feminicide; gender-based violence/violence against women | intimate-partner relationship; specific status not reported/unclear | yes | yes - IPV/domestic-abuse pathway | not reported/unclear | intimate partner violence; femicide/feminicide; gender-based violence/violence against women | machine learning; predictive analytics/modeling; data mining | police/legal/medico-legal |
| 10 | Karystianis et al. (2022) | Surveillance of Domestic Violence Using Text Mining Outputs From Australian Police Records. | Persons/cases affected by: domestic violence/abuse | domestic/family relationship context; specific intimate-partner status not reported/unclear | not reported/unclear | yes - IPV/domestic-abuse pathway | not reported/unclear | domestic violence/abuse | NLP/text mining | police/legal/medico-legal |
| 11 | Karystianis et al. (2022) | Mental Illness Concordance Between Hospital Clinical Records and Mentions in Domestic Violence Police Narratives: Data Linkage Study. | Persons/cases affected by: domestic violence/abuse | domestic/family relationship context; specific intimate-partner status not reported/unclear | not reported/unclear | yes - IPV/domestic-abuse pathway | not reported/unclear | domestic violence/abuse | NLP/text mining; record/data linkage | healthcare/digital health; police/legal/medico-legal; multi-agency/linked data |
| 12 | Randell et al. (2022) | Intimate Partner Violence and the Pediatric Electronic Health Record: A Qualitative Study. | Persons/cases affected by: intimate partner violence; stalking | intimate-partner relationship; specific status not reported/unclear | not reported/unclear | yes - IPV/domestic-abuse pathway | not reported/unclear | intimate partner violence; stalking | EHR/electronic health records | healthcare/digital health |
| 13 | Gu et al. (2026) | Leveraging multimodal machine learning for accurate risk identification of intimate partner violence. | Persons/cases affected by: intimate partner violence; domestic violence/abuse | intimate-partner relationship; specific status not reported/unclear | not reported/unclear | yes - IPV/domestic-abuse pathway | not reported/unclear | intimate partner violence; domestic violence/abuse | machine learning; deep learning; NLP/text mining; LLM/generative AI; predictive analytics/modeling; decision... | not reported/unclear |
| 14 | Lenert et al. (2024) | Electronic Health Record-Based Screening for Intimate Partner Violence: A Cluster Randomized Clinical Trial. | Persons/cases affected by: intimate partner violence | intimate-partner relationship; specific status not reported/unclear | not reported/unclear | yes - IPV/domestic-abuse pathway | not reported/unclear | intimate partner violence | decision support/risk stratification; EHR/electronic health records | healthcare/digital health |
| 15 | Karystianis G. et al. (2024) | Text mining domestic violence police narratives to identify behaviours linked to coercive control | Persons/cases affected by: domestic violence/abuse; family violence; coercive control | domestic/family relationship context; specific intimate-partner status not reported/unclear | not reported/unclear | yes - IPV/domestic-abuse pathway | not reported/unclear | domestic violence/abuse; family violence; coercive control | NLP/text mining | police/legal/medico-legal |
| 16 | Syed et al. (2021) | Predictive value of indicators for identifying child maltreatment and intimate partner violence in coded electronic health records: a systematic review and meta-analysis. | Persons/cases affected by: intimate partner violence; family violence | intimate-partner relationship; specific status not reported/unclear | not reported/unclear | yes - IPV/domestic-abuse pathway | not reported/unclear | intimate partner violence; family violence | deep learning; EHR/electronic health records | healthcare/digital health |
| 17 | Adily et al. (2021) | Text mining police narratives to identify types of abuse and victim injuries in family and domestic violence events | Persons/cases affected by: domestic violence/abuse; family violence | domestic/family relationship context; specific intimate-partner status not reported/unclear | not reported/unclear | yes - IPV/domestic-abuse pathway | not reported/unclear | domestic violence/abuse; family violence | artificial intelligence; NLP/text mining | police/legal/medico-legal |
| 18 | Karystianis et al. (2021) | Utilizing Text Mining, Data Linkage and Deep Learning in Police and Health Records to Predict Future Offenses in Family and Domestic Violence. | Persons/cases affected by: intimate partner violence; domestic violence/abuse; family violence | intimate-partner relationship; specific status not reported/unclear | not reported/unclear | yes - IPV/domestic-abuse pathway | not reported/unclear | intimate partner violence; domestic violence/abuse; family violence | deep learning; NLP/text mining; predictive analytics/modeling; record/data linkage | healthcare/digital health; police/legal/medico-legal; digital/online; multi-agency/linked data |
| 19 | Bhargava et al. (2011) | A predictive model to help identify intimate partner violence based on diagnoses and phone calls. | Persons/cases affected by: intimate partner violence | intimate-partner relationship; specific status not reported/unclear | not reported/unclear | yes - IPV/domestic-abuse pathway | not reported/unclear | intimate partner violence | predictive analytics/modeling; EHR/electronic health records | healthcare/digital health |
| 20 | Tabaie et al. (2024) | Identifying Latent Patterns of Intimate Partner Violence Using Electronic Health Records and Association Rule Mining. | Persons/cases affected by: intimate partner violence | intimate-partner relationship; specific status not reported/unclear | not reported/unclear | yes - IPV/domestic-abuse pathway | not reported/unclear | intimate partner violence | data mining; EHR/electronic health records | healthcare/digital health |
| 21 | Hui et al. (2023) | Harnessing Machine Learning in Tackling Domestic Violence-An Integrative Review. | Persons/cases affected by: intimate partner violence; domestic violence/abuse | intimate-partner relationship; specific status not reported/unclear | not reported/unclear | yes - IPV/domestic-abuse pathway | not reported/unclear | intimate partner violence; domestic violence/abuse | machine learning; NLP/text mining; EHR/electronic health records; geospatial/search/speech/computer vision | healthcare/digital health |
| 22 | Yılmaz et al. (2023) | Adverse health correlates of intimate partner violence against older women: Mining electronic health records. | Persons/cases affected by: intimate partner violence | intimate-partner relationship; specific status not reported/unclear | yes | yes - IPV/domestic-abuse pathway | not reported/unclear | intimate partner violence | EHR/electronic health records | healthcare/digital health |
| 23 | Johnson et al. (2023) | Risk Factors Associated With Primary Care-Reported Domestic Violence for Women Involved in Family Law Care Proceedings: Data Linkage Observational Study. | Persons/cases affected by: domestic violence/abuse | domestic/family relationship context; specific intimate-partner status not reported/unclear | yes | yes - IPV/domestic-abuse pathway | not reported/unclear | domestic violence/abuse | record/data linkage; EHR/electronic health records | healthcare/digital health; multi-agency/linked data |
| 24 | Whitten et al. (2022) | Early developmental vulnerabilities following exposure to domestic violence and abuse: Findings from an Australian population cohort record linkage study. | Persons/cases affected by: domestic violence/abuse | domestic/family relationship context; specific intimate-partner status not reported/unclear | not reported/unclear | yes - IPV/domestic-abuse pathway | not reported/unclear | domestic violence/abuse | record/data linkage | population/public health; multi-agency/linked data |
| 25 | Adily et al. (2021) | Text mining police narratives for mentions of mental disorders in family and domestic violence events | Persons/cases affected by: intimate partner violence; domestic violence/abuse; family violence; gender-based... | intimate-partner relationship; specific status not reported/unclear | not reported/unclear | yes - IPV/domestic-abuse pathway | not reported/unclear | intimate partner violence; domestic violence/abuse; family violence; gender-based... | NLP/text mining | police/legal/medico-legal |
| 26 | Liu et al. (2020) | Interplay between traumatic brain injury and intimate partner violence: data driven analysis utilizing electronic health records. | Persons/cases affected by: intimate partner violence; domestic violence/abuse | intimate-partner relationship; specific status not reported/unclear | not reported/unclear | yes - IPV/domestic-abuse pathway | not reported/unclear | intimate partner violence; domestic violence/abuse | data mining; EHR/electronic health records | healthcare/digital health |
| 27 | Karystianis et al. (2020) | Prevalence of Mental Illnesses in Domestic Violence Police Records: Text Mining Study. | Persons/cases affected by: domestic violence/abuse | domestic/family relationship context; specific intimate-partner status not reported/unclear | not reported/unclear | yes - IPV/domestic-abuse pathway | not reported/unclear | domestic violence/abuse | NLP/text mining | healthcare/digital health; police/legal/medico-legal; population/public health |
| 28 | Karystianis et al. (2019) | Automated Analysis of Domestic Violence Police Reports to Explore Abuse Types and Victim Injuries: Text Mining Study. | Persons/cases affected by: domestic violence/abuse; gender-based violence/violence against women | domestic/family relationship context; specific intimate-partner status not reported/unclear | yes | yes - IPV/domestic-abuse pathway | not reported/unclear | domestic violence/abuse; gender-based violence/violence against women | NLP/text mining | healthcare/digital health; police/legal/medico-legal |
| 29 | Karakurt et al. (2017) | Mining Electronic Health Records Data: Domestic Violence and Adverse Health Effects. | Persons/cases affected by: intimate partner violence; domestic violence/abuse | intimate-partner relationship; specific status not reported/unclear | not reported/unclear | yes - IPV/domestic-abuse pathway | not reported/unclear | intimate partner violence; domestic violence/abuse | data mining; EHR/electronic health records | healthcare/digital health |
| 30 | Kasa et al. (2024) | Exploring the Impact of Race on Addressing Intimate Partner Violence in the Emergency Department. | Persons/cases affected by: intimate partner violence; domestic violence/abuse | intimate-partner relationship; specific status not reported/unclear | not reported/unclear | yes - IPV/domestic-abuse pathway | not reported/unclear | intimate partner violence; domestic violence/abuse | EHR/electronic health records | healthcare/digital health |
| 31 | Dogan et al. (2022) | Participatory Machine Learning Models in Feminicide News Alert Detection | Persons/cases affected by: femicide/feminicide; gender-based violence/violence against women; lethality/severe escalation | not reported/unclear | yes | yes - IPV/domestic-abuse pathway | not reported/unclear | femicide/feminicide; gender-based violence/violence against women; lethality/severe escalation | artificial intelligence; machine learning; NLP/text mining | not reported/unclear |
| 32 | Prakash et al. (2024) | Antepartum Intimate Partner Violence: Development of a Risk Prediction Model. | Persons/cases affected by: intimate partner violence; domestic violence/abuse | intimate-partner relationship; specific status not reported/unclear | not reported/unclear | yes - IPV/domestic-abuse pathway | not reported/unclear | intimate partner violence; domestic violence/abuse | deep learning; predictive analytics/modeling; decision support/risk stratification; EHR/electronic health... | healthcare/digital health |
| 33 | Li et al. (2026) | The Role of Artificial Intelligence for Intimate Partner Violence Prevention: A Systematic Review. | Persons/cases affected by: intimate partner violence | intimate-partner relationship; specific status not reported/unclear | not reported/unclear | yes - IPV/domestic-abuse pathway | not reported/unclear | intimate partner violence | artificial intelligence; machine learning; deep learning; NLP/text mining; LLM/generative AI; predictive... | healthcare/digital health |
| 34 | D. D. Awasekar et al. (2025) | NyayaPhala (ProtectJC 2.0): Building India's First Statute-Aligned Legal Corpus for Training LAMP2 2.0 Machine Learning Models to Predict Reliefs in Domestic Violence Cases | Persons/cases affected by: domestic violence/abuse | domestic/family relationship context; specific intimate-partner status not reported/unclear | not reported/unclear | yes - IPV/domestic-abuse pathway | not reported/unclear | domestic violence/abuse | artificial intelligence; machine learning; predictive analytics/modeling; decision support/risk stratification | police/legal/medico-legal |
| 35 | Guan et al. (2025) | Classifying the Information Needs of Survivors of Domestic Violence in Online Health Communities Using Large Language Models: Prediction Model Development and Evaluation Study. | Persons/cases affected by: intimate partner violence; domestic violence/abuse | intimate-partner relationship; specific status not reported/unclear | not reported/unclear | yes - IPV/domestic-abuse pathway | not reported/unclear | intimate partner violence; domestic violence/abuse | artificial intelligence; NLP/text mining; LLM/generative AI; predictive analytics/modeling | healthcare/digital health; digital/online |
| 36 | Kaikhosrovi et al. (2025) | State-wide analysis of trends and patterns of emergency department presentations of violence against women in New South Wales, Australia 2015-2022: a data linkage study. | Persons/cases affected by: intimate partner violence; domestic violence/abuse; family violence; gender-based... | intimate-partner relationship; specific status not reported/unclear | yes | yes - IPV/domestic-abuse pathway | not reported/unclear | intimate partner violence; domestic violence/abuse; family violence; gender-based... | artificial intelligence; data mining; record/data linkage | healthcare/digital health; multi-agency/linked data |
| 37 | Özsezer et al. (2026) | The Dark Side of Love: Prediction of Digital Intimate Partner Violence and Associated Factors Among University Students Using Machine Learning. | Persons/cases affected by: intimate partner violence; domestic violence/abuse | intimate-partner relationship; specific status not reported/unclear | not reported/unclear | yes - IPV/domestic-abuse pathway | not reported/unclear | intimate partner violence; domestic violence/abuse | machine learning; predictive analytics/modeling; geospatial/search/speech/computer vision | digital/online |
| 38 | F. Mousavi et al. (2025) | Real-Time Detection of Domestic Violence Indicators on Twitter Using NLP and Deep Learning | Persons/cases affected by: intimate partner violence; domestic violence/abuse; gender-based violence/violence against women | intimate-partner relationship; specific status not reported/unclear | yes | yes - IPV/domestic-abuse pathway | not reported/unclear | intimate partner violence; domestic violence/abuse; gender-based violence/violence against women | artificial intelligence; machine learning; deep learning; NLP/text mining; predictive analytics/modeling... | digital/online |
| 39 | Saudagar S. et al. (2025) | Towards Safer Homes: AI-Driven Predictions for Domestic Violence Prevention | Persons/cases affected by: intimate partner violence; domestic violence/abuse; family violence; gender-based... | intimate-partner relationship; specific status not reported/unclear | not reported/unclear | yes - IPV/domestic-abuse pathway | not reported/unclear | intimate partner violence; domestic violence/abuse; family violence; gender-based... | artificial intelligence; machine learning; deep learning; NLP/text mining; predictive analytics/modeling | not reported/unclear |
| 40 | A. F. A. H. Alnuaimi et al. (2025) | An Empirical Ensemble Machine Learning Approach for the Classification and Prediction of Domestic Violence Crimes in Iraq | Persons/cases affected by: domestic violence/abuse | domestic/family relationship context; specific intimate-partner status not reported/unclear | not reported/unclear | yes - IPV/domestic-abuse pathway | not reported/unclear | domestic violence/abuse | machine learning; predictive analytics/modeling; data mining | police/legal/medico-legal; digital/online |
| 41 | Cruz-Mendoza M.C. et al. (2025) | Machine Learning Applied to Improve Prevention of, Response to, and Understanding of Violence Against Women | Persons/cases affected by: intimate partner violence; intimate partner homicide/domestic homicide; gender-based... | intimate-partner relationship; specific status not reported/unclear | yes | yes - IPV/domestic-abuse pathway | not reported/unclear | intimate partner violence; intimate partner homicide/domestic homicide; gender-based... | machine learning; deep learning; geospatial/search/speech/computer vision | digital/online |
| 42 | Warnars H.L.H.S. et al. (2025) | Prediction of Violence Against Women Using Ensemble Learning Models: A Comparative Study of LightGBM, XGBoost, and Others | Persons/cases affected by: domestic violence/abuse; gender-based violence/violence against women | domestic/family relationship context; specific intimate-partner status not reported/unclear | yes | yes - IPV/domestic-abuse pathway | not reported/unclear | domestic violence/abuse; gender-based violence/violence against women | artificial intelligence; machine learning; predictive analytics/modeling | not reported/unclear |
| 43 | González-Sanabria J.-S. et al. (2025) | XGBoost Classifier-Based Model to Predict the Nature of Gender-Based Violence. Case Study: Santander, Colombia | Persons/cases affected by: femicide/feminicide; gender-based violence/violence against women | not reported/unclear | yes | yes - IPV/domestic-abuse pathway | not reported/unclear | femicide/feminicide; gender-based violence/violence against women | machine learning | not reported/unclear |
| 44 | Shashidhara et al. (2024) | Using Machine Learning Prediction to Create a 15-question IPV Measurement Tool. | Persons/cases affected by: intimate partner violence; domestic violence/abuse | intimate-partner relationship; specific status not reported/unclear | not reported/unclear | yes - IPV/domestic-abuse pathway | not reported/unclear | intimate partner violence; domestic violence/abuse | machine learning; predictive analytics/modeling | not reported/unclear |
| 45 | Aguilar et al. (2023) | A Comparative Study of Three Pre-trained Convolutional Neural Networks in the Detection of Violence Against Women | Persons/cases affected by: domestic violence/abuse; gender-based violence/violence against women | domestic/family relationship context; specific intimate-partner status not reported/unclear | yes | yes - IPV/domestic-abuse pathway | not reported/unclear | domestic violence/abuse; gender-based violence/violence against women | artificial intelligence; deep learning; geospatial/search/speech/computer vision | not reported/unclear |
| 46 | Jung et al. (2022) | Does Stalking Behavior Improve Risk Prediction of Intimate Partner Violence? | Persons/cases affected by: intimate partner violence; coercive control; intimate partner homicide/domestic homicide; stalking | intimate-partner relationship; specific status not reported/unclear | not reported/unclear | yes - IPV/domestic-abuse pathway | not reported/unclear | intimate partner violence; coercive control; intimate partner homicide/domestic homicide; stalking | predictive analytics/modeling | not reported/unclear |
| 47 | Ludwig (2022) | Using Machine Learning to Identify High Risk Domestic Violence Offenders in NYC, Final Summary Overview | Persons/cases affected by: intimate partner violence; domestic violence/abuse; gender-based violence/violence against women | intimate-partner relationship; specific status not reported/unclear | yes | yes - IPV/domestic-abuse pathway | not reported/unclear | intimate partner violence; domestic violence/abuse; gender-based violence/violence against women | machine learning; predictive analytics/modeling; record/data linkage | multi-agency/linked data |
| 48 | Hossain M.M. et al. (2021) | Prediction on domestic violence in bangladesh during the covid-19 outbreak using machine learning methods | Persons/cases affected by: domestic violence/abuse; family violence; gender-based violence/violence against women | domestic/family relationship context; specific intimate-partner status not reported/unclear | yes | yes - IPV/domestic-abuse pathway | not reported/unclear | domestic violence/abuse; family violence; gender-based violence/violence against women | machine learning | digital/online |
| 49 | Choi et al. (2016) | A Predictive Model of Domestic Violence in Multicultural Families Focusing on Perpetrator. | Persons/cases affected by: domestic violence/abuse; family violence | domestic/family relationship context; specific intimate-partner status not reported/unclear | not reported/unclear | yes - IPV/domestic-abuse pathway | not reported/unclear | domestic violence/abuse; family violence | predictive analytics/modeling | not reported/unclear |
| 50 | Halpern et al. (2006) | A predictive model to identify women with injuries related to intimate partner violence. | Persons/cases affected by: intimate partner violence | intimate-partner relationship; specific status not reported/unclear | yes | yes - IPV/domestic-abuse pathway | not reported/unclear | intimate partner violence | predictive analytics/modeling | healthcare/digital health |
| 51 | Sprecher et al. (2004) | A neural network model analysis to identify victims of intimate partner violence. | Persons/cases affected by: intimate partner violence; domestic violence/abuse | intimate-partner relationship; specific status not reported/unclear | not reported/unclear | yes - IPV/domestic-abuse pathway | not reported/unclear | intimate partner violence; domestic violence/abuse | deep learning | healthcare/digital health |
| 52 | Barboza-Salerno et al. (2026) | Sex-based differences in injury patterns and hospitalization from emergency department narratives of intimate partner violence, United States, 2013-2024. | Persons/cases affected by: intimate partner violence | intimate-partner relationship; specific status not reported/unclear | not reported/unclear | yes - IPV/domestic-abuse pathway | not reported/unclear | intimate partner violence | NLP/text mining | healthcare/digital health |
| 53 | Turner et al. (2022) | Predicting Domestic Abuse (Fairly) and Police Risk Assessment; [La predicción (equitativa) de la violencia doméstica y la evaluación policial de riesgo] | Persons/cases affected by: intimate partner violence; domestic violence/abuse; stalking | intimate-partner relationship; specific status not reported/unclear | not reported/unclear | yes - IPV/domestic-abuse pathway | not reported/unclear | intimate partner violence; domestic violence/abuse; stalking | machine learning; predictive analytics/modeling | police/legal/medico-legal |
| 54 | Warnars H.L.H.S. et al. (2026) | The Effect of Feature Selection Based on CatBoost, LIME, SHAP, and Random Forest in Identifying the Risk of Violence Against Women | Persons/cases affected by: gender-based violence/violence against women | not reported/unclear | yes | not reported/unclear | not reported/unclear | gender-based violence/violence against women | machine learning; predictive analytics/modeling; geospatial/search/speech/computer vision | not reported/unclear |
| 55 | Başaran et al. (2025) | Determining domestic violence against women using machine learning methods: The case of Türkiye. | Persons/cases affected by: intimate partner violence; domestic violence/abuse; gender-based violence/violence against women... | intimate-partner relationship; specific status not reported/unclear | yes | yes - IPV/domestic-abuse pathway | not reported/unclear | intimate partner violence; domestic violence/abuse; gender-based violence/violence against women... | artificial intelligence; machine learning; predictive analytics/modeling | healthcare/digital health |
| 56 | Cook et al. (2025) | Improving police recorded crime data for domestic violence and abuse through natural language processing. | Persons/cases affected by: domestic violence/abuse | domestic/family relationship context; specific intimate-partner status not reported/unclear | not reported/unclear | yes - IPV/domestic-abuse pathway | not reported/unclear | domestic violence/abuse | machine learning; deep learning; NLP/text mining | police/legal/medico-legal |
| 57 | Kurniawan T.B. et al. (2025) | Detecting Gender-Based Violence Discourse Using Deep Learning: A CNN-LSTM Hybrid Model Approach | Persons/cases affected by: gender-based violence/violence against women | not reported/unclear | yes | not reported/unclear | not reported/unclear | gender-based violence/violence against women | artificial intelligence; machine learning; deep learning; geospatial/search/speech/computer vision | digital/online |
| 58 | Alnuaimi A.F.A.H. et al. (2025) | Statistical Comparison of Some Machine Learning Techniques: A Case Study for Classifying Domestic Violence Crimes in Iraq | Persons/cases affected by: domestic violence/abuse | domestic/family relationship context; specific intimate-partner status not reported/unclear | not reported/unclear | yes - IPV/domestic-abuse pathway | not reported/unclear | domestic violence/abuse | machine learning; deep learning; predictive analytics/modeling | police/legal/medico-legal |
| 59 | Hadjimatheou K. et al. (2024) | Using unsupervised machine learning to find profiles of domestic abuse perpetrators | Persons/cases affected by: domestic violence/abuse | domestic/family relationship context; specific intimate-partner status not reported/unclear | not reported/unclear | yes - IPV/domestic-abuse pathway | not reported/unclear | domestic violence/abuse | machine learning; geospatial/search/speech/computer vision | not reported/unclear |
| 60 | De Filippo et al. (2023) | Effects of digital chatbot on gender attitudes and exposure to intimate partner violence among young women in South Africa. | Persons/cases affected by: intimate partner violence | intimate-partner relationship; specific status not reported/unclear | yes | yes - IPV/domestic-abuse pathway | not reported/unclear | intimate partner violence | chatbot/digital assistant | digital/online |
| 61 | Poelmans J. et al. (2011) | Text mining with emergent self organizing maps and multi-dimensional scaling: A comparative study on domestic violence | Persons/cases affected by: domestic violence/abuse | domestic/family relationship context; specific intimate-partner status not reported/unclear | not reported/unclear | yes - IPV/domestic-abuse pathway | not reported/unclear | domestic violence/abuse | NLP/text mining; data mining | digital/online |
| 62 | Thompson Lee et al. (2026) | Harnessing the Power of Machine Learning to Prevent Gender-Based Violence: Using Big Data Techniques to Enhance Research on Violence Against Women. | Persons/cases affected by: domestic violence/abuse; gender-based violence/violence against women | domestic/family relationship context; specific intimate-partner status not reported/unclear | yes | yes - IPV/domestic-abuse pathway | not reported/unclear | domestic violence/abuse; gender-based violence/violence against women | artificial intelligence; machine learning; deep learning; data mining; geospatial/search/speech/computer... | not reported/unclear |
| 63 | Vogt et al. (2026) | From needs assessment to usability testing: evaluating the AinoAid™ chatbot for domestic violence support. | Persons/cases affected by: domestic violence/abuse; intimate partner homicide/domestic homicide; gender-based... | intimate-partner relationship; specific status not reported/unclear | not reported/unclear | yes - IPV/domestic-abuse pathway | not reported/unclear | domestic violence/abuse; intimate partner homicide/domestic homicide; gender-based... | artificial intelligence; chatbot/digital assistant | digital/online |
| 64 | Awasekar D.D. et al. (2025) | Empowering Women Through AI: A Comprehensive Chatbot for Domestic Violence Awareness and Legal Support in India | Persons/cases affected by: domestic violence/abuse | domestic/family relationship context; specific intimate-partner status not reported/unclear | yes | yes - IPV/domestic-abuse pathway | not reported/unclear | domestic violence/abuse | artificial intelligence; machine learning; NLP/text mining; chatbot/digital assistant | police/legal/medico-legal; digital/online |
| 65 | Ogğuztüzün et al. (2024) | Interpretable Machine Learning to Identify Risk Factors for Recidivism in Intimate Partner Violence. | Persons/cases affected by: intimate partner violence; domestic violence/abuse | intimate-partner relationship; specific status not reported/unclear | not reported/unclear | yes - IPV/domestic-abuse pathway | not reported/unclear | intimate partner violence; domestic violence/abuse | machine learning; predictive analytics/modeling | not reported/unclear |
| 66 | Escobar-Linero et al. (2023) | Using machine learning-based systems to help predict disengagement from the legal proceedings by women victims of intimate partner violence in Spain. | Persons/cases affected by: intimate partner violence; gender-based violence/violence against women | intimate-partner relationship; specific status not reported/unclear | yes | yes - IPV/domestic-abuse pathway | not reported/unclear | intimate partner violence; gender-based violence/violence against women | artificial intelligence; machine learning; predictive analytics/modeling | police/legal/medico-legal |
| 67 | Berk R.A. et al. (2016) | Forecasting Domestic Violence: A Machine Learning Approach to Help Inform Arraignment Decisions | Persons/cases affected by: domestic violence/abuse | domestic/family relationship context; specific intimate-partner status not reported/unclear | not reported/unclear | yes - IPV/domestic-abuse pathway | not reported/unclear | domestic violence/abuse | machine learning; predictive analytics/modeling | police/legal/medico-legal; digital/online |
| 68 | Um et al. (2025) | Predicting Intimate Partner Violence Perpetration Among Young Adults Experiencing Homelessness in Seven U.S. Cities Using Interpretable Machine Learning. | Persons/cases affected by: intimate partner violence; stalking | intimate-partner relationship; specific status not reported/unclear | not reported/unclear | yes - IPV/domestic-abuse pathway | not reported/unclear | intimate partner violence; stalking | machine learning; predictive analytics/modeling | not reported/unclear |
| 69 | Awasekar D. et al. (2025) | Artificial Intelligence for Legal Assistance: A Prescriptive Analytics Model Integrating Social Emotional Learning for Assisting Victims of Domestic Violence in India | Persons/cases affected by: domestic violence/abuse | domestic/family relationship context; specific intimate-partner status not reported/unclear | not reported/unclear | yes - IPV/domestic-abuse pathway | not reported/unclear | domestic violence/abuse | artificial intelligence; chatbot/digital assistant; geospatial/search/speech/computer vision | police/legal/medico-legal; digital/online |
| 70 | Hui et al. (2026) | Perspectives and preferences of domestic violence survivors regarding digital platform and AI chatbot for help-seeking: A qualitative study. | Persons/cases affected by: intimate partner violence; domestic violence/abuse; family violence | intimate-partner relationship; specific status not reported/unclear | not reported/unclear | yes - IPV/domestic-abuse pathway | not reported/unclear | intimate partner violence; domestic violence/abuse; family violence | artificial intelligence; chatbot/digital assistant | digital/online |
| 71 | Verma P. et al. (2026) | Predictive Modeling of Domestic Violence in India: A Machine Learning Perspective | Persons/cases affected by: intimate partner violence; domestic violence/abuse; gender-based violence/violence against women | intimate-partner relationship; specific status not reported/unclear | yes | yes - IPV/domestic-abuse pathway | not reported/unclear | intimate partner violence; domestic violence/abuse; gender-based violence/violence against women | machine learning; deep learning; predictive analytics/modeling | digital/online |
| 72 | Hernandez-Zetina S. et al. (2026) | Developing a Predictive Model for Gender-Based Violence in Urban Areas Using Open Data | Persons/cases affected by: gender-based violence/violence against women | not reported/unclear | yes | not reported/unclear | not reported/unclear | gender-based violence/violence against women | machine learning; deep learning; predictive analytics/modeling; geospatial/search/speech/computer vision | not reported/unclear |
| 73 | Giorgio A. (2026) | A Smart App for the Prevention of Gender-Based Violence Using Artificial Intelligence | Persons/cases affected by: femicide/feminicide; gender-based violence/violence against women | not reported/unclear | yes | yes - IPV/domestic-abuse pathway | not reported/unclear | femicide/feminicide; gender-based violence/violence against women | artificial intelligence; geospatial/search/speech/computer vision | digital/online |
| 74 | Hui et al. (2025) | Leveraging GraphRAG with Large Language Models to Identify Help-Seeking Information Among Domestic Violence Survivors from Qualitative Interviews. | Persons/cases affected by: domestic violence/abuse | domestic/family relationship context; specific intimate-partner status not reported/unclear | not reported/unclear | yes - IPV/domestic-abuse pathway | not reported/unclear | domestic violence/abuse | deep learning; NLP/text mining; LLM/generative AI | digital/online |
| 75 | Zhang et al. (2025) | Probing Social Support and Self-Disclosure Within Chinese Online Domestic Violence Support Groups: Leveraging Multiple Machine Learning Approaches. | Persons/cases affected by: domestic violence/abuse | domestic/family relationship context; specific intimate-partner status not reported/unclear | not reported/unclear | yes - IPV/domestic-abuse pathway | not reported/unclear | domestic violence/abuse | machine learning; deep learning; NLP/text mining; geospatial/search/speech/computer vision | digital/online |
| 76 | Talwalk et al. (2025) | Domestic Abuse Survivor Assistance Through Forecasting and Engagement with Artificial Intelligence Solutions | Persons/cases affected by: intimate partner violence; domestic violence/abuse; coercive control | intimate-partner relationship; specific status not reported/unclear | not reported/unclear | yes - IPV/domestic-abuse pathway | not reported/unclear | intimate partner violence; domestic violence/abuse; coercive control | artificial intelligence; predictive analytics/modeling | not reported/unclear |
| 77 | Gonzálvez-Gallego N. et al. (2024) | Do search queries predict violence against women? A forecasting model based on Google Trends | Persons/cases affected by: intimate partner violence; domestic violence/abuse; femicide/feminicide; gender-based... | intimate-partner relationship; specific status not reported/unclear | yes | yes - IPV/domestic-abuse pathway | not reported/unclear | intimate partner violence; domestic violence/abuse; femicide/feminicide; gender-based... | predictive analytics/modeling; geospatial/search/speech/computer vision | not reported/unclear |
| 78 | Salehi et al. (2023) | Domestic violence risk prediction in Iran using a machine learning approach by analyzing Persian textual content in social media. | Persons/cases affected by: domestic violence/abuse; family violence | domestic/family relationship context; specific intimate-partner status not reported/unclear | not reported/unclear | yes - IPV/domestic-abuse pathway | not reported/unclear | domestic violence/abuse; family violence | machine learning; NLP/text mining; predictive analytics/modeling; geospatial/search/speech/computer vision | digital/online |
| 79 | Sisodia A. et al. (2023) | A Machine Learning Approach to Predict Poor Mental Health of Intimate Partner Violence Survivors | Persons/cases affected by: intimate partner violence; gender-based violence/violence against women | intimate-partner relationship; specific status not reported/unclear | yes | yes - IPV/domestic-abuse pathway | not reported/unclear | intimate partner violence; gender-based violence/violence against women | machine learning; predictive analytics/modeling; geospatial/search/speech/computer vision | digital/online |
| 80 | Reyner-Fuentes et al. (2023) | Prediction of the Gender-based Violence Victim Condition using Speech: What do Machine Learning Models rely on? | Persons/cases affected by: gender-based violence/violence against women | not reported/unclear | yes | not reported/unclear | not reported/unclear | gender-based violence/violence against women | artificial intelligence; machine learning; deep learning; geospatial/search/speech/computer vision | not reported/unclear |
| 81 | L. R. Abdulkareem et al. (2022) | Using ANN to Predict Gender-Based Violence in Iraq: How AI and data mining technologies revolutionized social networks to make a safer world | Persons/cases affected by: gender-based violence/violence against women | not reported/unclear | yes | not reported/unclear | not reported/unclear | gender-based violence/violence against women | artificial intelligence; machine learning; deep learning; predictive analytics/modeling; data mining... | not reported/unclear |
| 82 | Victor et al. (2021) | Automated identification of domestic violence in written child welfare records: Leveraging text mining and machine learning to enhance social work research and evaluation. | Persons/cases affected by: domestic violence/abuse | domestic/family relationship context; specific intimate-partner status not reported/unclear | not reported/unclear | yes - IPV/domestic-abuse pathway | not reported/unclear | domestic violence/abuse | machine learning; deep learning; NLP/text mining; predictive analytics/modeling; record/data linkage | multi-agency/linked data |
| 83 | Castorena C.M. et al. (2021) | Deep neural network for gender-based violence detection on twitter messages | Persons/cases affected by: gender-based violence/violence against women | not reported/unclear | yes | not reported/unclear | not reported/unclear | gender-based violence/violence against women | machine learning; deep learning; geospatial/search/speech/computer vision | digital/online |
| 84 | Rodríguez-Rodríguez I. et al. (2020) | Modeling and forecasting gender-based violence through machine learning techniques | Persons/cases affected by: intimate partner violence; domestic violence/abuse; gender-based violence/violence against women | intimate-partner relationship; specific status not reported/unclear | yes | yes - IPV/domestic-abuse pathway | not reported/unclear | intimate partner violence; domestic violence/abuse; gender-based violence/violence against women | machine learning; deep learning; predictive analytics/modeling | digital/online |
| 85 | Subramani et al. (2019) | Deep Learning for Multi-Class Identification from Domestic Violence Online Posts | Persons/cases affected by: domestic violence/abuse; gender-based violence/violence against women | domestic/family relationship context; specific intimate-partner status not reported/unclear | yes | yes - IPV/domestic-abuse pathway | not reported/unclear | domestic violence/abuse; gender-based violence/violence against women | deep learning; geospatial/search/speech/computer vision | digital/online |
| 86 | S. Subramani et al. (2018) | Domestic violence crisis identification from facebook posts based on deep learning | Persons/cases affected by: domestic violence/abuse | domestic/family relationship context; specific intimate-partner status not reported/unclear | not reported/unclear | yes - IPV/domestic-abuse pathway | not reported/unclear | domestic violence/abuse | machine learning; deep learning; geospatial/search/speech/computer vision | digital/online |
| 87 | Petering R. et al. (2018) | Artificial Intelligence to Predict Intimate Partner Violence Perpetration | Persons/cases affected by: intimate partner violence; domestic violence/abuse | intimate-partner relationship; specific status not reported/unclear | not reported/unclear | yes - IPV/domestic-abuse pathway | not reported/unclear | intimate partner violence; domestic violence/abuse | artificial intelligence; machine learning | not reported/unclear |
| 88 | Van Hightower et al. (2000) | Predictive models of domestic violence and fear of intimate partners among migrant and seasonal farm worker women. | Persons/cases affected by: domestic violence/abuse; family violence | intimate-partner relationship; specific status not reported/unclear | yes | yes - IPV/domestic-abuse pathway | not reported/unclear | domestic violence/abuse; family violence | deep learning; predictive analytics/modeling | not reported/unclear |
| 89 | Marisetti M. et al. (2026) | Real Time Sentiment Analysis of Domestic Violence Tweets | Persons/cases affected by: intimate partner violence; domestic violence/abuse | intimate-partner relationship; specific status not reported/unclear | not reported/unclear | yes - IPV/domestic-abuse pathway | not reported/unclear | intimate partner violence; domestic violence/abuse | artificial intelligence; machine learning; geospatial/search/speech/computer vision | not reported/unclear |
| 90 | Le B.L. et al. (2026) | Beyond physical violence: A machine learning framework for predicting IPV victimisation using multidimensional predictors | Persons/cases affected by: intimate partner violence; domestic violence/abuse; femicide/feminicide; lethality/severe escalation | intimate-partner relationship; specific status not reported/unclear | yes | yes - IPV/domestic-abuse pathway | not reported/unclear | intimate partner violence; domestic violence/abuse; femicide/feminicide; lethality/severe escalation | machine learning; predictive analytics/modeling | not reported/unclear |
| 91 | Singh S. et al. (2026) | Machine Learning in Domestic Violence: Current Trends and Future Prospectives | Persons/cases affected by: domestic violence/abuse | domestic/family relationship context; specific intimate-partner status not reported/unclear | not reported/unclear | yes - IPV/domestic-abuse pathway | not reported/unclear | domestic violence/abuse | artificial intelligence; machine learning; deep learning | not reported/unclear |
| 92 | Sood V. (2025) | Empowering Interventions: AI and Machine Learning Solutions for Predicting and Managing Domestic Violence | Persons/cases affected by: domestic violence/abuse | domestic/family relationship context; specific intimate-partner status not reported/unclear | not reported/unclear | yes - IPV/domestic-abuse pathway | not reported/unclear | domestic violence/abuse | artificial intelligence; machine learning; deep learning; predictive analytics/modeling; decision... | not reported/unclear |
| 93 | Hui et al. (2024) | Predicting the Information Need for Domestic Violence Survivors Based on the Fine-Tuned Large Language Model. | Persons/cases affected by: intimate partner violence; domestic violence/abuse | intimate-partner relationship; specific status not reported/unclear | not reported/unclear | yes - IPV/domestic-abuse pathway | not reported/unclear | intimate partner violence; domestic violence/abuse | artificial intelligence; machine learning; NLP/text mining; LLM/generative AI; predictive analytics/modeling | digital/online |
| 94 | Sachdeva et al. (2024) | A Traumatic Brain Injury Prescreening Tool for Intimate Partner Violence Patients Using Initial Clinical Reports and Machine Learning. | Persons/cases affected by: intimate partner violence; domestic violence/abuse | intimate-partner relationship; specific status not reported/unclear | not reported/unclear | yes - IPV/domestic-abuse pathway | not reported/unclear | intimate partner violence; domestic violence/abuse | machine learning; EHR/electronic health records | healthcare/digital health |
| 95 | Szyfer Lipinsky et al. (2024) | Predicting psychopathology in Jewish ultra-orthodox IPV survivors: A machine learning approach. | Persons/cases affected by: intimate partner violence | intimate-partner relationship; specific status not reported/unclear | not reported/unclear | yes - IPV/domestic-abuse pathway | not reported/unclear | intimate partner violence | machine learning; predictive analytics/modeling | digital/online |
| 96 | Contreras-Jiménez et al. (2024) | Mapping Gender-Based Violence: Integrating Machine Learning and Geospatial Analysis in Mexico City | Persons/cases affected by: gender-based violence/violence against women | not reported/unclear | yes | not reported/unclear | not reported/unclear | gender-based violence/violence against women | machine learning; predictive analytics/modeling; data mining; geospatial/search/speech/computer vision | digital/online |
| 97 | Rahman et al. (2023) | A comparative study of machine learning algorithms for predicting domestic violence vulnerability in Liberian women. | Persons/cases affected by: intimate partner violence; domestic violence/abuse; gender-based violence/violence against women | intimate-partner relationship; specific status not reported/unclear | yes | yes - IPV/domestic-abuse pathway | not reported/unclear | intimate partner violence; domestic violence/abuse; gender-based violence/violence against women | machine learning; predictive analytics/modeling | not reported/unclear |
| 98 | Chen et al. (2023) | Using machine learning to estimate the incidence rate of intimate partner violence. | Persons/cases affected by: intimate partner violence; domestic violence/abuse | intimate-partner relationship; specific status not reported/unclear | not reported/unclear | yes - IPV/domestic-abuse pathway | not reported/unclear | intimate partner violence; domestic violence/abuse | machine learning | population/public health |
| 99 | Todorovic et al. (2022) | Prevalence, increase and predictors of family violence during the COVID-19 pandemic, using modern machine learning approaches. | Persons/cases affected by: intimate partner violence; family violence | intimate-partner relationship; specific status not reported/unclear | not reported/unclear | yes - IPV/domestic-abuse pathway | not reported/unclear | intimate partner violence; family violence | machine learning | digital/online; population/public health |
| 100 | Kennedy et al. (2023) | Insights from linking police domestic abuse data and health data in South Wales, UK: a linked routine data analysis using decision tree classification. | Persons/cases affected by: domestic violence/abuse; stalking | domestic/family relationship context; specific intimate-partner status not reported/unclear | not reported/unclear | yes - IPV/domestic-abuse pathway | not reported/unclear | domestic violence/abuse; stalking | record/data linkage | police/legal/medico-legal; multi-agency/linked data |
| 101 | Tabaie et al. (2022) | A Novel Technique to Identify Intimate Partner Violence in a Hospital Setting. | Persons/cases affected by: intimate partner violence | intimate-partner relationship; specific status not reported/unclear | not reported/unclear | yes - IPV/domestic-abuse pathway | not reported/unclear | intimate partner violence | deep learning; NLP/text mining; EHR/electronic health records | healthcare/digital health |
| 102 | D. D. Awasekar et al. (2025) | AI-Driven Extraction and Verification of Semi-Categorized Legal Documents: The LAMP2 4.0 Retrieval-Augmented Generation Framework for Statute-Aligned Legal Relief Prediction | Persons/cases affected by: domestic violence/abuse | domestic/family relationship context; specific intimate-partner status not reported/unclear | not reported/unclear | yes - IPV/domestic-abuse pathway | not reported/unclear | domestic violence/abuse | artificial intelligence; NLP/text mining; LLM/generative AI | police/legal/medico-legal |
| 103 | Botelle et al. (2022) | Can natural language processing models extract and classify instances of interpersonal violence in mental healthcare electronic records: an applied evaluative study. | Persons/cases affected by: domestic violence/abuse | domestic/family relationship context; specific intimate-partner status not reported/unclear | not reported/unclear | yes - IPV/domestic-abuse pathway | not reported/unclear | domestic violence/abuse | artificial intelligence; deep learning; NLP/text mining; data mining; EHR/electronic health records | healthcare/digital health; digital/online |
| 104 | González et al. (2021) | A sentiment analysis and unsupervised learning approach to digital violence a... | Persons/cases affected by: family violence; homicide/lethality; violence against women | domestic/family relationship context; specific intimate-partner status not reported/unclear | yes | yes - IPV/domestic-abuse pathway | not reported/unclear | family violence; homicide/lethality; violence against women | AI/algorithmic tool | digital/online |
| 105 | V. Ezhumalai et al. (2025) | Artificial Intelligence keeps women safe: Preventing online and domestic Viol... | Persons/cases affected by: domestic violence/abuse; harassment | domestic/family relationship context; specific intimate-partner status not reported/unclear | yes | yes - IPV/domestic-abuse pathway | not reported/unclear | domestic violence/abuse; harassment | NLP; machine learning; predictive analytics/risk prediction | digital/online |
| 106 | S. Talwalkar et al. (2023) | Designing User-Centered Artificial Intelligence to Assist in Recovery from Do... | Persons/cases affected by: IPV; domestic violence/abuse | domestic/family relationship context; specific intimate-partner status not reported/unclear | not reported/unclear | yes - IPV/domestic-abuse pathway | not reported/unclear | IPV; domestic violence/abuse | machine learning; data mining | not reported/unclear |
| 107 | Fathima K.S. et al. (2023) | Domestic Violence Detection System Using Natural Language Processing | Persons/cases affected by: domestic violence/abuse | domestic/family relationship context; specific intimate-partner status not reported/unclear | not reported/unclear | yes - IPV/domestic-abuse pathway | not reported/unclear | domestic violence/abuse | NLP; machine learning | not reported/unclear |
| 108 | Ngũnjiri et al. (2023) | Utilizing User Preferences in Designing the AGILE (Accelerating Access to Gen... | Persons/cases affected by: IPV; homicide/lethality; GBV | not reported/unclear | not reported/unclear | yes - IPV/domestic-abuse pathway | not reported/unclear | IPV; homicide/lethality; GBV | NLP; chatbot/virtual assistant | digital/online |
| 109 | A. K. Tiwari et al. (2024) | Leveraging Artificial Intelligence to Address Domestic Violence Against Women... | Persons/cases affected by: domestic violence/abuse; violence against women | domestic/family relationship context; specific intimate-partner status not reported/unclear | yes | yes - IPV/domestic-abuse pathway | not reported/unclear | domestic violence/abuse; violence against women | NLP; machine learning; predictive analytics/risk prediction; chatbot/virtual assistant | digital/online |
| 110 | P. P. Shifidi et al. (2023) | Machine Learning-Based Analytical Process for Predicting the Occurrence of Ge... | Persons/cases affected by: IPV; femicide; GBV | not reported/unclear | yes | yes - IPV/domestic-abuse pathway | not reported/unclear | IPV; femicide; GBV | machine learning | not reported/unclear |
| 111 | R. Roy et al. (2023) | Meta-Analysis of Artificial Intelligence Solution for Prevention of Violence ... | Persons/cases affected by: violence against women; harassment | not reported/unclear | yes | not reported/unclear | not reported/unclear | violence against women; harassment | machine learning; chatbot/virtual assistant; EHR-based identification | healthcare/digital health; digital/online |
| 112 | Soldevilla et al. (2021) | Natural language processing through BERT for identifying gender-based violenc... | Persons/cases affected by: domestic violence/abuse; GBV; violence against women; harassment | domestic/family relationship context; specific intimate-partner status not reported/unclear | yes | yes - IPV/domestic-abuse pathway | not reported/unclear | domestic violence/abuse; GBV; violence against women; harassment | NLP; machine learning | not reported/unclear |
| 113 | V. Itham et al. (2024) | Real-Time Sentiment Analysis of Domestic Violence Tweets: Informing Intervent... | Persons/cases affected by: IPV; domestic violence/abuse | domestic/family relationship context; specific intimate-partner status not reported/unclear | not reported/unclear | yes - IPV/domestic-abuse pathway | not reported/unclear | IPV; domestic violence/abuse | NLP; deep learning; machine learning; text mining/topic modelling | not reported/unclear |
| 114 | Stephanie E.M.A. et al. (2024) | Study of violence against women and its characteristics through the applicati... | Persons/cases affected by: domestic violence/abuse; femicide; GBV; violence against women | domestic/family relationship context; specific intimate-partner status not reported/unclear | yes | yes - IPV/domestic-abuse pathway | not reported/unclear | domestic violence/abuse; femicide; GBV; violence against women | NLP; deep learning; machine learning; text mining/topic modelling | digital/online |
| 115 | Alzyout M. et al. (2021) | Sentiment Analysis of Arabic Tweets about Violence against Women using Machin... | Persons/cases affected by: violence against women | not reported/unclear | yes | not reported/unclear | not reported/unclear | violence against women | deep learning; machine learning | not reported/unclear |
| 116 | D. D. Awasekar et al. (2025) | SWATI AI : Advancing AI Models from Rule-Based Frameworks to NLP-Driven Presc... | Persons/cases affected by: domestic violence/abuse | domestic/family relationship context; specific intimate-partner status not reported/unclear | not reported/unclear | yes - IPV/domestic-abuse pathway | not reported/unclear | domestic violence/abuse | LLM; NLP; machine learning; chatbot/virtual assistant | digital/online |
| 117 | Castro et al. (2019) | User acceptance of predictive analytics on violence against women and childre... | Persons/cases affected by: violence against women | not reported/unclear | yes | not reported/unclear | not reported/unclear | violence against women | predictive analytics/risk prediction | not reported/unclear |
| 118 | Yallico Arias T. et al. (2022) | Automatic Detection of Levels of Intimate Partner Violence Against Women with... | Persons/cases affected by: IPV; domestic violence/abuse; femicide; violence against women | intimate-partner relationship; specific status not reported/unclear | yes | yes - IPV/domestic-abuse pathway | not reported/unclear | IPV; domestic violence/abuse; femicide; violence against women | NLP; deep learning; machine learning | not reported/unclear |
| 119 | Gutam B.G. et al. (2025) | A Machine Learning and Community-Driven Approach for Feminist Cyber Resistanc... | Persons/cases affected by: GBV; stalking; harassment | not reported/unclear | not reported/unclear | yes - IPV/domestic-abuse pathway | not reported/unclear | GBV; stalking; harassment | machine learning | digital/online |
| 120 | Karystianis et al. (2018) | Automatic Extraction of Mental Health Disorders From Domestic Violence Police... | Persons/cases affected by: domestic violence/abuse; violence against women | domestic/family relationship context; specific intimate-partner status not reported/unclear | yes | yes - IPV/domestic-abuse pathway | not reported/unclear | domestic violence/abuse; violence against women | text mining/topic modelling | healthcare/digital health; police/legal/medico-legal |
| 121 | O. M. Cumbicus-Pineda et al. (2021) | Data Mining to Determine the Causes of Gender-Based Violence against Women in... | Persons/cases affected by: domestic violence/abuse; GBV; violence against women | domestic/family relationship context; specific intimate-partner status not reported/unclear | yes | yes - IPV/domestic-abuse pathway | not reported/unclear | domestic violence/abuse; GBV; violence against women | NLP; deep learning; machine learning; data mining | not reported/unclear |
| 122 | Ismail O. et al. (2025) | Digitalizing Prevention and Detection of Violence Against Women and Girls Thr... | Persons/cases affected by: domestic violence/abuse; GBV; violence against women; stalking; harassment | domestic/family relationship context; specific intimate-partner status not reported/unclear | yes | yes - IPV/domestic-abuse pathway | not reported/unclear | domestic violence/abuse; GBV; violence against women; stalking; harassment | NLP; deep learning; machine learning; predictive analytics/risk prediction; chatbot/virtual assistant | digital/online |
| 123 | Vowels et al. (2026) | Large language models for psychosocial risk assessment: A multi-method evalua... | Persons/cases affected by: IPV | not reported/unclear | not reported/unclear | not reported/unclear | not reported/unclear | IPV | LLM; chatbot/virtual assistant | digital/online |
| 124 | Dehingia et al. (2022) | Help seeking behavior by women experiencing intimate partner violence in indi... | Persons/cases affected by: IPV; GBV | intimate-partner relationship; specific status not reported/unclear | yes | yes - IPV/domestic-abuse pathway | not reported/unclear | IPV; GBV | machine learning | not reported/unclear |
| 125 | Salehi et al. (2024) | Classification of domestic violence Persian textual content in social media b... | Persons/cases affected by: domestic violence/abuse; violence against women | domestic/family relationship context; specific intimate-partner status not reported/unclear | yes | yes - IPV/domestic-abuse pathway | not reported/unclear | domestic violence/abuse; violence against women | text mining/topic modelling; EHR-based identification | healthcare/digital health; digital/online |

Supplementary Table 2B. Technical, implementation and governance charting fields

| **Supplementary record no.** | **Author/year** | **AI/digital method category** | **Data source** | **Country/region** | **Intended function** | **Target outcome** | **Femicide/lethality relevance** | **Validation status** | **Implementation or real-world evaluation status** | **Governance/ethics/accountability issues** | **Main relevance to the review** | **Notes/uncertainty** |
| --- | --- | --- | --- | --- | --- | --- | --- | --- | --- | --- | --- | --- |
| 1 | Garcia-Vergara et al. (2023) | artificial intelligence; machine learning; NLP/text mining; predictive analytics/modeling | police/legal/judicial or mortality records | Spain | detection/identification; prediction/forecasting; prevention/intervention/referral | femicide/feminicide; lethality/severe escalation | direct femicide/homicide/lethality or severe-escalation relevance | model development/validation | model development/evaluation/validation | risk of false reassurance/false positives requires human review | Links femicide/feminicide; lethality/severe escalation with detection/identification; prediction/forecasting; prevention/intervention/referral through artificial intelligence... | Coded from available bibliographic/charting information; not reported/unclear means not inferable from the available record-level charting. |
| 2 | Xue et al. (2020) | machine learning; NLP/text mining; geospatial/search/speech/computer vision | healthcare/EHR/clinical records | United States; Canada; China | detection/identification | intimate partner violence; domestic violence/abuse; family violence; coercive control... | indirect IPV/domestic-abuse risk-pathway relevance | model development/validation | model development/evaluation/validation | risk of false reassurance/false positives requires human review | Links intimate partner violence; domestic violence/abuse; family violence; coercive control; gender-based violence/violence against women with detection/identification; risk... | Coded from available bibliographic/charting information; not reported/unclear means not inferable from the available record-level charting. |
| 3 | Verrey et al. (2023) | artificial intelligence; machine learning; predictive analytics/modeling; decision support/risk stratification | police/legal/judicial or mortality records | United Kingdom; Israel | detection/identification; risk assessment/stratification | domestic violence/abuse; intimate partner homicide/domestic homicide; lethality/severe escalation | direct femicide/homicide/lethality or severe-escalation relevance | model development/validation | model development/evaluation/validation | risk of false reassurance/false positives requires human review | Links domestic violence/abuse; intimate partner homicide/domestic homicide; lethality/severe escalation with detection/identification; risk assessment/stratification... | Coded from available bibliographic/charting information; not reported/unclear means not inferable from the available record-level charting. |
| 4 | da Silva B.B.A. et al. (2026) | machine learning; deep learning; predictive analytics/modeling | police/legal/judicial or mortality records; online/social media/digital platform | United States; Spain; Brazil | detection/identification; prediction/forecasting; prevention/intervention/referral | femicide/feminicide; gender-based violence/violence against women | direct femicide/homicide/lethality or severe-escalation relevance | review | review evidence | risk of false reassurance/false positives requires human review | Links femicide/feminicide; gender-based violence/violence against women with detection/identification; prediction/forecasting; prevention/intervention/referral through machine... | Coded from available bibliographic/charting information; not reported/unclear means not inferable from the available record-level charting. |
| 5 | Peddireddy et al. (2024) | machine learning | police/legal/judicial or mortality records | United States | detection/identification; risk assessment/stratification; prediction/forecasting | intimate partner violence; intimate partner homicide/domestic homicide | direct femicide/homicide/lethality or severe-escalation relevance | model development/validation | model development/evaluation/validation | risk of false reassurance/false positives requires human review | Links intimate partner violence; intimate partner homicide/domestic homicide with detection/identification; risk assessment/stratification; prediction/forecasting... | Coded from available bibliographic/charting information; not reported/unclear means not inferable from the available record-level charting. |
| 6 | Trias Capella M.E. et al. (2024) | artificial intelligence | police/legal/judicial or mortality records | Spain | detection/identification; prevention/intervention/referral | femicide/feminicide; gender-based violence/violence against women | direct femicide/homicide/lethality or severe-escalation relevance | not reported/unclear | not reported/unclear or conceptual/technical | not reported/unclear from available charting | Links femicide/feminicide; gender-based violence/violence against women with detection/identification; prevention/intervention/referral through artificial intelligence. | Coded from available bibliographic/charting information; not reported/unclear means not inferable from the available record-level charting. |
| 7 | Hui et al. (2023) | NLP/text mining; geospatial/search/speech/computer vision | healthcare/EHR/clinical records; online/social media/digital platform | United States; China | detection/identification; prediction/forecasting; prevention/intervention/referral | intimate partner violence | indirect IPV/domestic-abuse risk-pathway relevance | model development/validation | model development/evaluation/validation | risk of false reassurance/false positives requires human review | Links intimate partner violence with detection/identification; prediction/forecasting; prevention/intervention/referral through NLP/text mining; geospatial/search/speech/computer... | Coded from available bibliographic/charting information; not reported/unclear means not inferable from the available record-level charting. |
| 8 | Harris et al. (2026) | artificial intelligence; NLP/text mining; data mining; EHR/electronic health records | healthcare/EHR/clinical records; survey/population or administrative data | United States | detection/identification; screening; risk assessment/stratification; early warning/surveillance; prevention/intervention/referral | intimate partner violence | indirect IPV/domestic-abuse risk-pathway relevance | model development/validation | model development/evaluation/validation | risk of false reassurance/false positives requires human review | Links intimate partner violence with detection/identification; screening; risk assessment/stratification; early warning/surveillance; prevention/intervention/referral... | Coded from available bibliographic/charting information; not reported/unclear means not inferable from the available record-level charting. |
| 9 | More S. et al. (2023) | machine learning; predictive analytics/modeling; data mining | police/legal/judicial or mortality records | India; Peru | detection/identification; prediction/forecasting | intimate partner violence; femicide/feminicide; gender-based violence/violence against women | direct femicide/homicide/lethality or severe-escalation relevance | model development/validation | model development/evaluation/validation | risk of false reassurance/false positives requires human review | Links intimate partner violence; femicide/feminicide; gender-based violence/violence against women with detection/identification; prediction/forecasting... | Coded from available bibliographic/charting information; not reported/unclear means not inferable from the available record-level charting. |
| 10 | Karystianis et al. (2022) | NLP/text mining | police/legal/judicial or mortality records | United States; Australia; United Kingdom | detection/identification; early warning/surveillance; prevention/intervention/referral; legal/ethical governance | domestic violence/abuse | indirect IPV/domestic-abuse risk-pathway relevance | not reported/unclear | not reported/unclear or conceptual/technical | governance/ethics explicitly indicated in charting | Links domestic violence/abuse with detection/identification; early warning/surveillance; prevention/intervention/referral; legal/ethical governance through NLP/text mining. | Coded from available bibliographic/charting information; not reported/unclear means not inferable from the available record-level charting. |
| 11 | Karystianis et al. (2022) | NLP/text mining; record/data linkage | healthcare/EHR/clinical records; police/legal/judicial or mortality records; linked administrative data | Australia; United Kingdom; South Wales | detection/identification; risk assessment/stratification; early warning/surveillance; inter-agency coordination; legal/ethical governance | domestic violence/abuse | indirect IPV/domestic-abuse risk-pathway relevance | observational/data-linkage study | observational/data-linkage evidence | governance/ethics explicitly indicated in charting; risk of false reassurance/false positives requires human review | Links domestic violence/abuse with detection/identification; risk assessment/stratification; early warning/surveillance; inter-agency coordination; legal/ethical governance... | Coded from available bibliographic/charting information; not reported/unclear means not inferable from the available record-level charting. |
| 12 | Randell et al. (2022) | EHR/electronic health records | healthcare/EHR/clinical records | not reported | detection/identification | intimate partner violence; stalking | indirect IPV/domestic-abuse risk-pathway relevance | qualitative study | qualitative/exploratory evidence | not reported/unclear from available charting | Links intimate partner violence; stalking with detection/identification through EHR/electronic health records. | Coded from available bibliographic/charting information; not reported/unclear means not inferable from the available record-level charting. |
| 13 | Gu et al. (2026) | machine learning; deep learning; NLP/text mining; LLM/generative AI; predictive analytics/modeling; decision... | not reported/unclear from available charting | United States | detection/identification; screening; prediction/forecasting; decision support/triage | intimate partner violence; domestic violence/abuse | indirect IPV/domestic-abuse risk-pathway relevance | model development/validation | model development/evaluation/validation | risk of false reassurance/false positives requires human review | Links intimate partner violence; domestic violence/abuse with detection/identification; screening; prediction/forecasting; decision support/triage... | Coded from available bibliographic/charting information; not reported/unclear means not inferable from the available record-level charting. |
| 14 | Lenert et al. (2024) | decision support/risk stratification; EHR/electronic health records | healthcare/EHR/clinical records | not reported | detection/identification; screening; risk assessment/stratification; early warning/surveillance; decision support/triage | intimate partner violence | indirect IPV/domestic-abuse risk-pathway relevance | clinical trial / implementation trial | implementation or trial evidence reported | risk of false reassurance/false positives requires human review | Links intimate partner violence with detection/identification; screening; risk assessment/stratification; early warning/surveillance; decision support/triage... | Coded from available bibliographic/charting information; not reported/unclear means not inferable from the available record-level charting. |
| 15 | Karystianis G. et al. (2024) | NLP/text mining | police/legal/judicial or mortality records | United States; Australia; United Kingdom | detection/identification | domestic violence/abuse; family violence; coercive control | indirect IPV/domestic-abuse risk-pathway relevance | not reported/unclear | not reported/unclear or conceptual/technical | not reported/unclear from available charting | Links domestic violence/abuse; family violence; coercive control with detection/identification through NLP/text mining. | Coded from available bibliographic/charting information; not reported/unclear means not inferable from the available record-level charting. |
| 16 | Syed et al. (2021) | deep learning; EHR/electronic health records | healthcare/EHR/clinical records | United Kingdom | detection/identification; early warning/surveillance; prediction/forecasting; prevention/intervention/referral | intimate partner violence; family violence | indirect IPV/domestic-abuse risk-pathway relevance | review | review evidence | risk of false reassurance/false positives requires human review | Links intimate partner violence; family violence with detection/identification; prediction/forecasting; early warning/surveillance; prevention/intervention/referral... | Coded from available bibliographic/charting information; not reported/unclear means not inferable from the available record-level charting. |
| 17 | Adily et al. (2021) | artificial intelligence; NLP/text mining | police/legal/judicial or mortality records | not reported | detection/identification; prediction/forecasting | domestic violence/abuse; family violence | indirect IPV/domestic-abuse risk-pathway relevance | not reported/unclear | not reported/unclear or conceptual/technical | risk of false reassurance/false positives requires human review | Links domestic violence/abuse; family violence with detection/identification; prediction/forecasting through artificial intelligence; NLP/text mining. | Coded from available bibliographic/charting information; not reported/unclear means not inferable from the available record-level charting. |
| 18 | Karystianis et al. (2021) | deep learning; NLP/text mining; predictive analytics/modeling; record/data linkage | healthcare/EHR/clinical records; police/legal/judicial or mortality records; online/social media/digital platform; linked administrative data | United States; Australia; United Kingdom | detection/identification; risk assessment/stratification; prediction/forecasting | intimate partner violence; domestic violence/abuse; family violence | indirect IPV/domestic-abuse risk-pathway relevance | model development/validation | model development/evaluation/validation | risk of false reassurance/false positives requires human review | Links intimate partner violence; domestic violence/abuse; family violence with detection/identification; risk assessment/stratification; prediction/forecasting through deep... | Coded from available bibliographic/charting information; not reported/unclear means not inferable from the available record-level charting. |
| 19 | Bhargava et al. (2011) | predictive analytics/modeling; EHR/electronic health records | healthcare/EHR/clinical records | not reported | detection/identification; prediction/forecasting; prevention/intervention/referral; legal/ethical governance | intimate partner violence | indirect IPV/domestic-abuse risk-pathway relevance | model development/validation | model development/evaluation/validation | governance/ethics explicitly indicated in charting; risk of false reassurance/false positives requires human review | Links intimate partner violence with detection/identification; prediction/forecasting; prevention/intervention/referral; legal/ethical governance through predictive... | Coded from available bibliographic/charting information; not reported/unclear means not inferable from the available record-level charting. |
| 20 | Tabaie et al. (2024) | data mining; EHR/electronic health records | healthcare/EHR/clinical records | United States | detection/identification; screening | intimate partner violence | indirect IPV/domestic-abuse risk-pathway relevance | not reported/unclear | not reported/unclear or conceptual/technical | not reported/unclear from available charting | Links intimate partner violence with detection/identification; screening through data mining; EHR/electronic health records. | Coded from available bibliographic/charting information; not reported/unclear means not inferable from the available record-level charting. |
| 21 | Hui et al. (2023) | machine learning; NLP/text mining; EHR/electronic health records; geospatial/search/speech/computer vision | healthcare/EHR/clinical records | United States; Australia | detection/identification; prediction/forecasting | intimate partner violence; domestic violence/abuse | indirect IPV/domestic-abuse risk-pathway relevance | review | review evidence | risk of false reassurance/false positives requires human review | Links intimate partner violence; domestic violence/abuse with detection/identification; prediction/forecasting through machine learning; NLP/text mining; EHR/electronic health... | Coded from available bibliographic/charting information; not reported/unclear means not inferable from the available record-level charting. |
| 22 | Yılmaz et al. (2023) | EHR/electronic health records | healthcare/EHR/clinical records | United States; Turkey; Kenya | detection/identification; screening | intimate partner violence | indirect IPV/domestic-abuse risk-pathway relevance | not reported/unclear | not reported/unclear or conceptual/technical | not reported/unclear from available charting | Links intimate partner violence with detection/identification; screening through EHR/electronic health records. | Coded from available bibliographic/charting information; not reported/unclear means not inferable from the available record-level charting. |
| 23 | Johnson et al. (2023) | record/data linkage; EHR/electronic health records | healthcare/EHR/clinical records; linked administrative data | United Kingdom | detection/identification; risk assessment/stratification; prevention/intervention/referral | domestic violence/abuse | indirect IPV/domestic-abuse risk-pathway relevance | review | review evidence | risk of false reassurance/false positives requires human review | Links domestic violence/abuse with detection/identification; risk assessment/stratification; prevention/intervention/referral through record/data linkage; EHR/electronic health... | Coded from available bibliographic/charting information; not reported/unclear means not inferable from the available record-level charting. |
| 24 | Whitten et al. (2022) | record/data linkage | survey/population or administrative data; linked administrative data | Australia; South Wales | prevention/intervention/referral | domestic violence/abuse | indirect IPV/domestic-abuse risk-pathway relevance | observational/data-linkage study | observational/data-linkage evidence | not reported/unclear from available charting | Links domestic violence/abuse with prevention/intervention/referral through record/data linkage. | Coded from available bibliographic/charting information; not reported/unclear means not inferable from the available record-level charting. |
| 25 | Adily et al. (2021) | NLP/text mining | police/legal/judicial or mortality records | Australia | detection/identification | intimate partner violence; domestic violence/abuse; family violence; gender-based... | indirect IPV/domestic-abuse risk-pathway relevance | review | review evidence | not reported/unclear from available charting | Links intimate partner violence; domestic violence/abuse; family violence; gender-based violence/violence against women; stalking with detection/identification through NLP/text... | Coded from available bibliographic/charting information; not reported/unclear means not inferable from the available record-level charting. |
| 26 | Liu et al. (2020) | data mining; EHR/electronic health records | healthcare/EHR/clinical records | United States | detection/identification; screening | intimate partner violence; domestic violence/abuse | indirect IPV/domestic-abuse risk-pathway relevance | observational/data-linkage study | observational/data-linkage evidence | not reported/unclear from available charting | Links intimate partner violence; domestic violence/abuse with detection/identification; screening through data mining; EHR/electronic health records. | Coded from available bibliographic/charting information; not reported/unclear means not inferable from the available record-level charting. |
| 27 | Karystianis et al. (2020) | NLP/text mining | healthcare/EHR/clinical records; police/legal/judicial or mortality records; survey/population or administrative data | United States; Australia; United Kingdom | detection/identification; prevention/intervention/referral | domestic violence/abuse | indirect IPV/domestic-abuse risk-pathway relevance | not reported/unclear | not reported/unclear or conceptual/technical | not reported/unclear from available charting | Links domestic violence/abuse with detection/identification; prevention/intervention/referral through NLP/text mining. | Coded from available bibliographic/charting information; not reported/unclear means not inferable from the available record-level charting. |
| 28 | Karystianis et al. (2019) | NLP/text mining | healthcare/EHR/clinical records; police/legal/judicial or mortality records | Australia; United Kingdom; South Wales | detection/identification | domestic violence/abuse; gender-based violence/violence against women | indirect IPV/domestic-abuse risk-pathway relevance | not reported/unclear | not reported/unclear or conceptual/technical | not reported/unclear from available charting | Links domestic violence/abuse; gender-based violence/violence against women with detection/identification through NLP/text mining. | Coded from available bibliographic/charting information; not reported/unclear means not inferable from the available record-level charting. |
| 29 | Karakurt et al. (2017) | data mining; EHR/electronic health records | healthcare/EHR/clinical records | United States | detection/identification; prevention/intervention/referral | intimate partner violence; domestic violence/abuse | indirect IPV/domestic-abuse risk-pathway relevance | not reported/unclear | not reported/unclear or conceptual/technical | not reported/unclear from available charting | Links intimate partner violence; domestic violence/abuse with detection/identification; prevention/intervention/referral through data mining; EHR/electronic health records. | Coded from available bibliographic/charting information; not reported/unclear means not inferable from the available record-level charting. |
| 30 | Kasa et al. (2024) | EHR/electronic health records | healthcare/EHR/clinical records | not reported | detection/identification; screening; decision support/triage; prevention/intervention/referral | intimate partner violence; domestic violence/abuse | indirect IPV/domestic-abuse risk-pathway relevance | qualitative study | qualitative/exploratory evidence | governance/ethics explicitly indicated in charting; risk of false reassurance/false positives requires human review | Links intimate partner violence; domestic violence/abuse with detection/identification; screening; decision support/triage; prevention/intervention/referral; legal/ethical... | Coded from available bibliographic/charting information; not reported/unclear means not inferable from the available record-level charting. |
| 31 | Dogan et al. (2022) | artificial intelligence; machine learning; NLP/text mining | not reported/unclear from available charting | United States | detection/identification; prediction/forecasting | femicide/feminicide; gender-based violence/violence against women; lethality/severe escalation | direct femicide/homicide/lethality or severe-escalation relevance | qualitative study | qualitative/exploratory evidence | risk of false reassurance/false positives requires human review | Links femicide/feminicide; gender-based violence/violence against women; lethality/severe escalation with detection/identification; prediction/forecasting; early... | Coded from available bibliographic/charting information; not reported/unclear means not inferable from the available record-level charting. |
| 32 | Prakash et al. (2024) | deep learning; predictive analytics/modeling; decision support/risk stratification; EHR/electronic health... | healthcare/EHR/clinical records | United States | detection/identification; screening; risk assessment/stratification; prediction/forecasting | intimate partner violence; domestic violence/abuse | indirect IPV/domestic-abuse risk-pathway relevance | qualitative study | qualitative/exploratory evidence | risk of false reassurance/false positives requires human review | Links intimate partner violence; domestic violence/abuse with detection/identification; screening; risk assessment/stratification; prediction/forecasting through deep learning... | Coded from available bibliographic/charting information; not reported/unclear means not inferable from the available record-level charting. |
| 33 | Li et al. (2026) | artificial intelligence; machine learning; deep learning; NLP/text mining; LLM/generative AI; predictive... | healthcare/EHR/clinical records | United States | detection/identification; screening; risk assessment/stratification; prediction/forecasting; prevention/intervention/referral | intimate partner violence | indirect IPV/domestic-abuse risk-pathway relevance | review | review evidence | governance/ethics explicitly indicated in charting; risk of false reassurance/false positives requires human review | Links intimate partner violence with detection/identification; screening; risk assessment/stratification; prediction/forecasting; prevention/intervention/referral; legal/ethical... | Coded from available bibliographic/charting information; not reported/unclear means not inferable from the available record-level charting. |
| 34 | D. D. Awasekar et al. (2025) | artificial intelligence; machine learning; predictive analytics/modeling; decision support/risk stratification | police/legal/judicial or mortality records | India | prediction/forecasting; decision support/triage; prevention/intervention/referral | domestic violence/abuse | indirect IPV/domestic-abuse risk-pathway relevance | model development/validation | model development/evaluation/validation | risk of false reassurance/false positives requires human review | Links domestic violence/abuse with prediction/forecasting; decision support/triage; prevention/intervention/referral through artificial intelligence; machine learning; predictive... | Coded from available bibliographic/charting information; not reported/unclear means not inferable from the available record-level charting. |
| 35 | Guan et al. (2025) | artificial intelligence; NLP/text mining; LLM/generative AI; predictive analytics/modeling | healthcare/EHR/clinical records; online/social media/digital platform | United States; China | detection/identification; prediction/forecasting; prevention/intervention/referral | intimate partner violence; domestic violence/abuse | indirect IPV/domestic-abuse risk-pathway relevance | model development/validation | model development/evaluation/validation | risk of false reassurance/false positives requires human review | Links intimate partner violence; domestic violence/abuse with detection/identification; prediction/forecasting; prevention/intervention/referral through artificial intelligence... | Coded from available bibliographic/charting information; not reported/unclear means not inferable from the available record-level charting. |
| 36 | Kaikhosrovi et al. (2025) | artificial intelligence; data mining; record/data linkage | healthcare/EHR/clinical records; linked administrative data | Australia; South Wales | detection/identification; screening | intimate partner violence; domestic violence/abuse; family violence; gender-based... | indirect IPV/domestic-abuse risk-pathway relevance | observational/data-linkage study | observational/data-linkage evidence | not reported/unclear from available charting | Links intimate partner violence; domestic violence/abuse; family violence; gender-based violence/violence against women with detection/identification; screening... | Coded from available bibliographic/charting information; not reported/unclear means not inferable from the available record-level charting. |
| 37 | Özsezer et al. (2026) | machine learning; predictive analytics/modeling; geospatial/search/speech/computer vision | online/social media/digital platform | Turkey | detection/identification; risk assessment/stratification; early warning/surveillance; prediction/forecasting | intimate partner violence; domestic violence/abuse | indirect IPV/domestic-abuse risk-pathway relevance | model development/validation | model development/evaluation/validation | risk of false reassurance/false positives requires human review | Links intimate partner violence; domestic violence/abuse with detection/identification; risk assessment/stratification; prediction/forecasting; early warning/surveillance... | Coded from available bibliographic/charting information; not reported/unclear means not inferable from the available record-level charting. |
| 38 | F. Mousavi et al. (2025) | artificial intelligence; machine learning; deep learning; NLP/text mining; predictive analytics/modeling... | online/social media/digital platform | United Kingdom | detection/identification; prediction/forecasting | intimate partner violence; domestic violence/abuse; gender-based violence/violence against women | indirect IPV/domestic-abuse risk-pathway relevance | model development/validation | model development/evaluation/validation | risk of false reassurance/false positives requires human review | Links intimate partner violence; domestic violence/abuse; gender-based violence/violence against women with detection/identification; prediction/forecasting... | Coded from available bibliographic/charting information; not reported/unclear means not inferable from the available record-level charting. |
| 39 | Saudagar S. et al. (2025) | artificial intelligence; machine learning; deep learning; NLP/text mining; predictive analytics/modeling | not reported/unclear from available charting | India; Bangladesh; Liberia | detection/identification | intimate partner violence; domestic violence/abuse; family violence; gender-based... | indirect IPV/domestic-abuse risk-pathway relevance | model development/validation | model development/evaluation/validation | risk of false reassurance/false positives requires human review | Links intimate partner violence; domestic violence/abuse; family violence; gender-based violence/violence against women with detection/identification; risk... | Coded from available bibliographic/charting information; not reported/unclear means not inferable from the available record-level charting. |
| 40 | A. F. A. H. Alnuaimi et al. (2025) | machine learning; predictive analytics/modeling; data mining | police/legal/judicial or mortality records; online/social media/digital platform | Iraq | detection/identification; prediction/forecasting; prevention/intervention/referral | domestic violence/abuse | indirect IPV/domestic-abuse risk-pathway relevance | model development/validation | model development/evaluation/validation | risk of false reassurance/false positives requires human review | Links domestic violence/abuse with detection/identification; prediction/forecasting; prevention/intervention/referral through machine learning; predictive analytics/modeling... | Coded from available bibliographic/charting information; not reported/unclear means not inferable from the available record-level charting. |
| 41 | Cruz-Mendoza M.C. et al. (2025) | machine learning; deep learning; geospatial/search/speech/computer vision | online/social media/digital platform | United States; India; Mexico | detection/identification; prediction/forecasting | intimate partner violence; intimate partner homicide/domestic homicide; gender-based... | direct femicide/homicide/lethality or severe-escalation relevance | model development/validation | model development/evaluation/validation | risk of false reassurance/false positives requires human review | Links intimate partner violence; intimate partner homicide/domestic homicide; gender-based violence/violence against women with detection/identification; prediction/forecasting... | Coded from available bibliographic/charting information; not reported/unclear means not inferable from the available record-level charting. |
| 42 | Warnars H.L.H.S. et al. (2025) | artificial intelligence; machine learning; predictive analytics/modeling | not reported/unclear from available charting | United Kingdom | detection/identification; prediction/forecasting; prevention/intervention/referral | domestic violence/abuse; gender-based violence/violence against women | indirect IPV/domestic-abuse risk-pathway relevance | model development/validation | model development/evaluation/validation | risk of false reassurance/false positives requires human review | Links domestic violence/abuse; gender-based violence/violence against women with detection/identification; prediction/forecasting; prevention/intervention/referral through... | Coded from available bibliographic/charting information; not reported/unclear means not inferable from the available record-level charting. |
| 43 | González-Sanabria J.-S. et al. (2025) | machine learning | not reported/unclear from available charting | Spain; Colombia | detection/identification; early warning/surveillance; prediction/forecasting | femicide/feminicide; gender-based violence/violence against women | direct femicide/homicide/lethality or severe-escalation relevance | model development/validation | model development/evaluation/validation | risk of false reassurance/false positives requires human review | Links femicide/feminicide; gender-based violence/violence against women with detection/identification; prediction/forecasting; early warning/surveillance... | Coded from available bibliographic/charting information; not reported/unclear means not inferable from the available record-level charting. |
| 44 | Shashidhara et al. (2024) | machine learning; predictive analytics/modeling | not reported/unclear from available charting | United States; Spain; Australia | detection/identification; risk assessment/stratification; prediction/forecasting; legal/ethical governance | intimate partner violence; domestic violence/abuse | indirect IPV/domestic-abuse risk-pathway relevance | review | review evidence | governance/ethics explicitly indicated in charting; risk of false reassurance/false positives requires human review | Links intimate partner violence; domestic violence/abuse with detection/identification; risk assessment/stratification; prediction/forecasting; legal/ethical governance through... | Coded from available bibliographic/charting information; not reported/unclear means not inferable from the available record-level charting. |
| 45 | Aguilar et al. (2023) | artificial intelligence; deep learning; geospatial/search/speech/computer vision | not reported/unclear from available charting | not reported | detection/identification; prevention/intervention/referral | domestic violence/abuse; gender-based violence/violence against women | indirect IPV/domestic-abuse risk-pathway relevance | model development/validation | model development/evaluation/validation | not reported/unclear from available charting | Links domestic violence/abuse; gender-based violence/violence against women with detection/identification; prevention/intervention/referral through artificial intelligence; deep... | Coded from available bibliographic/charting information; not reported/unclear means not inferable from the available record-level charting. |
| 46 | Jung et al. (2022) | predictive analytics/modeling | not reported/unclear from available charting | Canada | detection/identification; risk assessment/stratification | intimate partner violence; coercive control; intimate partner homicide/domestic homicide; stalking | direct femicide/homicide/lethality or severe-escalation relevance | model development/validation | model development/evaluation/validation | risk of false reassurance/false positives requires human review | Links intimate partner violence; coercive control; intimate partner homicide/domestic homicide; stalking with detection/identification; risk assessment/stratification... | Coded from available bibliographic/charting information; not reported/unclear means not inferable from the available record-level charting. |
| 47 | Ludwig (2022) | machine learning; predictive analytics/modeling; record/data linkage | linked administrative data | not reported | detection/identification; prediction/forecasting | intimate partner violence; domestic violence/abuse; gender-based violence/violence against women | indirect IPV/domestic-abuse risk-pathway relevance | clinical trial / implementation trial | implementation or trial evidence reported | risk of false reassurance/false positives requires human review | Links intimate partner violence; domestic violence/abuse; gender-based violence/violence against women with detection/identification; prediction/forecasting... | Coded from available bibliographic/charting information; not reported/unclear means not inferable from the available record-level charting. |
| 48 | Hossain M.M. et al. (2021) | machine learning | online/social media/digital platform | Bangladesh; Italy; China | prediction/forecasting; prevention/intervention/referral | domestic violence/abuse; family violence; gender-based violence/violence against women | indirect IPV/domestic-abuse risk-pathway relevance | model development/validation | model development/evaluation/validation | risk of false reassurance/false positives requires human review | Links domestic violence/abuse; family violence; gender-based violence/violence against women with prediction/forecasting; prevention/intervention/referral through machine learning. | Coded from available bibliographic/charting information; not reported/unclear means not inferable from the available record-level charting. |
| 49 | Choi et al. (2016) | predictive analytics/modeling | not reported/unclear from available charting | Korea; South Korea | prediction/forecasting; prevention/intervention/referral | domestic violence/abuse; family violence | indirect IPV/domestic-abuse risk-pathway relevance | model development/validation | model development/evaluation/validation | risk of false reassurance/false positives requires human review | Links domestic violence/abuse; family violence with prediction/forecasting; prevention/intervention/referral through predictive analytics/modeling. | Coded from available bibliographic/charting information; not reported/unclear means not inferable from the available record-level charting. |
| 50 | Halpern et al. (2006) | predictive analytics/modeling | healthcare/EHR/clinical records | not reported | detection/identification; screening; risk assessment/stratification; prediction/forecasting; prevention/intervention/referral | intimate partner violence | indirect IPV/domestic-abuse risk-pathway relevance | clinical trial / implementation trial | implementation or trial evidence reported | risk of false reassurance/false positives requires human review | Links intimate partner violence with detection/identification; screening; risk assessment/stratification; prediction/forecasting; prevention/intervention/referral through... | Coded from available bibliographic/charting information; not reported/unclear means not inferable from the available record-level charting. |
| 51 | Sprecher et al. (2004) | deep learning | healthcare/EHR/clinical records | not reported | detection/identification; screening | intimate partner violence; domestic violence/abuse | indirect IPV/domestic-abuse risk-pathway relevance | model development/validation | model development/evaluation/validation | not reported/unclear from available charting | Links intimate partner violence; domestic violence/abuse with detection/identification; screening through deep learning. | Coded from available bibliographic/charting information; not reported/unclear means not inferable from the available record-level charting. |
| 52 | Barboza-Salerno et al. (2026) | NLP/text mining | healthcare/EHR/clinical records | United States | detection/identification; screening; early warning/surveillance; prediction/forecasting; prevention/intervention/referral | intimate partner violence | indirect IPV/domestic-abuse risk-pathway relevance | review | review evidence | governance/ethics explicitly indicated in charting; risk of false reassurance/false positives requires human review | Links intimate partner violence with detection/identification; screening; prediction/forecasting; early warning/surveillance; prevention/intervention/referral; legal/ethical... | Coded from available bibliographic/charting information; not reported/unclear means not inferable from the available record-level charting. |
| 53 | Turner et al. (2022) | machine learning; predictive analytics/modeling | police/legal/judicial or mortality records | Spain; United Kingdom | detection/identification; risk assessment/stratification; prediction/forecasting | intimate partner violence; domestic violence/abuse; stalking | indirect IPV/domestic-abuse risk-pathway relevance | model development/validation | model development/evaluation/validation | risk of false reassurance/false positives requires human review | Links intimate partner violence; domestic violence/abuse; stalking with detection/identification; risk assessment/stratification; prediction/forecasting... | Coded from available bibliographic/charting information; not reported/unclear means not inferable from the available record-level charting. |
| 54 | Warnars H.L.H.S. et al. (2026) | machine learning; predictive analytics/modeling; geospatial/search/speech/computer vision | not reported/unclear from available charting | United Kingdom | detection/identification; risk assessment/stratification; early warning/surveillance; prediction/forecasting | gender-based violence/violence against women | indirect IPV/domestic-abuse risk-pathway relevance | model development/validation | model development/evaluation/validation | risk of false reassurance/false positives requires human review | Links gender-based violence/violence against women with detection/identification; risk assessment/stratification; prediction/forecasting; early warning/surveillance... | Coded from available bibliographic/charting information; not reported/unclear means not inferable from the available record-level charting. |
| 55 | Başaran et al. (2025) | artificial intelligence; machine learning; predictive analytics/modeling | healthcare/EHR/clinical records | Turkey | detection/identification; risk assessment/stratification | intimate partner violence; domestic violence/abuse; gender-based violence/violence against women... | indirect IPV/domestic-abuse risk-pathway relevance | model development/validation | model development/evaluation/validation | risk of false reassurance/false positives requires human review | Links intimate partner violence; domestic violence/abuse; gender-based violence/violence against women; stalking with detection/identification; risk assessment/stratification... | Coded from available bibliographic/charting information; not reported/unclear means not inferable from the available record-level charting. |
| 56 | Cook et al. (2025) | machine learning; deep learning; NLP/text mining | police/legal/judicial or mortality records | United Kingdom | detection/identification; risk assessment/stratification; prediction/forecasting; prevention/intervention/referral | domestic violence/abuse | indirect IPV/domestic-abuse risk-pathway relevance | model development/validation | model development/evaluation/validation | risk of false reassurance/false positives requires human review | Links domestic violence/abuse with detection/identification; risk assessment/stratification; prediction/forecasting; prevention/intervention/referral through machine learning... | Coded from available bibliographic/charting information; not reported/unclear means not inferable from the available record-level charting. |
| 57 | Kurniawan T.B. et al. (2025) | artificial intelligence; machine learning; deep learning; geospatial/search/speech/computer vision | online/social media/digital platform | not reported | detection/identification | gender-based violence/violence against women | indirect IPV/domestic-abuse risk-pathway relevance | model development/validation | model development/evaluation/validation | not reported/unclear from available charting | Links gender-based violence/violence against women with detection/identification through artificial intelligence; machine learning; deep learning... | Coded from available bibliographic/charting information; not reported/unclear means not inferable from the available record-level charting. |
| 58 | Alnuaimi A.F.A.H. et al. (2025) | machine learning; deep learning; predictive analytics/modeling | police/legal/judicial or mortality records | Iraq | detection/identification; prediction/forecasting; prevention/intervention/referral | domestic violence/abuse | indirect IPV/domestic-abuse risk-pathway relevance | model development/validation | model development/evaluation/validation | risk of false reassurance/false positives requires human review | Links domestic violence/abuse with detection/identification; prediction/forecasting; prevention/intervention/referral through machine learning; deep learning; predictive... | Coded from available bibliographic/charting information; not reported/unclear means not inferable from the available record-level charting. |
| 59 | Hadjimatheou K. et al. (2024) | machine learning; geospatial/search/speech/computer vision | not reported/unclear from available charting | United Kingdom | detection/identification; prevention/intervention/referral | domestic violence/abuse | indirect IPV/domestic-abuse risk-pathway relevance | qualitative study | qualitative/exploratory evidence | not reported/unclear from available charting | Links domestic violence/abuse with detection/identification; prevention/intervention/referral through machine learning; geospatial/search/speech/computer vision. | Coded from available bibliographic/charting information; not reported/unclear means not inferable from the available record-level charting. |
| 60 | De Filippo et al. (2023) | chatbot/digital assistant | online/social media/digital platform | United States; United Kingdom; South Africa | detection/identification; screening; prevention/intervention/referral | intimate partner violence | indirect IPV/domestic-abuse risk-pathway relevance | clinical trial / implementation trial | implementation or trial evidence reported | not reported/unclear from available charting | Links intimate partner violence with detection/identification; screening; prevention/intervention/referral through chatbot/digital assistant. | Coded from available bibliographic/charting information; not reported/unclear means not inferable from the available record-level charting. |
| 61 | Poelmans J. et al. (2011) | NLP/text mining; data mining | online/social media/digital platform | Netherlands | detection/identification; decision support/triage | domestic violence/abuse | indirect IPV/domestic-abuse risk-pathway relevance | model development/validation | model development/evaluation/validation | risk of false reassurance/false positives requires human review | Links domestic violence/abuse with detection/identification; decision support/triage through NLP/text mining; data mining. | Coded from available bibliographic/charting information; not reported/unclear means not inferable from the available record-level charting. |
| 62 | Thompson Lee et al. (2026) | artificial intelligence; machine learning; deep learning; data mining; geospatial/search/speech/computer... | not reported/unclear from available charting | United States | detection/identification; prediction/forecasting; prevention/intervention/referral | domestic violence/abuse; gender-based violence/violence against women | indirect IPV/domestic-abuse risk-pathway relevance | qualitative study | qualitative/exploratory evidence | risk of false reassurance/false positives requires human review | Links domestic violence/abuse; gender-based violence/violence against women with detection/identification; prediction/forecasting; prevention/intervention/referral through... | Coded from available bibliographic/charting information; not reported/unclear means not inferable from the available record-level charting. |
| 63 | Vogt et al. (2026) | artificial intelligence; chatbot/digital assistant | online/social media/digital platform | Spain | detection/identification | domestic violence/abuse; intimate partner homicide/domestic homicide; gender-based... | direct femicide/homicide/lethality or severe-escalation relevance | qualitative study | qualitative/exploratory evidence | risk of false reassurance/false positives requires human review | Links domestic violence/abuse; intimate partner homicide/domestic homicide; gender-based violence/violence against women with detection/identification; risk... | Coded from available bibliographic/charting information; not reported/unclear means not inferable from the available record-level charting. |
| 64 | Awasekar D.D. et al. (2025) | artificial intelligence; machine learning; NLP/text mining; chatbot/digital assistant | police/legal/judicial or mortality records; online/social media/digital platform | India | prevention/intervention/referral; legal/ethical governance | domestic violence/abuse | indirect IPV/domestic-abuse risk-pathway relevance | model development/validation | model development/evaluation/validation | governance/ethics explicitly indicated in charting | Links domestic violence/abuse with prevention/intervention/referral; legal/ethical governance through artificial intelligence; machine learning; NLP/text mining; chatbot/digital... | Coded from available bibliographic/charting information; not reported/unclear means not inferable from the available record-level charting. |
| 65 | Ogğuztüzün et al. (2024) | machine learning; predictive analytics/modeling | not reported/unclear from available charting | United States | detection/identification; risk assessment/stratification; prediction/forecasting; prevention/intervention/referral | intimate partner violence; domestic violence/abuse | indirect IPV/domestic-abuse risk-pathway relevance | qualitative study | qualitative/exploratory evidence | risk of false reassurance/false positives requires human review | Links intimate partner violence; domestic violence/abuse with detection/identification; risk assessment/stratification; prediction/forecasting; prevention/intervention/referral... | Coded from available bibliographic/charting information; not reported/unclear means not inferable from the available record-level charting. |
| 66 | Escobar-Linero et al. (2023) | artificial intelligence; machine learning; predictive analytics/modeling | police/legal/judicial or mortality records | Spain; India | detection/identification; prediction/forecasting; prevention/intervention/referral | intimate partner violence; gender-based violence/violence against women | indirect IPV/domestic-abuse risk-pathway relevance | model development/validation | model development/evaluation/validation | risk of false reassurance/false positives requires human review | Links intimate partner violence; gender-based violence/violence against women with detection/identification; prediction/forecasting; prevention/intervention/referral through... | Coded from available bibliographic/charting information; not reported/unclear means not inferable from the available record-level charting. |
| 67 | Berk R.A. et al. (2016) | machine learning; predictive analytics/modeling | police/legal/judicial or mortality records; online/social media/digital platform | not reported | prediction/forecasting; decision support/triage; prevention/intervention/referral | domestic violence/abuse | indirect IPV/domestic-abuse risk-pathway relevance | clinical trial / implementation trial | implementation or trial evidence reported | risk of false reassurance/false positives requires human review | Links domestic violence/abuse with prediction/forecasting; decision support/triage; prevention/intervention/referral through machine learning; predictive analytics/modeling. | Coded from available bibliographic/charting information; not reported/unclear means not inferable from the available record-level charting. |
| 68 | Um et al. (2025) | machine learning; predictive analytics/modeling | not reported/unclear from available charting | United States | detection/identification; prediction/forecasting; prevention/intervention/referral | intimate partner violence; stalking | indirect IPV/domestic-abuse risk-pathway relevance | model development/validation | model development/evaluation/validation | risk of false reassurance/false positives requires human review | Links intimate partner violence; stalking with detection/identification; prediction/forecasting; prevention/intervention/referral through machine learning; predictive... | Coded from available bibliographic/charting information; not reported/unclear means not inferable from the available record-level charting. |
| 69 | Awasekar D. et al. (2025) | artificial intelligence; chatbot/digital assistant; geospatial/search/speech/computer vision | police/legal/judicial or mortality records; online/social media/digital platform | United States; India; Turkey | detection/identification; prevention/intervention/referral | domestic violence/abuse | indirect IPV/domestic-abuse risk-pathway relevance | qualitative study | qualitative/exploratory evidence | not reported/unclear from available charting | Links domestic violence/abuse with detection/identification; prevention/intervention/referral through artificial intelligence; chatbot/digital assistant... | Coded from available bibliographic/charting information; not reported/unclear means not inferable from the available record-level charting. |
| 70 | Hui et al. (2026) | artificial intelligence; chatbot/digital assistant | online/social media/digital platform | United States | detection/identification; prevention/intervention/referral; legal/ethical governance | intimate partner violence; domestic violence/abuse; family violence | indirect IPV/domestic-abuse risk-pathway relevance | qualitative study | qualitative/exploratory evidence | governance/ethics explicitly indicated in charting | Links intimate partner violence; domestic violence/abuse; family violence with detection/identification; prevention/intervention/referral; legal/ethical governance through... | Coded from available bibliographic/charting information; not reported/unclear means not inferable from the available record-level charting. |
| 71 | Verma P. et al. (2026) | machine learning; deep learning; predictive analytics/modeling | online/social media/digital platform | India | detection/identification; prediction/forecasting | intimate partner violence; domestic violence/abuse; gender-based violence/violence against women | indirect IPV/domestic-abuse risk-pathway relevance | model development/validation | model development/evaluation/validation | risk of false reassurance/false positives requires human review | Links intimate partner violence; domestic violence/abuse; gender-based violence/violence against women with detection/identification; prediction/forecasting... | Coded from available bibliographic/charting information; not reported/unclear means not inferable from the available record-level charting. |
| 72 | Hernandez-Zetina S. et al. (2026) | machine learning; deep learning; predictive analytics/modeling; geospatial/search/speech/computer vision | not reported/unclear from available charting | Spain; Mexico | detection/identification; prediction/forecasting; prevention/intervention/referral | gender-based violence/violence against women | indirect IPV/domestic-abuse risk-pathway relevance | model development/validation | model development/evaluation/validation | risk of false reassurance/false positives requires human review | Links gender-based violence/violence against women with detection/identification; prediction/forecasting; prevention/intervention/referral through machine learning; deep... | Coded from available bibliographic/charting information; not reported/unclear means not inferable from the available record-level charting. |
| 73 | Giorgio A. (2026) | artificial intelligence; geospatial/search/speech/computer vision | online/social media/digital platform | Italy | detection/identification; early warning/surveillance; prevention/intervention/referral | femicide/feminicide; gender-based violence/violence against women | direct femicide/homicide/lethality or severe-escalation relevance | not reported/unclear | not reported/unclear or conceptual/technical | not reported/unclear from available charting | Links femicide/feminicide; gender-based violence/violence against women with detection/identification; early warning/surveillance; prevention/intervention/referral through... | Coded from available bibliographic/charting information; not reported/unclear means not inferable from the available record-level charting. |
| 74 | Hui et al. (2025) | deep learning; NLP/text mining; LLM/generative AI | online/social media/digital platform | United States | detection/identification; prevention/intervention/referral | domestic violence/abuse | indirect IPV/domestic-abuse risk-pathway relevance | clinical trial / implementation trial | implementation or trial evidence reported | not reported/unclear from available charting | Links domestic violence/abuse with detection/identification; prevention/intervention/referral through deep learning; NLP/text mining; LLM/generative AI. | Coded from available bibliographic/charting information; not reported/unclear means not inferable from the available record-level charting. |
| 75 | Zhang et al. (2025) | machine learning; deep learning; NLP/text mining; geospatial/search/speech/computer vision | online/social media/digital platform | China | detection/identification; prevention/intervention/referral | domestic violence/abuse | indirect IPV/domestic-abuse risk-pathway relevance | model development/validation | model development/evaluation/validation | not reported/unclear from available charting | Links domestic violence/abuse with detection/identification; prevention/intervention/referral through machine learning; deep learning; NLP/text mining... | Coded from available bibliographic/charting information; not reported/unclear means not inferable from the available record-level charting. |
| 76 | Talwalk et al. (2025) | artificial intelligence; predictive analytics/modeling | not reported/unclear from available charting | United States; Canada | detection/identification; prediction/forecasting; prevention/intervention/referral | intimate partner violence; domestic violence/abuse; coercive control | indirect IPV/domestic-abuse risk-pathway relevance | model development/validation | model development/evaluation/validation | governance/ethics explicitly indicated in charting; risk of false reassurance/false positives requires human review | Links intimate partner violence; domestic violence/abuse; coercive control with detection/identification; prediction/forecasting; prevention/intervention/referral; legal/ethical... | Coded from available bibliographic/charting information; not reported/unclear means not inferable from the available record-level charting. |
| 77 | Gonzálvez-Gallego N. et al. (2024) | predictive analytics/modeling; geospatial/search/speech/computer vision | not reported/unclear from available charting | United States; Spain | detection/identification | intimate partner violence; domestic violence/abuse; femicide/feminicide; gender-based... | direct femicide/homicide/lethality or severe-escalation relevance | model development/validation | model development/evaluation/validation | risk of false reassurance/false positives requires human review | Links intimate partner violence; domestic violence/abuse; femicide/feminicide; gender-based violence/violence against women; stalking with detection/identification; risk... | Coded from available bibliographic/charting information; not reported/unclear means not inferable from the available record-level charting. |
| 78 | Salehi et al. (2023) | machine learning; NLP/text mining; predictive analytics/modeling; geospatial/search/speech/computer vision | online/social media/digital platform | Iran | detection/identification; screening; prediction/forecasting; prevention/intervention/referral; legal/ethical governance | domestic violence/abuse; family violence | indirect IPV/domestic-abuse risk-pathway relevance | clinical trial / implementation trial | implementation or trial evidence reported | governance/ethics explicitly indicated in charting; risk of false reassurance/false positives requires human review | Links domestic violence/abuse; family violence with detection/identification; screening; prediction/forecasting; prevention/intervention/referral; legal/ethical governance... | Coded from available bibliographic/charting information; not reported/unclear means not inferable from the available record-level charting. |
| 79 | Sisodia A. et al. (2023) | machine learning; predictive analytics/modeling; geospatial/search/speech/computer vision | online/social media/digital platform | India; Canada | detection/identification; prediction/forecasting | intimate partner violence; gender-based violence/violence against women | indirect IPV/domestic-abuse risk-pathway relevance | review | review evidence | risk of false reassurance/false positives requires human review | Links intimate partner violence; gender-based violence/violence against women with detection/identification; prediction/forecasting through machine learning; predictive... | Coded from available bibliographic/charting information; not reported/unclear means not inferable from the available record-level charting. |
| 80 | Reyner-Fuentes et al. (2023) | artificial intelligence; machine learning; deep learning; geospatial/search/speech/computer vision | not reported/unclear from available charting | Spain | detection/identification; prediction/forecasting; legal/ethical governance | gender-based violence/violence against women | indirect IPV/domestic-abuse risk-pathway relevance | qualitative study | qualitative/exploratory evidence | governance/ethics explicitly indicated in charting; risk of false reassurance/false positives requires human review | Links gender-based violence/violence against women with detection/identification; prediction/forecasting; legal/ethical governance through artificial intelligence; machine... | Coded from available bibliographic/charting information; not reported/unclear means not inferable from the available record-level charting. |
| 81 | L. R. Abdulkareem et al. (2022) | artificial intelligence; machine learning; deep learning; predictive analytics/modeling; data mining... | not reported/unclear from available charting | Iraq | detection/identification; prediction/forecasting; prevention/intervention/referral | gender-based violence/violence against women | indirect IPV/domestic-abuse risk-pathway relevance | model development/validation | model development/evaluation/validation | risk of false reassurance/false positives requires human review | Links gender-based violence/violence against women with detection/identification; prediction/forecasting; prevention/intervention/referral through artificial intelligence... | Coded from available bibliographic/charting information; not reported/unclear means not inferable from the available record-level charting. |
| 82 | Victor et al. (2021) | machine learning; deep learning; NLP/text mining; predictive analytics/modeling; record/data linkage | linked administrative data | United States | detection/identification; risk assessment/stratification; prediction/forecasting; prevention/intervention/referral | domestic violence/abuse | indirect IPV/domestic-abuse risk-pathway relevance | model development/validation | model development/evaluation/validation | risk of false reassurance/false positives requires human review | Links domestic violence/abuse with detection/identification; risk assessment/stratification; prediction/forecasting; prevention/intervention/referral through machine learning... | Coded from available bibliographic/charting information; not reported/unclear means not inferable from the available record-level charting. |
| 83 | Castorena C.M. et al. (2021) | machine learning; deep learning; geospatial/search/speech/computer vision | online/social media/digital platform | Mexico | detection/identification | gender-based violence/violence against women | indirect IPV/domestic-abuse risk-pathway relevance | model development/validation | model development/evaluation/validation | not reported/unclear from available charting | Links gender-based violence/violence against women with detection/identification through machine learning; deep learning; geospatial/search/speech/computer vision. | Coded from available bibliographic/charting information; not reported/unclear means not inferable from the available record-level charting. |
| 84 | Rodríguez-Rodríguez I. et al. (2020) | machine learning; deep learning; predictive analytics/modeling | online/social media/digital platform | Spain; Italy | prediction/forecasting; prevention/intervention/referral | intimate partner violence; domestic violence/abuse; gender-based violence/violence against women | indirect IPV/domestic-abuse risk-pathway relevance | model development/validation | model development/evaluation/validation | risk of false reassurance/false positives requires human review | Links intimate partner violence; domestic violence/abuse; gender-based violence/violence against women with prediction/forecasting; prevention/intervention/referral through... | Coded from available bibliographic/charting information; not reported/unclear means not inferable from the available record-level charting. |
| 85 | Subramani et al. (2019) | deep learning; geospatial/search/speech/computer vision | online/social media/digital platform | Australia; China | detection/identification; prediction/forecasting; prevention/intervention/referral | domestic violence/abuse; gender-based violence/violence against women | indirect IPV/domestic-abuse risk-pathway relevance | model development/validation | model development/evaluation/validation | risk of false reassurance/false positives requires human review | Links domestic violence/abuse; gender-based violence/violence against women with detection/identification; prediction/forecasting; prevention/intervention/referral through deep... | Coded from available bibliographic/charting information; not reported/unclear means not inferable from the available record-level charting. |
| 86 | S. Subramani et al. (2018) | machine learning; deep learning; geospatial/search/speech/computer vision | online/social media/digital platform | Australia | detection/identification; prevention/intervention/referral | domestic violence/abuse | indirect IPV/domestic-abuse risk-pathway relevance | model development/validation | model development/evaluation/validation | not reported/unclear from available charting | Links domestic violence/abuse with detection/identification; prevention/intervention/referral through machine learning; deep learning; geospatial/search/speech/computer vision. | Coded from available bibliographic/charting information; not reported/unclear means not inferable from the available record-level charting. |
| 87 | Petering R. et al. (2018) | artificial intelligence; machine learning | not reported/unclear from available charting | United States | detection/identification; prediction/forecasting; decision support/triage; prevention/intervention/referral | intimate partner violence; domestic violence/abuse | indirect IPV/domestic-abuse risk-pathway relevance | model development/validation | model development/evaluation/validation | risk of false reassurance/false positives requires human review | Links intimate partner violence; domestic violence/abuse with detection/identification; prediction/forecasting; decision support/triage; prevention/intervention/referral through... | Coded from available bibliographic/charting information; not reported/unclear means not inferable from the available record-level charting. |
| 88 | Van Hightower et al. (2000) | deep learning; predictive analytics/modeling | not reported/unclear from available charting | not reported | screening; prediction/forecasting; prevention/intervention/referral | domestic violence/abuse; family violence | indirect IPV/domestic-abuse risk-pathway relevance | model development/validation | model development/evaluation/validation | risk of false reassurance/false positives requires human review | Links domestic violence/abuse; family violence with screening; prediction/forecasting; prevention/intervention/referral through deep learning; predictive analytics/modeling. | Coded from available bibliographic/charting information; not reported/unclear means not inferable from the available record-level charting. |
| 89 | Marisetti M. et al. (2026) | artificial intelligence; machine learning; geospatial/search/speech/computer vision | not reported/unclear from available charting | United States; Spain; Australia | detection/identification; prevention/intervention/referral; legal/ethical governance | intimate partner violence; domestic violence/abuse | indirect IPV/domestic-abuse risk-pathway relevance | model development/validation | model development/evaluation/validation | governance/ethics explicitly indicated in charting | Links intimate partner violence; domestic violence/abuse with detection/identification; prevention/intervention/referral; legal/ethical governance through artificial... | Coded from available bibliographic/charting information; not reported/unclear means not inferable from the available record-level charting. |
| 90 | Le B.L. et al. (2026) | machine learning; predictive analytics/modeling | not reported/unclear from available charting | United Kingdom | detection/identification; risk assessment/stratification | intimate partner violence; domestic violence/abuse; femicide/feminicide; lethality/severe escalation | direct femicide/homicide/lethality or severe-escalation relevance | protocol | not reported/unclear or conceptual/technical | risk of false reassurance/false positives requires human review | Links intimate partner violence; domestic violence/abuse; femicide/feminicide; lethality/severe escalation with detection/identification; risk assessment/stratification... | Coded from available bibliographic/charting information; not reported/unclear means not inferable from the available record-level charting. |
| 91 | Singh S. et al. (2026) | artificial intelligence; machine learning; deep learning | not reported/unclear from available charting | United States; Brazil; Australia | prevention/intervention/referral | domestic violence/abuse | indirect IPV/domestic-abuse risk-pathway relevance | model development/validation | model development/evaluation/validation | not reported/unclear from available charting | Links domestic violence/abuse with prevention/intervention/referral through artificial intelligence; machine learning; deep learning. | Coded from available bibliographic/charting information; not reported/unclear means not inferable from the available record-level charting. |
| 92 | Sood V. (2025) | artificial intelligence; machine learning; deep learning; predictive analytics/modeling; decision... | not reported/unclear from available charting | United States; Brazil; India | prediction/forecasting; decision support/triage; prevention/intervention/referral | domestic violence/abuse | indirect IPV/domestic-abuse risk-pathway relevance | model development/validation | model development/evaluation/validation | risk of false reassurance/false positives requires human review | Links domestic violence/abuse with prediction/forecasting; decision support/triage; prevention/intervention/referral through artificial intelligence; machine learning; deep... | Coded from available bibliographic/charting information; not reported/unclear means not inferable from the available record-level charting. |
| 93 | Hui et al. (2024) | artificial intelligence; machine learning; NLP/text mining; LLM/generative AI; predictive analytics/modeling | online/social media/digital platform | United States | detection/identification; prediction/forecasting; prevention/intervention/referral | intimate partner violence; domestic violence/abuse | indirect IPV/domestic-abuse risk-pathway relevance | model development/validation | model development/evaluation/validation | risk of false reassurance/false positives requires human review | Links intimate partner violence; domestic violence/abuse with detection/identification; prediction/forecasting; prevention/intervention/referral through artificial intelligence... | Coded from available bibliographic/charting information; not reported/unclear means not inferable from the available record-level charting. |
| 94 | Sachdeva et al. (2024) | machine learning; EHR/electronic health records | healthcare/EHR/clinical records | not reported | detection/identification; screening | intimate partner violence; domestic violence/abuse | indirect IPV/domestic-abuse risk-pathway relevance | model development/validation | model development/evaluation/validation | not reported/unclear from available charting | Links intimate partner violence; domestic violence/abuse with detection/identification; screening through machine learning; EHR/electronic health records. | Coded from available bibliographic/charting information; not reported/unclear means not inferable from the available record-level charting. |
| 95 | Szyfer Lipinsky et al. (2024) | machine learning; predictive analytics/modeling | online/social media/digital platform | Israel | detection/identification; risk assessment/stratification; prediction/forecasting; prevention/intervention/referral | intimate partner violence | indirect IPV/domestic-abuse risk-pathway relevance | qualitative study | qualitative/exploratory evidence | risk of false reassurance/false positives requires human review | Links intimate partner violence with detection/identification; risk assessment/stratification; prediction/forecasting; prevention/intervention/referral through machine learning... | Coded from available bibliographic/charting information; not reported/unclear means not inferable from the available record-level charting. |
| 96 | Contreras-Jiménez et al. (2024) | machine learning; predictive analytics/modeling; data mining; geospatial/search/speech/computer vision | online/social media/digital platform | United States; Mexico; South Africa | detection/identification; prediction/forecasting; prevention/intervention/referral | gender-based violence/violence against women | indirect IPV/domestic-abuse risk-pathway relevance | model development/validation | model development/evaluation/validation | risk of false reassurance/false positives requires human review | Links gender-based violence/violence against women with detection/identification; prediction/forecasting; prevention/intervention/referral through machine learning; predictive... | Coded from available bibliographic/charting information; not reported/unclear means not inferable from the available record-level charting. |
| 97 | Rahman et al. (2023) | machine learning; predictive analytics/modeling | not reported/unclear from available charting | Bangladesh; Liberia | detection/identification; risk assessment/stratification | intimate partner violence; domestic violence/abuse; gender-based violence/violence against women | indirect IPV/domestic-abuse risk-pathway relevance | model development/validation | model development/evaluation/validation | risk of false reassurance/false positives requires human review | Links intimate partner violence; domestic violence/abuse; gender-based violence/violence against women with detection/identification; risk assessment/stratification... | Coded from available bibliographic/charting information; not reported/unclear means not inferable from the available record-level charting. |
| 98 | Chen et al. (2023) | machine learning | survey/population or administrative data | China | prediction/forecasting; prevention/intervention/referral; legal/ethical governance | intimate partner violence; domestic violence/abuse | indirect IPV/domestic-abuse risk-pathway relevance | qualitative study | qualitative/exploratory evidence | governance/ethics explicitly indicated in charting; risk of false reassurance/false positives requires human review | Links intimate partner violence; domestic violence/abuse with prediction/forecasting; prevention/intervention/referral; legal/ethical governance through machine learning. | Coded from available bibliographic/charting information; not reported/unclear means not inferable from the available record-level charting. |
| 99 | Todorovic et al. (2022) | machine learning | online/social media/digital platform; survey/population or administrative data | United States; Netherlands | detection/identification; prediction/forecasting | intimate partner violence; family violence | indirect IPV/domestic-abuse risk-pathway relevance | model development/validation | model development/evaluation/validation | risk of false reassurance/false positives requires human review | Links intimate partner violence; family violence with detection/identification; prediction/forecasting through machine learning. | Coded from available bibliographic/charting information; not reported/unclear means not inferable from the available record-level charting. |
| 100 | Kennedy et al. (2023) | record/data linkage | police/legal/judicial or mortality records; linked administrative data | United Kingdom; South Wales | detection/identification; risk assessment/stratification; prevention/intervention/referral; inter-agency coordination | domestic violence/abuse; stalking | indirect IPV/domestic-abuse risk-pathway relevance | model development/validation | model development/evaluation/validation | governance/ethics explicitly indicated in charting; risk of false reassurance/false positives requires human review | Links domestic violence/abuse; stalking with detection/identification; risk assessment/stratification; prevention/intervention/referral; inter-agency coordination; legal/ethical... | Coded from available bibliographic/charting information; not reported/unclear means not inferable from the available record-level charting. |
| 101 | Tabaie et al. (2022) | deep learning; NLP/text mining; EHR/electronic health records | healthcare/EHR/clinical records | not reported | detection/identification; screening; prevention/intervention/referral | intimate partner violence | indirect IPV/domestic-abuse risk-pathway relevance | model development/validation | model development/evaluation/validation | not reported/unclear from available charting | Links intimate partner violence with detection/identification; screening; prevention/intervention/referral through deep learning; NLP/text mining; EHR/electronic health records. | Coded from available bibliographic/charting information; not reported/unclear means not inferable from the available record-level charting. |
| 102 | D. D. Awasekar et al. (2025) | artificial intelligence; NLP/text mining; LLM/generative AI | police/legal/judicial or mortality records | India | detection/identification; prediction/forecasting; prevention/intervention/referral | domestic violence/abuse | indirect IPV/domestic-abuse risk-pathway relevance | model development/validation | model development/evaluation/validation | risk of false reassurance/false positives requires human review | Links domestic violence/abuse with detection/identification; prediction/forecasting; prevention/intervention/referral through artificial intelligence; NLP/text mining... | Coded from available bibliographic/charting information; not reported/unclear means not inferable from the available record-level charting. |
| 103 | Botelle et al. (2022) | artificial intelligence; deep learning; NLP/text mining; data mining; EHR/electronic health records | healthcare/EHR/clinical records; online/social media/digital platform | not reported | detection/identification | domestic violence/abuse | indirect IPV/domestic-abuse risk-pathway relevance | model development/validation | model development/evaluation/validation | not reported/unclear from available charting | Links domestic violence/abuse with detection/identification through artificial intelligence; deep learning; NLP/text mining; data mining; EHR/electronic health records. | Coded from available bibliographic/charting information; not reported/unclear means not inferable from the available record-level charting. |
| 104 | González et al. (2021) | AI/algorithmic tool | online/social media/digital platform | Mexico | detection/identification | family violence; homicide/lethality; violence against women | direct femicide/homicide/lethality or severe-escalation relevance | model development/evaluation | model development/evaluation/validation | not reported/unclear from available charting | Links family violence; homicide/lethality; violence against women with detection/identification through AI/algorithmic tool. | Coded from available bibliographic/charting information; not reported/unclear means not inferable from the available record-level charting. |
| 105 | V. Ezhumalai et al. (2025) | NLP; machine learning; predictive analytics/risk prediction | online/social media/digital platform | India | detection/identification; risk assessment/stratification | domestic violence/abuse; harassment | indirect IPV/domestic-abuse risk-pathway relevance | model development/evaluation | model development/evaluation/validation | risk of false reassurance/false positives requires human review | Links domestic violence/abuse; harassment with detection/identification; risk assessment/stratification; prediction; early warning; decision support/assistance; prevention... | Coded from available bibliographic/charting information; not reported/unclear means not inferable from the available record-level charting. |
| 106 | S. Talwalkar et al. (2023) | machine learning; data mining | not reported/unclear from available charting | United States | detection/identification; risk assessment/stratification | IPV; domestic violence/abuse | indirect IPV/domestic-abuse risk-pathway relevance | model development/evaluation | model development/evaluation/validation | risk of false reassurance/false positives requires human review | Links IPV; domestic violence/abuse with detection/identification; risk assessment/stratification; prediction; decision support/assistance; prevention through machine learning... | Coded from available bibliographic/charting information; not reported/unclear means not inferable from the available record-level charting. |
| 107 | Fathima K.S. et al. (2023) | NLP; machine learning | not reported/unclear from available charting | India | detection/identification | domestic violence/abuse | indirect IPV/domestic-abuse risk-pathway relevance | survey / cross-sectional | not reported/unclear or conceptual/technical | risk of false reassurance/false positives requires human review | Links domestic violence/abuse with detection/identification; decision support/assistance; prevention through NLP; machine learning. | Coded from available bibliographic/charting information; not reported/unclear means not inferable from the available record-level charting. |
| 108 | Ngũnjiri et al. (2023) | NLP; chatbot/virtual assistant | online/social media/digital platform | Kenya; United States | risk assessment/stratification | IPV; homicide/lethality; GBV | direct femicide/homicide/lethality or severe-escalation relevance | conceptual/technical paper | not reported/unclear or conceptual/technical | risk of false reassurance/false positives requires human review | Links IPV; homicide/lethality; GBV with risk assessment/stratification; decision support/assistance; prevention through NLP; chatbot/virtual assistant. | Coded from available bibliographic/charting information; not reported/unclear means not inferable from the available record-level charting. |
| 109 | A. K. Tiwari et al. (2024) | NLP; machine learning; predictive analytics/risk prediction; chatbot/virtual assistant | online/social media/digital platform | India; United States | detection/identification; risk assessment/stratification | domestic violence/abuse; violence against women | indirect IPV/domestic-abuse risk-pathway relevance | model development/evaluation | model development/evaluation/validation | risk of false reassurance/false positives requires human review | Links domestic violence/abuse; violence against women with detection/identification; risk assessment/stratification; prediction; early warning; decision support/assistance... | Coded from available bibliographic/charting information; not reported/unclear means not inferable from the available record-level charting. |
| 110 | P. P. Shifidi et al. (2023) | machine learning | not reported/unclear from available charting | Namibia | detection/identification; risk assessment/stratification | IPV; femicide; GBV | direct femicide/homicide/lethality or severe-escalation relevance | case study / model development | model development/evaluation/validation | risk of false reassurance/false positives requires human review | Links IPV; femicide; GBV with detection/identification; risk assessment/stratification; prediction; decision support/assistance; prevention through machine learning. | Coded from available bibliographic/charting information; not reported/unclear means not inferable from the available record-level charting. |
| 111 | R. Roy et al. (2023) | machine learning; chatbot/virtual assistant; EHR-based identification | healthcare/EHR/clinical records; online/social media/digital platform | India | detection/identification; risk assessment/stratification | violence against women; harassment | indirect IPV/domestic-abuse risk-pathway relevance | review / meta-analysis | review evidence | risk of false reassurance/false positives requires human review | Links violence against women; harassment with detection/identification; risk assessment/stratification; prediction; prevention through machine learning; chatbot/virtual... | Coded from available bibliographic/charting information; not reported/unclear means not inferable from the available record-level charting. |
| 112 | Soldevilla et al. (2021) | NLP; machine learning | not reported/unclear from available charting | Peru | detection/identification | domestic violence/abuse; GBV; violence against women; harassment | indirect IPV/domestic-abuse risk-pathway relevance | survey / cross-sectional | not reported/unclear or conceptual/technical | risk of false reassurance/false positives requires human review | Links domestic violence/abuse; GBV; violence against women; harassment with detection/identification; classification; prediction; decision support/assistance through NLP; machine... | Coded from available bibliographic/charting information; not reported/unclear means not inferable from the available record-level charting. |
| 113 | V. Itham et al. (2024) | NLP; deep learning; machine learning; text mining/topic modelling | not reported/unclear from available charting | India | detection/identification | IPV; domestic violence/abuse | indirect IPV/domestic-abuse risk-pathway relevance | model development/evaluation | model development/evaluation/validation | not reported/unclear from available charting | Links IPV; domestic violence/abuse with detection/identification; classification; prevention through NLP; deep learning; machine learning; text mining/topic modelling. | Coded from available bibliographic/charting information; not reported/unclear means not inferable from the available record-level charting. |
| 114 | Stephanie E.M.A. et al. (2024) | NLP; deep learning; machine learning; text mining/topic modelling | online/social media/digital platform | Mexico; Spain | detection/identification | domestic violence/abuse; femicide; GBV; violence against women | direct femicide/homicide/lethality or severe-escalation relevance | model development/evaluation | model development/evaluation/validation | risk of false reassurance/false positives requires human review | Links domestic violence/abuse; femicide; GBV; violence against women with detection/identification; classification; decision support/assistance through NLP; deep learning... | Coded from available bibliographic/charting information; not reported/unclear means not inferable from the available record-level charting. |
| 115 | Alzyout M. et al. (2021) | deep learning; machine learning | not reported/unclear from available charting | Jordan; Arab world; United States | detection/identification | violence against women | indirect IPV/domestic-abuse risk-pathway relevance | model development/evaluation | model development/evaluation/validation | risk of false reassurance/false positives requires human review | Links violence against women with detection/identification; classification; decision support/assistance through deep learning; machine learning. | Coded from available bibliographic/charting information; not reported/unclear means not inferable from the available record-level charting. |
| 116 | D. D. Awasekar et al. (2025) | LLM; NLP; machine learning; chatbot/virtual assistant | online/social media/digital platform | India | detection/identification | domestic violence/abuse | indirect IPV/domestic-abuse risk-pathway relevance | model development/evaluation | model development/evaluation/validation | risk of false reassurance/false positives requires human review | Links domestic violence/abuse with detection/identification; prediction; decision support/assistance through LLM; NLP; machine learning; chatbot/virtual assistant. | Coded from available bibliographic/charting information; not reported/unclear means not inferable from the available record-level charting. |
| 117 | Castro et al. (2019) | predictive analytics/risk prediction | not reported/unclear from available charting | Philippines | risk assessment/stratification | violence against women | indirect IPV/domestic-abuse risk-pathway relevance | survey / cross-sectional | not reported/unclear or conceptual/technical | risk of false reassurance/false positives requires human review | Links violence against women with risk assessment/stratification; prediction; decision support/assistance through predictive analytics/risk prediction. | Coded from available bibliographic/charting information; not reported/unclear means not inferable from the available record-level charting. |
| 118 | Yallico Arias T. et al. (2022) | NLP; deep learning; machine learning | not reported/unclear from available charting | Peru; United States | detection/identification; risk assessment/stratification | IPV; domestic violence/abuse; femicide; violence against women | direct femicide/homicide/lethality or severe-escalation relevance | model development/evaluation | model development/evaluation/validation | risk of false reassurance/false positives requires human review | Links IPV; domestic violence/abuse; femicide; violence against women with detection/identification; risk assessment/stratification; decision support/assistance; prevention... | Coded from available bibliographic/charting information; not reported/unclear means not inferable from the available record-level charting. |
| 119 | Gutam B.G. et al. (2025) | machine learning | online/social media/digital platform | India; United States | detection/identification | GBV; stalking; harassment | indirect IPV/domestic-abuse risk-pathway relevance | model development/evaluation | model development/evaluation/validation | risk of false reassurance/false positives requires human review | Links GBV; stalking; harassment with detection/identification; classification; decision support/assistance through machine learning. | Coded from available bibliographic/charting information; not reported/unclear means not inferable from the available record-level charting. |
| 120 | Karystianis et al. (2018) | text mining/topic modelling | healthcare/EHR/clinical records; police/legal/judicial or mortality records | Australia; United States | detection/identification; risk assessment/stratification | domestic violence/abuse; violence against women | indirect IPV/domestic-abuse risk-pathway relevance | conceptual/technical paper | not reported/unclear or conceptual/technical | risk of false reassurance/false positives requires human review | Links domestic violence/abuse; violence against women with detection/identification; risk assessment/stratification; decision support/assistance through text mining/topic... | Coded from available bibliographic/charting information; not reported/unclear means not inferable from the available record-level charting. |
| 121 | O. M. Cumbicus-Pineda et al. (2021) | NLP; deep learning; machine learning; data mining | not reported/unclear from available charting | Ecuador; United States | detection/identification | domestic violence/abuse; GBV; violence against women | indirect IPV/domestic-abuse risk-pathway relevance | model development/evaluation | model development/evaluation/validation | risk of false reassurance/false positives requires human review | Links domestic violence/abuse; GBV; violence against women with detection/identification; classification; prediction; decision support/assistance through NLP; deep learning... | Coded from available bibliographic/charting information; not reported/unclear means not inferable from the available record-level charting. |
| 122 | Ismail O. et al. (2025) | NLP; deep learning; machine learning; predictive analytics/risk prediction; chatbot/virtual assistant | online/social media/digital platform | not reported | detection/identification; risk assessment/stratification | domestic violence/abuse; GBV; violence against women; stalking; harassment | indirect IPV/domestic-abuse risk-pathway relevance | survey / cross-sectional | not reported/unclear or conceptual/technical | risk of false reassurance/false positives requires human review | Links domestic violence/abuse; GBV; violence against women; stalking; harassment with detection/identification; classification; risk assessment/stratification; prediction... | Coded from available bibliographic/charting information; not reported/unclear means not inferable from the available record-level charting. |
| 123 | Vowels et al. (2026) | LLM; chatbot/virtual assistant | online/social media/digital platform | United States | detection/identification; risk assessment/stratification | IPV | indirect IPV/domestic-abuse risk-pathway relevance | model development/evaluation | model development/evaluation/validation | risk of false reassurance/false positives requires human review | Links IPV with detection/identification; risk assessment/stratification; decision support/assistance through LLM; chatbot/virtual assistant. | Coded from available bibliographic/charting information; not reported/unclear means not inferable from the available record-level charting. |
| 124 | Dehingia et al. (2022) | machine learning | not reported/unclear from available charting | India; United States | detection/identification; screening; risk assessment/stratification | IPV; GBV | indirect IPV/domestic-abuse risk-pathway relevance | survey / cross-sectional | not reported/unclear or conceptual/technical | risk of false reassurance/false positives requires human review | Links IPV; GBV with detection/identification; screening; risk assessment/stratification; decision support/assistance; prevention through machine learning. | Coded from available bibliographic/charting information; not reported/unclear means not inferable from the available record-level charting. |
| 125 | Salehi et al. (2024) | text mining/topic modelling; EHR-based identification | healthcare/EHR/clinical records; online/social media/digital platform | Iran | detection/identification | domestic violence/abuse; violence against women | indirect IPV/domestic-abuse risk-pathway relevance | review / meta-analysis | review evidence | risk of false reassurance/false positives requires human review | Links domestic violence/abuse; violence against women with detection/identification; classification; decision support/assistance; prevention through text mining/topic modelling... | Coded from available bibliographic/charting information; not reported/unclear means not inferable from the available record-level charting. |

Note. Categories are not mutually exclusive. Missing or unclear relationship status, LGBTQ+/transgender relevance, violence subtype, implementation status or governance content was coded as "not reported/unclear" and was not inferred from the authors' interpretation.

# Supplementary Table 3. Grey/legal-policy and institutional source audit

These sources informed the legal, ethical, policy and governance framing. They were not counted as empirical PRISMA-ScR core records unless otherwise indicated in the manuscript.

| **Institution/body** | **Year** | **Title** | **Source type** | **Jurisdiction/scope** | **Reason for inclusion** | **Manuscript use** | **Caution/limitation** |
| --- | --- | --- | --- | --- | --- | --- | --- |
| European Union | 2016 | Regulation (EU) 2016/679 (GDPR) | Legal/regulatory source | EU | Principles, special-category data, automated decision-making, DPIA; authority: primary law / treaty / official legal source | Legal/ethical framework; Discussion; conceptual synthesis governance | Primary law; must be read with EDPB guidance |
| European Union | 2024 | Regulation (EU) 2024/1689 (EU AI Act) | Legal/regulatory source | EU | High-risk AI, human oversight, FRIA, law-enforcement risk assessment; authority: primary law / treaty / official legal source | Legal/ethical framework; Discussion; conceptual synthesis governance | Applicability depends on concrete use case and Annex III fit |
| European Union | 2024 | Directive (EU) 2024/1385 on combating violence against women and domestic violence | Legal/regulatory source | EU | Prevention, protection, support, coordination, data collection; authority: primary law / treaty / official legal source | Introduction; Legal/ethical framework; Discussion; Policy implications | Normative framework, not evidence of effectiveness |
| Council of Europe | 2011 | Convention on preventing and combating violence against women and domestic violence (Istanbul Convention, CETS No. 210) | Legal/regulatory source | Europe | Due diligence, coordinated policies, protection, prosecution, risk assessment; authority: primary law / treaty / official legal source | Introduction; Legal/ethical framework; Discussion | International treaty standard; implementation varies by state |
| European Data Protection Board | 2018 | Automated decision-making and profiling | AI governance / data-protection guidance | EU | Article 22, profiling, safeguards, meaningful information; authority: official AI/data-protection guidance | Legal/ethical framework; AI-governance map | Not sector-specific to IPV, so must be translated cautiously |
| European Data Protection Board | 2018 | Data protection impact assessments: high-risk processing | AI governance / data-protection guidance | EU | Article 35 DPIA, high-risk indicators, vulnerable subjects; authority: official AI/data-protection guidance | Legal/ethical framework; conceptual synthesis governance | Guidance predates AI Act but remains authoritative for GDPR |
| European Data Protection Board | 2020 | Guidelines 4/2019 on Article 25 Data Protection by Design and by Default | AI governance / data-protection guidance | EU | Data minimisation, privacy by design, default settings; authority: official AI/data-protection guidance | Legal/ethical framework; AI-governance map | Horizontal guidance; not violence-specific |
| European Data Protection Board | 2024 | Opinion 28/2024 on certain data protection aspects related to AI models | AI governance / data-protection guidance | EU | Lawfulness, fairness, anonymity, downstream illegality; authority: official AI/data-protection guidance | Legal/ethical framework; Discussion | Focused on AI models generally, not policing/health triage alone |
| World Health Organization | 2024 | Violence against women fact sheet | Institutional public-health source | Global | Public-health burden, human rights, prevalence; authority: official institutional report or policy source | Introduction | Fact sheet, not a technical service-delivery manual |
| World Health Organization | 2025 | Violence against women prevalence estimates, 2023 | Institutional public-health source | Global | Updated prevalence estimates; authority: official institutional report or policy source | Introduction; Discussion | Prevalence source, not risk-pathway guidance |
| World Health Organization | 2013 | Responding to intimate partner violence and sexual violence against women: WHO clinical and policy guidelines | Risk-assessment / risk-management guidance | Global | Health-sector response, survivor-centred care, coordination; authority: official risk-assessment / clinical or operational guidance | Introduction; Discussion; conceptual synthesis human review | Older, but still the official foundational guideline pending update |
| World Health Organization | 2014 | Health care for women subjected to intimate partner violence or sexual violence: a clinical handbook | Risk-assessment / risk-management guidance | Global | First-line support, clinical workflow, referral; authority: official risk-assessment / clinical or operational guidance | Discussion; conceptual synthesis human review; Medico-legal accountability | Clinical handbook, not a legal source |
| World Health Organization | 2017 | Strengthening health systems to respond to women subjected to intimate partner violence or sexual violence: a manual for health managers | Institutional public-health source; Risk-assessment / risk-management guidance | Global | Service organisation, planning, monitoring; authority: official institutional report or policy source | Discussion; conceptual synthesis multi-agency response | Operational guidance rather than empirical trial evidence |
| WHO Regional Office for Europe | 2025 | Care, courage, change: health-sector leadership in tackling violence against women and girls | Institutional public-health source | Europe | Health policy gaps, survivor-centred services, mandatory reporting concerns; authority: official institutional report or policy source | Discussion; Policy implications | Region-specific and policy-oriented |
| UN Women / UNODC | 2025 | Femicides in 2024: Global estimates of intimate partner/family member femicides | Violence/femicide policy source | Global | Latest femicide estimates; authority: official institutional report or policy source | Introduction; Discussion | Statistical source; not a prediction framework |
| UN Women / UNODC | 2022 | Statistical framework for measuring the gender-related killings of women and girls (femicide/feminicide) | Violence/femicide policy source | Global | Definitions, typology, variables; authority: official institutional report or policy source | Introduction; Methods background; Discussion | Measurement framework, not prevention-effectiveness evidence |
| FRA / Eurostat / EIGE | 2024 | EU gender-based violence survey: Key results | Institutional public-health source; Violence/femicide policy source | EU | Prevalence of violence against women in EU; authority: official institutional report or policy source | Introduction | Key-results report is concise; detail comes from 2026 report |
| FRA / EIGE / Eurostat | 2026 | EU gender-based violence survey: Evidence for policy and practice | Institutional public-health source; Violence/femicide policy source | EU | Detailed analysis incl. psychological, economic, cyber violence; authority: official institutional report or policy source | Introduction; Discussion; Policy implications | Survey evidence, not service-effectiveness evidence |
| EIGE | 2019 | A guide to risk assessment and risk management of intimate partner violence in the EU | Risk-assessment / risk-management guidance | EU | Police risk assessment, risk management, victim safety; authority: official risk-assessment / clinical or operational guidance | Discussion; conceptual synthesis distributed signals and response | Policy guidance, not proof that any tool predicts individual femicide |
| EIGE | 2019 | Risk assessment and risk management toolkit pages | Risk-assessment / risk-management guidance | EU | Lethality, repeated violence, escalating violence, coercive control, confidentiality, multi-agency framework; authority: official risk-assessment / clinical or operational guidance | Discussion; conceptual synthesis; Policy implications | Web toolkit needs careful citation as guidance, not evidence |
| EIGE | 2022 | Combating coercive control and psychological violence against women in the EU Member States | Violence/femicide policy source; Background only | EU | Coercive control, psychological violence; authority: official institutional report or policy source | Introduction; Discussion | More policy-analytic than operational |
| GREVIO | 2025 | 5th General Report on GREVIO’s Activities | Violence/femicide policy source; Domestic homicide/femicide review source | Europe | Risk assessment and risk management implementation gaps; authority: official institutional report or policy source | Legal/ethical framework; Discussion | Monitoring and compliance lens, not empirical intervention study |
| GREVIO / Council of Europe | 2025 | Thematic perspectives on the implementation of the Istanbul Convention | Violence/femicide policy source; Domestic homicide/femicide review source | Europe | Cross-cutting implementation lessons; authority: official institutional report or policy source | Discussion; Policy implications | Secondary synthesis of GREVIO themes |
| UK Home Office | 2024 | Key findings from analysis of domestic homicide reviews | Domestic homicide/femicide review source | England and Wales | Missed opportunities, risk, information-sharing, suicide, family abuse; authority: official domestic homicide/death review report | Discussion; conceptual synthesis accountability; Limitations | Not femicide-only; jurisdiction-specific |
| Domestic Abuse Commissioner | 2025 | Learning from Loss: Ensuring the lessons from domestic homicide reviews lead to change | Domestic homicide/femicide review source | England and Wales | Oversight, implementation drift, accountability; authority: official domestic homicide/death review report | Discussion; conceptual synthesis accountability; Policy implications | Public-body report, but not a legal act |
| Office of the Chief Coroner for Ontario | 2024 | Domestic Violence Death Review Committee: 2019–2020 Annual Report | Domestic homicide/femicide review source | Ontario | Lethality risk factors and recommendations; authority: official domestic homicide/death review report | Discussion; conceptual synthesis distributed signals | Outside EU; use comparatively, not as binding standard |
| European Commission | 2026 | Ending gender-based violence / Gender Equality Strategy pages | Violence/femicide policy source; Background only | EU | Implementation context, network, AI-related gender bias in policy agenda; authority: official institutional report or policy source | Introduction; Policy implications | Policy context, not primary legal authority |
| Ministero della Salute | 2025 | Violenza sulle donne page | Institutional public-health source; Background only | Italy | Public-health framing, referral, national health response references; authority: official institutional report or policy source | Introduction; Policy implications | Webpage; use as contextual national source, not as core international authority |
| Dipartimento per le Pari Opportunità | 2025 | Piano strategico nazionale sulla violenza maschile contro le donne e la violenza domestica 2025–2027 | Violence/femicide policy source; Background only | Italy | National strategic coordination; authority: official institutional report or policy source | Discussion; Policy implications | National strategy; not a substitute for EU/CoE legal framework |

# Supplementary Table 4. Database-specific search strategies

The following section reports the database-specific search strategies supplied in Supplementary_File_1_Full_Search_Strategies_FINAL.docx. Final export/search date: 28 April 2026.

## Supplementary Table 4A. Search-strategy overview

| **Database** | **Platform/interface** | Date/export date |
| --- | --- | --- |
| PubMed/MEDLINE | PubMed; MeSH + title/abstract | Final export/search date: 28 April 2026. |
| Scopus | Scopus; TITLE-ABS-KEY | Final export/search date: 28 April 2026. |
| Web of Science Core Collection | Web of Science; Topic (TS) | Final export/search date: 28 April 2026. |
| PsycINFO | APA PsycInfo / Ovid-style syntax; title/abstract/identifier or keyword-equivalent fields | Final export/search date: 28 April 2026. |
| Criminal Justice Abstracts | EBSCOhost / Criminal Justice Abstracts; title OR abstract OR subject terms | Final export/search date: 28 April 2026. |
| IEEE Xplore | IEEE Xplore Advanced Search; All metadata | Final export/search date: 28 April 2026. |
| ACM Digital Library | ACM Digital Library Advanced Search; All fields / metadata | Final export/search date: 28 April 2026. |

## Supplementary Table 4B. Full database-specific search strings

### 1. PubMed/MEDLINE

**Final search string:**

| ("Intimate Partner Violence"[Mesh] OR "Domestic Violence"[Mesh] OR "Violence Against Women"[Mesh] OR "intimate partner violence"[tiab] OR IPV[tiab] OR "domestic violence"[tiab] OR "domestic abuse"[tiab] OR "family violence"[tiab] OR "coercive control"[tiab] OR "gender-based violence"[tiab] OR "violence against women"[tiab] OR femicide[tiab] OR feminicide[tiab] OR "intimate partner homicide"[tiab] OR "domestic homicide"[tiab] OR "lethality risk"[tiab] OR "severe violence"[tiab] OR stalking[tiab]) AND ("Artificial Intelligence"[Mesh] OR "Machine Learning"[Mesh] OR "Deep Learning"[Mesh] OR "Natural Language Processing"[Mesh] OR "Decision Support Systems, Clinical"[Mesh] OR "Electronic Health Records"[Mesh] OR "Data Mining"[Mesh] OR "artificial intelligence"[tiab] OR AI[tiab] OR "machine learning"[tiab] OR "deep learning"[tiab] OR "natural language processing"[tiab] OR NLP[tiab] OR "large language model*"[tiab] OR LLM[tiab] OR algorithm*[tiab] OR "automated decision*"[tiab] OR "predictive analy*"[tiab] OR "predictive model*"[tiab] OR "risk prediction"[tiab] OR "risk stratification"[tiab] OR "decision support"[tiab] OR "clinical decision support"[tiab] OR "data mining"[tiab] OR "text mining"[tiab] OR "record linkage"[tiab] OR "data linkage"[tiab] OR "electronic health record*"[tiab] OR EHR[tiab] OR "electronic medical record*"[tiab] OR EMR[tiab] OR chatbot*[tiab] OR "digital triage"[tiab] OR "early warning"[tiab]) |
| --- |

### 2. Scopus

**Final search string:**

| TITLE-ABS-KEY ( ("intimate partner violence" OR IPV OR "domestic violence" OR "domestic abuse" OR "family violence" OR "coercive control" OR "gender-based violence" OR "violence against women" OR femicide OR feminicide OR "intimate partner homicide" OR "domestic homicide" OR "lethality risk" OR "severe violence" OR stalking) AND ("artificial intelligence" OR AI OR "machine learning" OR "deep learning" OR "natural language processing" OR NLP OR "large language model*" OR LLM OR algorithm* OR "automated decision*" OR "predictive analy*" OR "predictive model*" OR "risk prediction" OR "risk stratification" OR "decision support" OR "clinical decision support" OR "data mining" OR "text mining" OR "record linkage" OR "data linkage" OR "electronic health record*" OR EHR OR "electronic medical record*" OR EMR OR chatbot* OR "digital triage" OR "early warning") ) |
| --- |

### 3. Web of Science Core Collection

**Final search string:**

| TS=(("intimate partner violence" OR IPV OR "domestic violence" OR "domestic abuse" OR "family violence" OR "coercive control" OR "gender-based violence" OR "violence against women" OR femicide OR feminicide OR "intimate partner homicide" OR "domestic homicide" OR "lethality risk" OR "severe violence" OR stalking) AND ("artificial intelligence" OR AI OR "machine learning" OR "deep learning" OR "natural language processing" OR NLP OR "large language model*" OR LLM OR algorithm* OR "automated decision*" OR "predictive analy*" OR "predictive model*" OR "risk prediction" OR "risk stratification" OR "decision support" OR "clinical decision support" OR "data mining" OR "text mining" OR "record linkage" OR "data linkage" OR "electronic health record*" OR EHR OR "electronic medical record*" OR EMR OR chatbot* OR "digital triage" OR "early warning")) |
| --- |

### 4. PsycINFO

**Final search string:**

| (("intimate partner violence" OR IPV OR "domestic violence" OR "domestic abuse" OR "family violence" OR "coercive control" OR "gender-based violence" OR "violence against women" OR femicide OR feminicide OR "intimate partner homicide" OR "domestic homicide" OR "lethality risk" OR "severe violence" OR stalking)).ti,ab,id. AND (("artificial intelligence" OR AI OR "machine learning" OR "deep learning" OR "natural language processing" OR NLP OR "large language model*" OR LLM OR algorithm* OR "automated decision*" OR "predictive analy*" OR "predictive model*" OR "risk prediction" OR "risk stratification" OR "decision support" OR "clinical decision support" OR "data mining" OR "text mining" OR "record linkage" OR "data linkage" OR "electronic health record*" OR EHR OR "electronic medical record*" OR EMR OR chatbot* OR "digital triage" OR "early warning")).ti,ab,id. |
| --- |

### 5. Criminal Justice Abstracts

**Final search string:**

| (TI ("intimate partner violence" OR IPV OR "domestic violence" OR "domestic abuse" OR "family violence" OR "coercive control" OR "gender-based violence" OR "violence against women" OR femicide OR feminicide OR "intimate partner homicide" OR "domestic homicide" OR "lethality risk" OR "severe violence" OR stalking) OR AB ("intimate partner violence" OR IPV OR "domestic violence" OR "domestic abuse" OR "family violence" OR "coercive control" OR "gender-based violence" OR "violence against women" OR femicide OR feminicide OR "intimate partner homicide" OR "domestic homicide" OR "lethality risk" OR "severe violence" OR stalking) OR SU ("intimate partner violence" OR IPV OR "domestic violence" OR "domestic abuse" OR "family violence" OR "coercive control" OR "gender-based violence" OR "violence against women" OR femicide OR feminicide OR "intimate partner homicide" OR "domestic homicide" OR "lethality risk" OR "severe violence" OR stalking)) AND (TI ("artificial intelligence" OR AI OR "machine learning" OR "deep learning" OR "natural language processing" OR NLP OR "large language model*" OR LLM OR algorithm* OR "automated decision*" OR "predictive analy*" OR "predictive model*" OR "risk prediction" OR "risk stratification" OR "decision support" OR "clinical decision support" OR "data mining" OR "text mining" OR "record linkage" OR "data linkage" OR "electronic health record*" OR EHR OR "electronic medical record*" OR EMR OR chatbot* OR "digital triage" OR "early warning") OR AB ("artificial intelligence" OR AI OR "machine learning" OR "deep learning" OR "natural language processing" OR NLP OR "large language model*" OR LLM OR algorithm* OR "automated decision*" OR "predictive analy*" OR "predictive model*" OR "risk prediction" OR "risk stratification" OR "decision support" OR "clinical decision support" OR "data mining" OR "text mining" OR "record linkage" OR "data linkage" OR "electronic health record*" OR EHR OR "electronic medical record*" OR EMR OR chatbot* OR "digital triage" OR "early warning") OR SU ("artificial intelligence" OR AI OR "machine learning" OR "deep learning" OR "natural language processing" OR NLP OR "large language model*" OR LLM OR algorithm* OR "automated decision*" OR "predictive analy*" OR "predictive model*" OR "risk prediction" OR "risk stratification" OR "decision support" OR "clinical decision support" OR "data mining" OR "text mining" OR "record linkage" OR "data linkage" OR "electronic health record*" OR EHR OR "electronic medical record*" OR EMR OR chatbot* OR "digital triage" OR "early warning")) |
| --- |

### 6. IEEE Xplore

**Final search string:**

| ("All Metadata":"intimate partner violence" OR "All Metadata":"domestic violence" OR "All Metadata":"domestic abuse" OR "All Metadata":"family violence" OR "All Metadata":"coercive control" OR "All Metadata":"gender-based violence" OR "All Metadata":"violence against women" OR "All Metadata":"femicide" OR "All Metadata":"feminicide" OR "All Metadata":"intimate partner homicide" OR "All Metadata":"domestic homicide" OR "All Metadata":"lethality risk" OR "All Metadata":"severe violence" OR "All Metadata":"stalking") AND ("All Metadata":"artificial intelligence" OR "All Metadata":"machine learning" OR "All Metadata":"deep learning" OR "All Metadata":"natural language processing" OR "All Metadata":"NLP" OR "All Metadata":"large language model" OR "All Metadata":"LLM" OR "All Metadata":"algorithm" OR "All Metadata":"automated decision" OR "All Metadata":"predictive analytics" OR "All Metadata":"predictive model" OR "All Metadata":"risk prediction" OR "All Metadata":"risk stratification" OR "All Metadata":"decision support" OR "All Metadata":"clinical decision support" OR "All Metadata":"data mining" OR "All Metadata":"text mining" OR "All Metadata":"record linkage" OR "All Metadata":"data linkage" OR "All Metadata":"electronic health record" OR "All Metadata":"EHR" OR "All Metadata":"electronic medical record" OR "All Metadata":"EMR" OR "All Metadata":"chatbot" OR "All Metadata":"digital triage" OR "All Metadata":"early warning") |
| --- |

### 7. ACM Digital Library

**Final search string:**

| AllField: ("intimate partner violence" OR IPV OR "domestic violence" OR "domestic abuse" OR "family violence" OR "coercive control" OR "gender-based violence" OR "violence against women" OR femicide OR feminicide OR "intimate partner homicide" OR "domestic homicide" OR "lethality risk" OR "severe violence" OR stalking) AND AllField: ("artificial intelligence" OR AI OR "machine learning" OR "deep learning" OR "natural language processing" OR NLP OR "large language model*" OR LLM OR algorithm* OR "automated decision*" OR "predictive analy*" OR "predictive model*" OR "risk prediction" OR "risk stratification" OR "decision support" OR "clinical decision support" OR "data mining" OR "text mining" OR "record linkage" OR "data linkage" OR "electronic health record*" OR EHR OR "electronic medical record*" OR EMR OR chatbot* OR "digital triage" OR "early warning") |
| --- |

Supplementary Table 5. Reports sought for retrieval but not retrieved

This table lists the 14 reports for which full texts were sought but not retrieved despite legal and institutional access attempts.

Note. Classification is based only on bibliographic metadata and titles. These reports were not included in full-text eligibility assessment and were not incorporated into the core evidence map because full texts could not be retrieved.

| **No.** | **Author(s), year** | **Title** | **Metadata-level topic cluster** | **Potential relevance to the review** | **Retrieval status / full-text assessment** |
| --- | --- | --- | --- | --- | --- |
| 1 | Lucea et al., 2025 | Homicides of Pregnant Women: Artificial Intelligence Detects Partner Violence and System Interaction. | Femicide / homicide / system interaction | Potentially relevant to AI-supported recognition of partner-violence signals in lethal or pregnancy-associated contexts. | Full text not retrieved despite legal and institutional access attempts; not assessed at full-text level. |
| 2 | Kafka et al., 2023 | Intimate partner violence and suicide mortality: a cross-sectional study using machine learning and natural language processing of suicide data from 43 states. | Suicide mortality / NLP / violent-death data | Potentially relevant to AI/NLP detection of IPV circumstances in mortality data. | Full text not retrieved despite legal and institutional access attempts; not assessed at full-text level. |
| 3 | Kafka et al., 2023 | Detecting intimate partner violence circumstance for suicide: development and validation of a tool using natural language processing and supervised machine learning in the National Violent Death Reporting System. | Suicide data / NLP / supervised machine learning | Potentially relevant to supervised NLP identification of IPV circumstances in violent-death datasets. | Full text not retrieved despite legal and institutional access attempts; not assessed at full-text level. |
| 4 | Walklate, 2025 | 'Boundary Objects' and 'Black Boxes': Theory Informed Prevention for Femicide. | Femicide prevention / theory / black-box systems | Potentially relevant to conceptual and governance issues around black-box tools in femicide prevention. | Full text not retrieved despite legal and institutional access attempts; not assessed at full-text level. |
| 5 | Behnam et al., 2024 | SAFETY FIRST: Electronic Health Record Integration of Intimate Partner Violence Screening and Response in Primary Care. | EHR / primary care / IPV screening | Potentially relevant to integration of IPV screening and response in healthcare records. | Full text not retrieved despite legal and institutional access attempts; not assessed at full-text level. |
| 6 | Rasel and Chowdhury, 2024 | A machine learning-based domestic violence prediction and Android application-based domestic violence prevention assistance system. | ML prediction / mobile application / DV assistance | Potentially relevant to application-based domestic-violence prediction and prevention support. | Full text not retrieved despite legal and institutional access attempts; not assessed at full-text level. |
| 7 | Zeidan et al., 2022 | Estimating the prevalence of intimate partner violence at an urban hospital before and during the COVID-19 pandemic using a novel natural language processing algorithm. | Hospital setting / NLP / IPV prevalence | Potentially relevant to NLP-based identification of IPV in hospital data. | Full text not retrieved despite legal and institutional access attempts; not assessed at full-text level. |
| 8 | Echeburua et al., 2010 | Severe Intimate Partner Violence Risk Prediction Scale-Revised. | IPV risk prediction / risk scale | Potentially relevant to severe IPV risk assessment, although AI/algorithmic eligibility could not be confirmed without full text. | Full text not retrieved despite legal and institutional access attempts; not assessed at full-text level. |
| 9 | Paramasivan et al., 2025 | A Lightweight Neural Network Tool for the Detection of Domestic Abuse and Harassment Against Women to Improve Reporting and Deter Offenders. | Neural network / domestic abuse / harassment detection | Potentially relevant to AI-based detection of domestic abuse and reporting support. | Full text not retrieved despite legal and institutional access attempts; not assessed at full-text level. |
| 10 | Palacios-Hidalgo and Huertas-Abril, 2024 | AIALL and queer language education to prevent gender-based violence: Using artificial intelligence for lesson planning. | AI education / queer language education / GBV prevention | Potentially relevant to AI-supported prevention education, although direct IPV risk-pathway eligibility could not be confirmed without full text. | Full text not retrieved despite legal and institutional access attempts; not assessed at full-text level. |
| 11 | Hindin and Wilson, 2015 | Developing a Predictive Model to Inform Gyn Health Promotion in Women who Experience Intimate Partner Violence. | Predictive model / gynecological health / IPV | Potentially relevant to predictive modelling for health promotion among women exposed to IPV. | Full text not retrieved despite legal and institutional access attempts; not assessed at full-text level. |
| 12 | Eaton et al., 2011 | A Predictive Model to Help Identify Intimate Partner Violence Among Older Women Based on Phone Calls and Diagnoses. | Predictive model / phone calls / diagnoses / older women | Potentially relevant to healthcare-data-based IPV identification. | Full text not retrieved despite legal and institutional access attempts; not assessed at full-text level. |
| 13 | Thakur, 2025 | Intersectionality and the problems of using artificial intelligence to address online gender-based violence and gendered disinformation. | AI governance / online GBV / intersectionality | Potentially relevant to intersectional risks and limitations of AI responses to online gender-based violence. | Full text not retrieved despite legal and institutional access attempts; not assessed at full-text level. |
| 14 | Souza Francisco et al., 2025 | Challenges in Extracting Forensic Data on Sexual and Gender-Based Violence Victims. | Forensic data extraction / sexual and gender-based violence | Potentially relevant to forensic-data extraction in gender-based violence, although direct IPV/AI eligibility could not be confirmed without full text. | Full text not retrieved despite legal and institutional access attempts; not assessed at full-text level. |
